# Following the Structural Changes of Iron Oxides during Reduction under Transient Conditions

**DOI:** 10.1002/cssc.202401045

**Published:** 2024-09-03

**Authors:** Lukas Braun, Jonas Spielmann, Dmitry E. Doronkin, Carola Kuhn, Aleksandr Maliugin, Dmitry I. Sharapa, Isabel Huck, Jianing Bao, Steffen Tischer, Felix Studt, Olaf Deutschmann, Ulrike I. Kramm, Jan‐Dierk Grunwaldt

**Affiliations:** ^1^ Institute for Chemical Technology and Polymer Chemistry (ITCP) Karlsruhe Institute of Technology Engesserstr. 20 76131 Karlsruhe Germany; ^2^ Eduard-Zintl Institute for Inorganic and Physical Chemistry Catalysts and Electrocatalysts Technical University of Darmstadt Otto-Berndt Street 3 64287 Darmstadt Germany; ^3^ Institute of Catalysis Research and Technology (IKFT) Karlsruhe Institute of Technology Hermann-von-Helmholtz-Platz 1 76344 Eggenstein-Leopoldshafen Germany

**Keywords:** Energy conversion, Circularity, Iron reduction, Mössbauer spectroscopy, Synchrotron X-ray absorption spectroscopy and diffraction

## Abstract

Iron is considered as attractive energy carrier in a carbon‐free, circular energy economy. The reduction of iron oxide is crucial for its application as a metal fuel as it determines the efficiency of the cycle. Temperature programmed reduction of α‐Fe_2_O_3_ was monitored by complementary X‐ray absorption spectroscopy (XAS) and diffraction (XRD) to obtain the phase composition with high time resolution. Synchrotron Mössbauer spectroscopy (SMS) was additionally employed due to its high sensitivity to the different iron species. Theoretical calculations of surface and bulk adsorption processes were performed to establish the potential reaction pathways and the corresponding energy barriers. A kinetic particle model was then developed to bridge the experimental data and theoretical calculations, which reproduced the reduction onset and behavior. The reduction process was found to be strongly dependent on the heating rate in terms of the reduction window and the observed intermediate species. We propose that a core‐shell mechanism determines the reaction by forming an iron layer which subsequently hinders diffusion of water out of the porous particles leading to some unreduced FeO at high temperature. This study demonstrates the need for complementary methods for describing complex heterogeneous systems and overcoming the chemical sensitivity limitations of any single method.

## Graphical abstract

The iron/iron oxide system will play a vital role as an energy carrier in a carbon‐free circular energy economy by enabling energy storage via the reduction of iron oxide and energy release via combustion of reduced iron. Various synchrotron‐based characterization techniques, supported by theoretical calculations and kinetic modelling, were applied to evaluate the iron oxide reduction process and investigate the changes in the mechanism depending on the conditions.

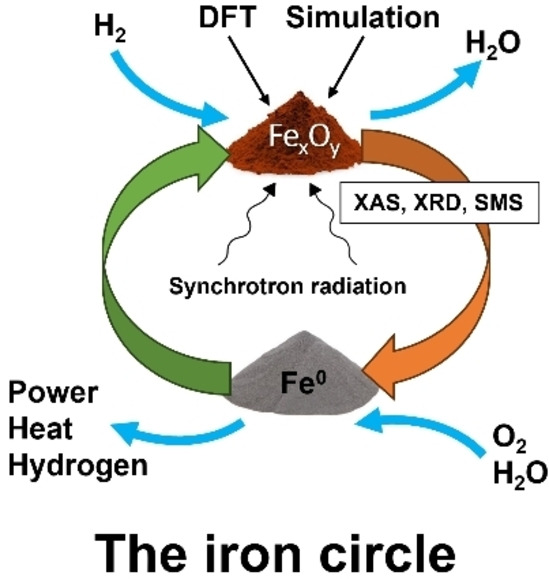

## Introduction

The combustion of fossil fuels for energy production in the current day leads to large emissions of CO_2_, methane, and other greenhouse gases, contributing to global warming and climate change. In 2015, 196 countries adopted the Paris agreement and pledged to decrease their greenhouse gas emissions to mitigate the effects of the climate change by limiting the average global temperature increase to 1.5 °C above pre‐industrial levels.^[1]^ Achieving this goal will require a complete transition of our current energy production away from fossil towards clean and renewable energy. In principle, the current energy production from coal and natural gas fired power plants can be replaced by renewable energy sources such as wind and solar power. However, their volatile nature and strong dependence on the location of production make it obligatory to bridge sites of energy production and energy demand by suitable energy carriers, ensuring a stable power supply.[^2^, ^3^, ^4^] Conventional concepts, such as mechanical storage or pumped hydroelectric energy storage, cannot be scaled up to the needed capacities in the TWh regime, whereas chemical storage media e. g. metals, can fulfill this demand.^[5]^ P. Julien and J. Bergthorson^[6]^ analyzed the potential of various metals as future energy carriers and identified characteristics that need to be fulfilled. An optimal energy carrier should have a high energy density, be stable in its reduced form over long storage periods and can be transported safely over long distances. In particular, a high volumetric density becomes important as it will reduce the space required for storage and transport, which is one of the main disadvantages of hydrogen or hydrogen derivates as an energy carrier.[^7^, ^8^] Additionally, the material should be naturally abundant and recyclable.

**Figure 1:**
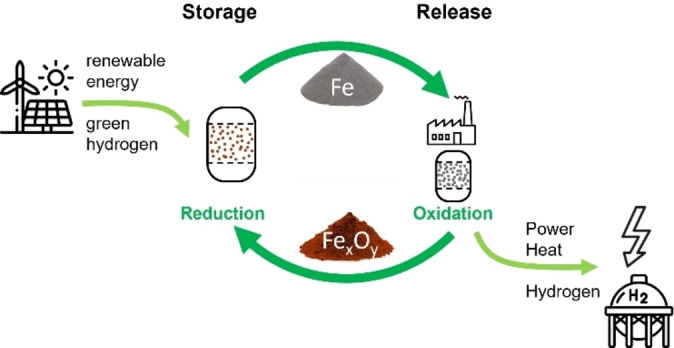
Principle of a metal fuel circle for storage of energy and regain with the example of iron (see also ref [19]).

Energy storage is realized via reduction of the storage medium, while it is burned (*i. e*. oxidized) for the energy release, as shown schematically in Figure 1. Reduction can be achieved either directly or with an intermediate step of hydrogen production. The oxidation can be carried out either in air to produce electricity and heat, or with the addition of water to produce hydrogen once again. The described cycle should be repeatable many times, ideally infinitely, and no greenhouse gases should be released during reduction and reoxidation. Given these challenges, as well as price and availability, iron, aluminum, and silicon have been identified as the most attractive energy carriers for a future large‐scale application. Silicon (20.8 kWh ⋅ L^−1^)^[9]^ suffers from the fact that it cannot be fully reduced by hydrogen (H_2_), leading only to silicon monoxide SiO, which significantly decreases the overall efficiency.[^9^, ^10^] Aluminum possesses the highest volumetric energy density (23.5 kWh ⋅ L^−1^) of the three, but has two significant drawbacks.^[11]^ First, the handling of alumina powder requires special care and safety precautions, due to the high deflagration index of small particles presenting a serious explosion hazard.[^12^, ^13^] Secondly, aluminum is expected to burn in a vapor‐phase droplet combustion mode because the flame temperature vastly exceeds the boiling point of the metal.[^13^, ^14^] This will lead to an increased formation of nanoparticles, which are generally more difficult to collect and to implement in continuous processes with high flows.[^13^, ^15^] Although iron has a lower energy density (16.7 kWh ⋅ L^−1^)^[11]^ compared to these two metals, it has several physicochemical properties that are advantageous for energy storage. Iron powder is easy to store and transport. Its deflagration index is much lower than that of aluminum and even lower than that of coal, resulting in simpler and safer operation.[^12^, ^16^, ^17^] Furthermore, due to its high density, lower heating value[^18^, ^19^] and high combustion temperature of 2200 K, similar to standard carbon‐based fuels, iron powders could be used in standard combustion units with only a few modifications.^[20]^ The oxidized particles from the combustion would be collected in storage tanks. Upon complete oxidation, hematite (α‐Fe_2_O_3_) is formed, with iron in a formal oxidation state of +III. The metals can then be recycled (see Figure 1), helping us to move away from the current single‐use economy to a regenerative circular economy.

In fact, iron powder exhibits similar physical properties to pulverized coal, which allows the use of the metal in retrofitted power plants. Existing coal‐fired power plants need only minor adjustments, resulting from the lower heating value, but higher volumetric density of iron powder as compared to coal.[^19^, ^21^, ^22^] Since some of the supplementary systems required in coal‐fired power plants, such as a desulfurization system, and parts of the exhaust gas aftertreatment can be omitted a higher efficiency is generally possible when using iron powder as fuel.^[21]^ By combining green hydrogen as the reducing agent and such retrofitted power plants for energy generation, a carbon‐free energy circle with decentralized facilities as given schematically in Figure 1 can be realized. Decoupling the production and consumption of the metal fuels allows for greater flexibility while maintaining a closed chemical cycle. For a circular economy within the chemical industry it will be crucial to optimize spare resources and minimize waste production.[^23^, ^24^] This will be especially important for very energy‐intensive industrial processes, such as steel production.[^25^, ^26^, ^27^, ^28^] Carbon capturing, the associated transport, and storage are less attractive than avoiding CO_2_ emissions in the first place due to high costs and safety concerns.^[29]^ Green hydrogen produced by water electrolysis can be used as a carbon‐neutral replacement for the currently dominant blast furnace route in steel production.^[27]^ So far the so‐called *hydrogen direct reduction of iron oxide* (H‐DRI) process has only been partially installed in industrial plants, but the topic is constantly developing due to its importance for the future.[^30^, ^31^, ^32^] Nevertheless, the first plants for direct reaction with green hydrogen and associated steel production are in the late stages of design or construction and are expected to be operational within the next few years.[^33^, ^34^] Considering that the steel industry is responsible for about 7 % of the total CO_2_ emissions, the potential for the H‐DRI process to reduce carbon emissions in this sector is massive.^[35]^

For closed‐loop energy storage, the efficiency of the reduction step is crucial to be economically viable, so a detailed understanding of the iron reduction mechanism and knowledge of the phase composition during the process and its dependence on technically important parameters such as heating rates, particle sizes or gas composition is critical. Due to the complexity of the multistep reduction process of α‐Fe_2_O_3_ through different intermediates (e. g. Fe_3_O_4_ and FeO) the exact mechanism is still a matter of debate. In this special issue, we now focus on this first part of the iron energy storage circle. Using complementary *in situ* X‐ray absorption spectroscopy (XAS) combined with X‐ray diffraction (XRD) and *in situ* Synchrotron Mössbauer spectroscopy (SMS), we have been able to identify the phase content, oxidation state, and chemical environment of Fe species during the reduction of Fe_2_O_3_, resulting in a better understanding of the mechanisms involved. All techniques including Mössbauer spectroscopy have been conducted employing synchrotron radiation to obtain sufficient time resolution. The observations made are additionally verified by density functional theory (DFT) calculations and simulated based on the corresponding microkinetic models using a particle model.

## Results and Discussion

### Ex Situ Characterization

In this work, we compare the structural changes of iron oxide (hematite) particles during the reduction process using two different heating rates. Before going into detail of the *in situ* experiments, in the following we will summarize the most relevant physicochemical characteristics of the starting material.

Scanning electron microscopy (SEM) was used to analyze the morphology of the particle. Figure S1 shows the α‐Fe_2_O_3_ sample with an average particle size of 3 μm. The particles had a uniform porous structure. Aggregates ranging from 1.5 μm to about 5 μm were found with an average size of 3.1 μm±0.2 μm as determined by SEM. No dense, spherical particles were observed, but rather broken‐up spheres. To further investigate the textural properties, N_2_‐physisorption (BET) as well as *ex situ* XRD were performed, and the results are summarized in Table 1. BET provided a specific surface area slightly below 10 m^2^ ⋅ g^−1^ and a low porosity with a small pore volume. XRD was used for phase identification and estimation of the crystallite phase (Figure S2). All reflections expected for α‐Fe_2_O_3_ (ICSD collection code 164010) were present, as shown in Figure S2. The full line width at half maximum (FWHM) of the (104)‐reflection at 33.1° was used to estimate the size of crystallite grains (cf. Table 1) via the Scherrer equation (Figure S2). As expected, the size was significantly smaller than the particle size observed in Figure S1. This can be explained by the fact that single particles consist of several crystallite grains.

**Table 1:** BET specific surface area, porosity, and crystallite size (extracted from BET and XRD, respectively).

Sample	BET specific surface area (m^2^ ⋅ g^−1^)	Pore volume (cm^3^ ⋅ g^−1^)	Crystallite size (nm)^[a]^	Crystallite size (nm)^[b]^
Fe_2_O_3_	8.7	0.027	29	26

[a] Calculated from XRD data using Scherrer′s equation using the [104] reflection of α‐Fe_2_O_3_. [b] Estimated from the BET data (see SI) by relating the specific surface area to the reciprocal crystallite grain size, using the density of Fe_2_O_3_, cf. ref.[36].

Mössbauer spectroscopy (MS) was able to confirm the purity of the powder, showing only the characteristic sextet signal associated with α‐Fe_2_O_3_. The spectrum is shown in Figure S3, and the parameters are reported in Table S2. A portion of the signal (ca. 14 %) shows a slightly smaller magnetic hyperfine splitting (49.9 vs 51.4 T), signaling the existence of a nanomaterial with a not fully developed internal magnetic field, which was considered in the fitting process accordingly.^[37]^

### In Situ XAS and XRD

The X‐ray absorption near edge spectra (XANES) were recorded during the *in situ* reduction of diluted α‐Fe_2_O_3_ microparticles with 5 % H_2_ at constant heating rates up to 750 °C. Spectra recorded during the reduction (Figure S4 and Figure S5), as well as the spectra of the references (Figure S6) are displayed in the supporting information. All starting spectra showed exactly the same features as α‐Fe_2_O_3_ (the starting material used), although with some dampening of peak and valley features. This dampening was slightly differing for different experiments (noticeable by a slightly lower white line intensity) and was therefore not related to the chemistry of the samples, but only to the non‐uniform packing of the samples with pinholes, which is inevitable for the given material (highly diluted microparticles in a powder matrix), but not ideal for transmission spectroscopy.^[38]^ Nevertheless, since the starting and end states (all features correspond to the spectrum of Fe^0^ with the corresponding dampening) were well defined and the sample geometry did not change during the experiment, their spectra could be used for linear combination analysis (LCA) instead of the spectra of the reference compounds. The same approach could not be used for the intermediate species; here spectra of pelletized, uniformly diluted reference compounds were used to account for intermediate species during the LCA. However, in our specific case with relatively small contributions from intermediate states, neglecting the moderate dampening effect for intermediates did not result in any significant misfit, with fit residuals always close to the zero line.

**Figure 2:**
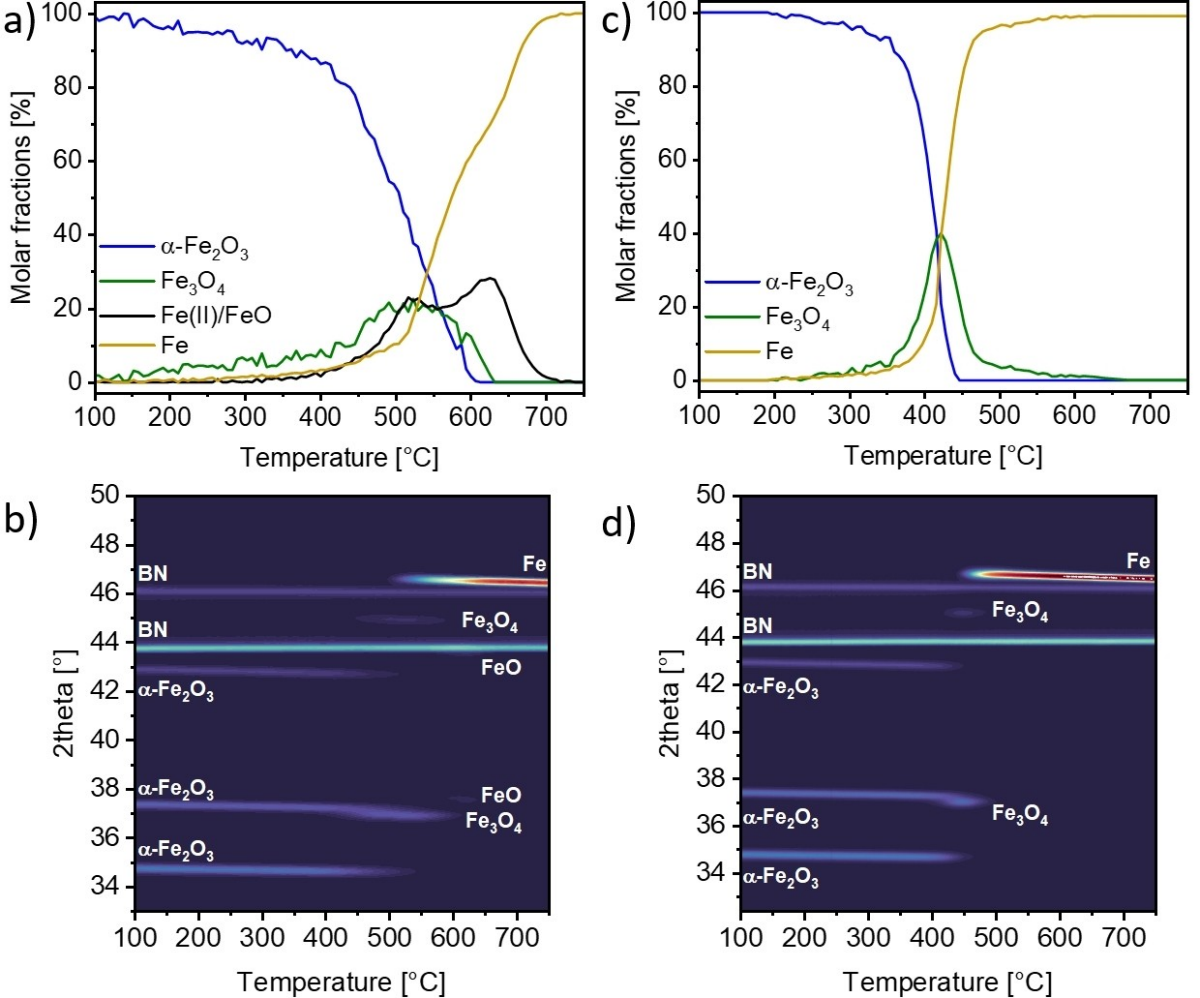
(a) Reduction profiles obtained from the LCA of in situ XANES of α‐Fe_2_O_3_ measured during temperature programmed reduction (TPR) at 15 K ⋅ min^−1^ ramp rate and (b) the corresponding XRD profiles. (c) Reduction profiles obtained from the LCA of in situ XANES of α‐Fe_2_O_3_ measured during TPR at 2 K ⋅ min^−1^ ramp rate and (d) the corresponding XRD profiles.

The results of the linear combination analysis of the TPR of α‐Fe_2_O_3_ with an average particle size of 3 μm at a heating rate of 15 K min^−1^ are shown in Figure 2a. As described in the experimental part, *in situ* XRD was measured at the same time as quick‐scanning extended X‐ray absorption fine structure (QEXAFS). The time for each XAS spectrum was less than one second, while each diffractogram required approximately two seconds to collect. This was considerably faster than the heating of the sample, resulting in a negligible error in the temperature scale. The reaction onset was observed at around 340 °C and the hematite species were fully converted around 612 °C. Nearly simultaneous to the direct conversion to Fe^0^, the reduction to magnetite (Fe_3_O_4_) occurred as an intermediate species. A maximum of 44 %_mol_ magnetite was detected at 523 °C. Simultaneous with magnetite, a fraction of wüstite (FeO) could be formed according to the LCA. However, this intermediate phase is thermodynamically unstable at this temperature (Figure S19) and can most likely also be traced back to Fe_3_O_4_ (as also indicated by SMS). We attribute this to the error in the XANES data processing, as both of these iron species contain Fe with an oxidation state of +2. XANES is highly sensitive towards the oxidation state and local structure, which makes it likely to misinterpret Fe_3_O_4_ as a mixture of FeO and Fe_2_O_3_.[^39^, ^40^] The actual FeO formation should occur at 570 °C or above, within its thermodynamic stability window (Figure S19).^[41]^ Therefore, the exact starting point for FeO formation could not be determine by XAS alone. A maximum fraction of 28 %_mol_ wüstite was observed at 625 °C and full conversion towards metallic iron at 720 °C.

The contour plot of the X‐ray diffraction patterns against the temperature is shown in Figure 2b. The reflections of α‐Fe_2_O_3_ appear at 2Θ=34.9°, 37.5°, 43.1°, and 45.8° at the energy used (λ_beam_=1.6102 Å, E_beam_=7700 eV).[^40^, ^41^] The contour diagram of the XRD patterns shows reflections of the species identified in the LCA analysis of XANES, in addition to the boron nitride dilutant. A steady decrease in intensity of the α‐Fe_2_O_3_ reflections could be found until 540 °C, above this temperature no reflections of this phase could be detected, indicating complete disappearance of hematite. Upon reaching temperature of 445 °C, the conversion towards magnetite (Fe_3_O_4_) was observed, as evidenced by the signals at 37.1° and 45.1°. The transformation of Fe_3_O_4_ was completed at 612 °C. First amounts of metallic iron were detected by a reflection at 46.7° at 495 °C onwards, with increasing intensity as the reaction continued. The boron nitride signals at 43.8° and 46.1° can superimpose with the α‐Fe_2_O_3_ signal at 45.8° and the FeO signal at 44.0°. This proved to be a problem in the XRD analysis, as the remaining FeO reflection at 37.8° is superimposed by the much more intense hematite signal at this position. With the help of multiple peak fitting with a Gaussian model (see Figure S8 and Table S1) the reflections could be deconvoluted. In the temperature range of 593 °C to 635 °C a signal of wüstite could be detected, also demonstrating the potential misinterpretation of the LCA results at lower temperatures, as described above.

The effect of different heating rates during the non‐isothermal reduction on the reduction rate and behavior was tested by decreasing the rate to 2 K ⋅ min^−1^. LCA results are displayed in Figure 2c with the corresponding XRD contour plot is given in Figure 2d. The start of the reaction was estimated to appear between 300 °C and 320 °C, where the onset of the conversion of hematite towards magnetite and metallic iron was observed by XAS. A very fast (in the temperature domain) reduction of hematite, led to full conversion at 453 °C. Magnetite was formed as an intermediate with a maximum of 40 %_mol_ at 421 °C, which is then fully reduced around 550 °C. The LCA was performed without including the FeO (wüstite) phase, as no traces were detected by the other analytical techniques and its unstable nature at the specific reaction conditions. Fits including FeO as a potential reference were also performed, but no improvement in the fit residuals was observed.

XRD patterns (Figure 1d) showed Fe_3_O_4_ around 418 °C, while first reflections of metallic iron could be seen at 438 °C. The hematite signal disappeared approximately at 462 °C, and the diffractograms indicate a complete reduction of the remaining Fe_3_O_4_ to Fe at 498 °C. As a result, it can be concluded that the reduction was initiated at significantly lower temperatures when the heating rate was decreased from 15 K ⋅ min^−1^ to 2 K ⋅ min^−1^, which was probably caused by a change in the reduction mechanism. This shift in the onset of reduction was also found in thermogravimetric experiments under similar conditions.[^42^, ^43^] The XRD patterns validate the viability of the LCA analysis of XANES without the inclusion of FeO, as no traces of FeO were visible in the diffractograms, which would otherwise appear at 37.8° and 44.0°. The XRD results reflect the reduction behavior determined by XAS very well and the transition points also match well with a maximum deviation of 10–20 °C. It could therefore help to determine which species occurred, as the method was not so much influenced by the oxidation state as XAS. It should be noted that only the extended crystalline domains of each phase could be analyzed by XRD, and the amorphous parts could not be evaluated by this method. Therefore, the exact transition points could be misinterpreted by XRD and need to be cross‐referenced with other methods (e. g. XAS, Mössbauer spectroscopy), as done in the current work. A detailed discussion of the mechanisms we propose for the α‐Fe_2_O_3_ reduction will be given later.

### In Situ Mössbauer Spectroscopy

^57^Fe Mössbauer spectroscopy (MS) is highly sensitive to changes in oxidation and spin states and has, in contrast to XRD, the capability of identifying both amorphous and crystalline phases. Thus, this technique was chosen to check for reproducibility and robustness of the XAS and XRD analysis. To enable *in situ* Mössbauer spectroscopy with reasonable acquisition times, we used partially enriched α‐Fe_2_O_3_ particles, as described in the experimental part. Enrichment in ^57^Fe and the absence of a non‐resonant background for Mössbauer measurements with a synchrotron radiation source allows to bring the measurement time down to minutes, as compared to hours and days for natural iron in laboratory based Mössbauer spectroscopy. Moreover, a very similar setup as employed during XAS/XRD measurements for reduction could be used (cf. Setup S1 and S2 in the supporting information). Beginning of test (BoT) and End of test (EoT) spectra with longer measurement times were recorded to confirm the purity of the starting material and completeness of the reduction at EoT. The spectra recorded during the reduction are shown in Figure S13 a+v and Figure S14 a+s in the ESI, for the runs with 15 K ⋅ min^−1^ and 2 K ⋅ min^−1^, respectively.

For the evaluation of the spectra, knowledge of the temperature dependent changes of the hyperfine interaction parameters (HIPs) of the expected species occurring during the reduction reaction is necessary. Values of center shift (CS), quadrupole splitting (QS), and magnetic hyperfine splitting (H) are known from literature for pure systems at various temperatures.[^44^, ^45^, ^46^, ^47^, ^48^, ^49^, ^50^, ^51^, ^52^, ^53^, ^54^] From our experience we know that HIPs obtained under *in situ* conditions (e. g. during oxidation or reduction reactions) corresponded well to those of the pure components without an ongoing reaction.

Thus, these parameters served as orientation for the evaluation of the spectra. Moreover, the contribution of the nanomaterial was approximated by a larger Lorentz‐contribution to the signal line widths of the bulk component to balance the complexity of the fitting models.

Figure S15 and Figure S16 in the ESI compare the HIPs obtained in this study with the literature values. Overall, a good agreement was observed. Only the values for the magnetic hyperfine field (Figure S15b & Figure S16b, ESI) of iron and magnetite differed slightly from literature. Parameters found for both experiments with heating rates of 2 and 15 K ⋅ min^−1^ were consistent within the margin of error, as shown by the comparison of the HIPs shown in Figure S17.

**Figure 3:**
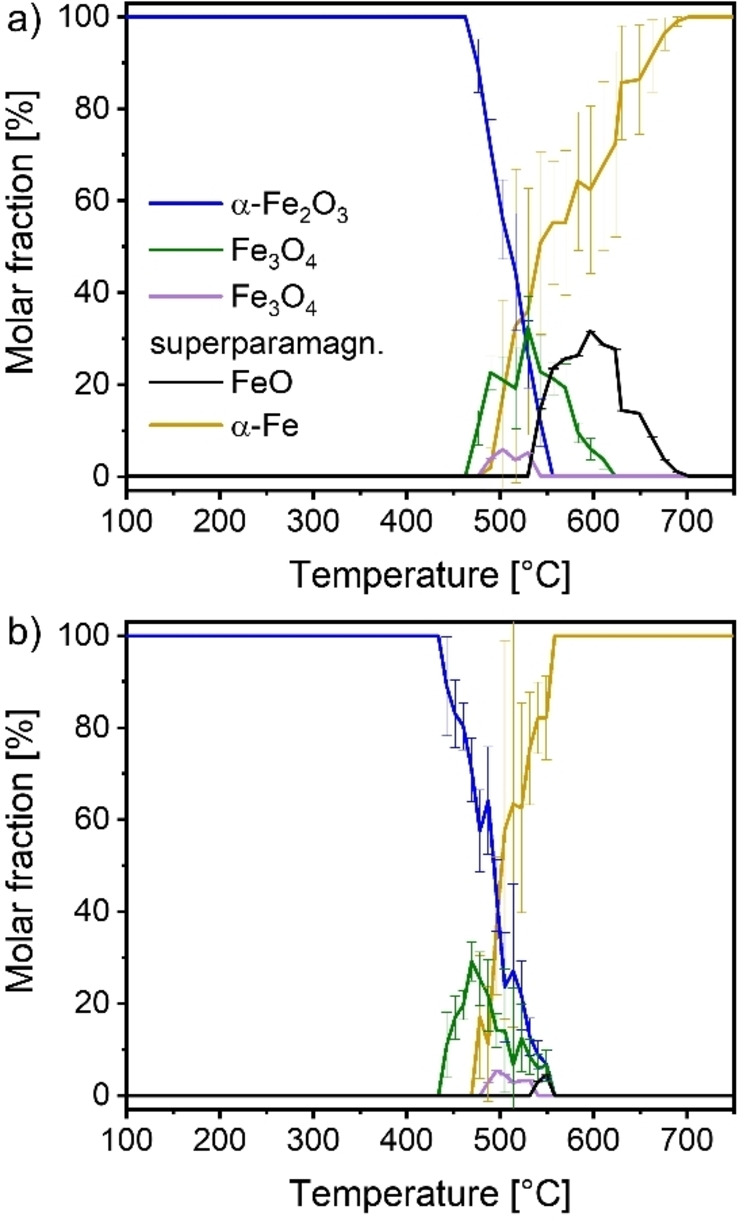
Composition obtained from Mössbauer spectra recorded during TPR with (a) 15 K ⋅ min^−1^ and (b) 2 K ⋅ min^−1^ ramp rates of the partially enriched α‐Fe_2_O_3_ powder diluted 1 : 10 with BN. A direct comparison of Mössbauer vs XAS results is shown in the supporting information in Figure S9 and Figure S10.

Figure 3 shows the composition profiles obtained during reduction with heating rates of 2 K ⋅ min^−1^ and 15 K ⋅ min^−1^. Overall, reduction with a heating rate of 15 K ⋅ min^−1^ appeared over a temperature interval of 240 °C but was very fast time‐wise with only 16 minutes from beginning to end of reduction. For each individual spectrum we estimated an error in temperature of 7.5 °C, the reduction with a heating rate of 2 K ⋅ min^−1^ spanned a total of 124 °C which corresponded to a time interval of 1 h 5 min from beginning to end, the estimated error of given temperature values was 5 °C. In both experiments, the reduction started with the formation of Fe_3_O_4_, followed by the appearance of α‐Fe. FeO was further found once the temperature had been risen to about 550 °C.

For the heating rate of 15 K ⋅ min^−1^, the beginning of the reduction was observed at 463 °C. Initially, large amounts of Fe_3_O_4_ were formed with a maximum at 530 °C and 32±7 %_mol_. α‐Fe and FeO were identified from 490 °C and 544 °C onwards, respectively. After reaching its maximum, Fe_3_O_4_ was consumed in favor of α‐Fe and FeO formation and completely disappeared at 624 °C. FeO reached a maximum at 597 °C with 31.6±1.0 %_mol_ and decreased then in content until full reduction was achieved at 703 °C.

In case of the 2 K ⋅ min^−1^ heating rate (Figure 3b), the reduction started at 434 °C. Like for heating with 15 K ⋅ min^−1^, large amounts of Fe_3_O_4_ were formed initially with a maximum of 29.2±4.2 %_mol_ at 469 °C. Afterwards, both α‐Fe_2_O_3_ and Fe_3_O_4_ were consumed until the reduction was completed at 558 °C. FeO appeared around 550 °C in very small amounts of up to 4.4 %_mol_.

**Figure 4:**
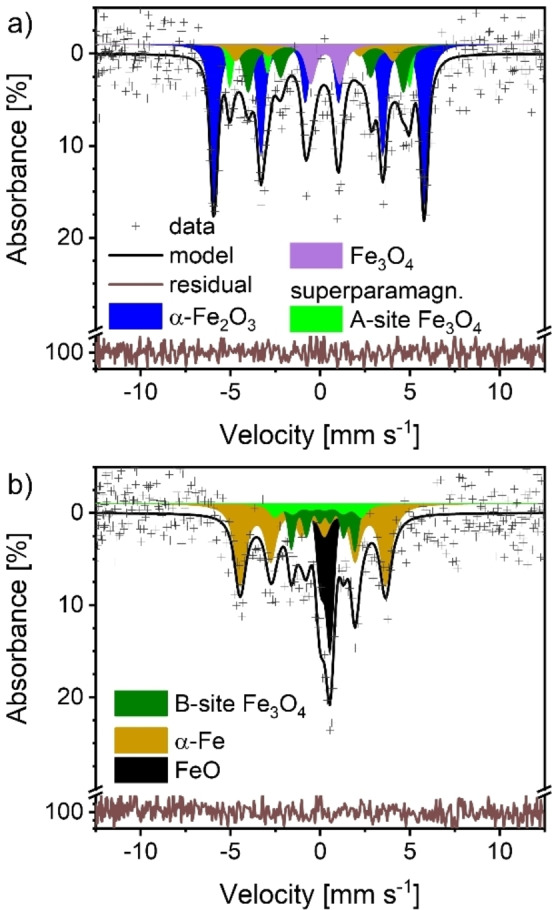
Exemplary *in situ* Mössbauer spectra obtained during 15 K ⋅ min ^−1^ reduction run of the synthesized α‐Fe_2_O_3_ powder with 11 % enrichment in ^57^Fe; spectra measured for 1 min at average temperatures of a) 503 °C and b) 584 °C.

Aside from α‐Fe_2_O_3_, Fe_3_O_4_, FeO and α‐Fe, an additional iron species was identified by Mössbauer spectroscopy. Its CS_475–544 °C_ was around 0.3 mm ⋅ s^−1^ (a value comparable to the CS value of iron in B‐sites of Fe_3_O_4_ and FeO) and the QS_475–544 °C_ was between 1.2–1.6 mm ⋅ s^−1^ which; however, did not fit to these oxides. Thus, the hyperfine interaction parameters (HIPs) could not be assigned to any of the bulk oxides (violet doublet in Figure 4a). The species appeared after the onset of the reduction in parallel with α‐Fe formation. Generally, such high QS values can be found e. g. in superparamagnetic iron species or hydroxides. Among the hydroxides, only the formation of Fe(OH)_2_ would make sense in the context of the reaction conditions.^[55]^ For Fe(OH)_2_ QS_RT_ values of around 3 mm s^−1^ have been reported,[^56^, ^57^] which is significantly larger than the observed value. For superparamagnetic species, Fe_3_O_4_ could be considered. Other superparamagnetic species can be ruled out due to experimental conditions and CS_475–544 °C_ values.[^58^, ^59^, ^60^] For superparamagnetic Fe_3_O_4_ QS_RT_ values around 1 mm s^−1^ are reported.[^61^, ^62^, ^63^] Since superparamagnetic Fe_3_O_4_ fits best the observed HIPs, we assigned this species to the given doublet.

The larger observed QS_475–544 °C_ of 1.2–1.6 mm s^−1^ could eventually be explained by interactions of the Fe_3_O_4_ structure with water. It is known[^56^, ^64^] that interaction with water can induce additional distortions in the crystal lattice, resulting in an increase in QS. This could indicate that water was trapped in the pores and/or the crystal structure during the reduction (see the DFT section for further discussion). When the temperature reached 544 °C, the signal assigned to superparamagnetic Fe_3_O_4_ vanished and the typical asymmetric FeO doublet was found, as shown in Figure 4b. This indicated that superparamagnetic Fe_3_O_4_ might have been an intermediate leading to FeO formation.

In order to compare the phase composition results from Mössbauer spectroscopy and XAS/XRD for the reduction processes, the XAS and Mössbauer results are shown as an overlay in Figure S9, displaying a very good agreement within the limits of each method. Similar results were obtained for the reduction with 15 K ⋅ min^−1^. Reduction was found to be finished at the same temperature within the margin of error. The intermediate oxides Fe_3_O_4_ and FeO were found to be formed subsequently, with a maximum in Fe_3_O_4_ content at a temperature around 530 °C. Only the onset of reduction was earlier in case of XAS and XRD. This might have been due to the low amounts of species, which were still in the range of the detection limit of Mössbauer spectroscopy, also considering the 1 min accumulation time for each spectrum in this experiment. Considering the rather large error bars of ±10 %_mol_ of the Mössbauer spectra, the determination of the starting point and transition points by this method alone would have been difficult.

To conclude, while time resolution was a weak point of MS compared to QEXAFS, it excels in its sensitivity for iron site identification. As such, the results from Mössbauer spectroscopy helped to identify the relevant iron speciation at temperatures below 570 °C during XAS measurements. Further, indications of a superparamagnetic Fe_3_O_4_ species with unique QS value might suggest that water transport out of the material was inhibited.

For heating with 2 K ⋅ min^−1^ (Figure S10), the reduction occurred in a similarly small temperature window for both XAS and Mössbauer but appeared to be shifted. In both cases, first an increase and then a decrease in Fe_3_O_4_ content was found, with maxima at 427 °C and 469 °C, spanning a temperature window of about 100 °C.

### DFT‐Simulations: Hydrogen Adsorption on the Hematite (α‐Fe_2_O_3_) (0001) Surface and its Reduction

**Figure 5:**
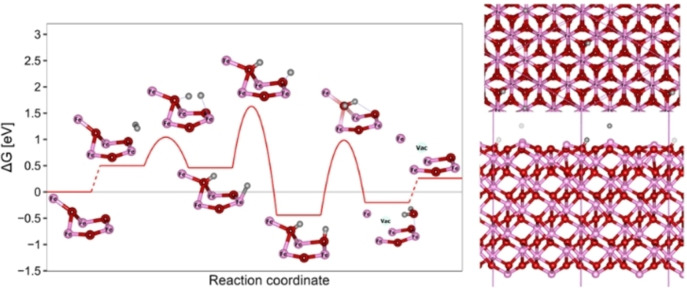
Reaction free energy diagram of the reduction of the (0001) orientation of the hematite surface at 400 °C and a hydrogen pressure of 1 atm. Hydrogen bonds are depicted with dashed lines. Top right – top view and bottom right – side view on simulation cell, second transition state is shown.

The primary phase in the hematite reduction process is the adsorption of hydrogen onto its surface. At this stage, it is critical to assess whether this step could in any way impede the oxide reduction. Using Density functional theory (DFT), energetics of hydrogen adsorption on the hematite surface and the subsequent water molecule elimination were calculated, the corresponding free energy diagram at 400 °C is shown in Figure 5, (see Figure S18 for free energy diagrams at other temperatures). Initially, a hydrogen molecule is weakly bonded to an iron atom. The splitting of the hydrogen molecule is facilitated by two oxygen atoms with a barrier of about 0.54 eV at 400 °C. The subsequent migration of a hydrogen atom from a surface iron to oxygen, with a barrier of 1.14 eV at 400 °C, corresponds to the global minimum of the studied system where now both hydrogen atoms are bound to nearby oxygen atoms, which is −0.44 eV below the initial state. Water elimination requires the migration of a hydrogen atom to the hydroxyl group, with a barrier of 0.98 eV. Note, that this barrier is at the low coverage limit (1/6 ML of hydroxyl). Water desorption is slightly uphill at 400 °C and becomes thermoneutral at 1090 °C (see SI).

We also investigated the feasibility of removing water molecules from the bulk phase of hematite using hydrogen as the reductive agent. Given the structure‘s relatively large interstitial size, several possible absorption pathways were explored, presented in Table 2. It turns out that the presence of a hydrogen molecule within an interstitial site is thermodynamically highly unfavorable. Oxygen vacancy formation within the structure is quite plausible, with a free energy of +0.55 eV at 400 °C. The bulk oxygen vacancy is thus about 0.29 eV less stable than the corresponding surface vacancy depicted in Figure 5. As hypothesized, the presence of a water molecule inside the pore is highly unfavorable. It was also established that a water molecule could easily occupy the oxygen vacancy site, hindering the reduction process in the bulk phase. It is conceivable that at relatively high temperatures, cracks and channels may form, enabling migration of water and hydrogen molecules, potentially accelerating the process significantly.

**Table 2:** Absorption of hydrogen and water in a hematite interstitial site (IS) at 400 °C.

State	ΔG [eV]
Fe_2_O_3_ (bulk)	0
H_2_ (molecule) in IS	2.76
2 H (dissociated and attached) in IS	1.90
O‐vacancy formation	0.55
H_2_O (molecule) in IS	5.34
H_2_O (in O‐vac of Fe_2_O_3_ lattice)	2.33

### Kinetic Modelling of the Hematite Reduction

Next a particle model was developed to simulate the reduction of α‐Fe_2_O_3_ microparticles. Details of this model are presented in the experimental section. Both the experiments and previous research indicated that the reduction occurred in three discrete steps with Fe_3_O_4_ and FeO as reaction intermediates (Eq.1–Eq.4), as found by XAS/XRD/SMS and reported in literature.[^42^, ^43^, ^65^] It is assumed that the reduction follows a three‐step global reaction mechanism (Eq. 1–3:
(1)

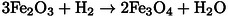



(2)

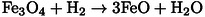



(3)

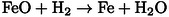



(4)

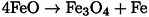




To consider the thermodynamic stability of FeO for temperatures above 570 °C, Eq. 4 is additionally taken into account.

**Figure 6:**
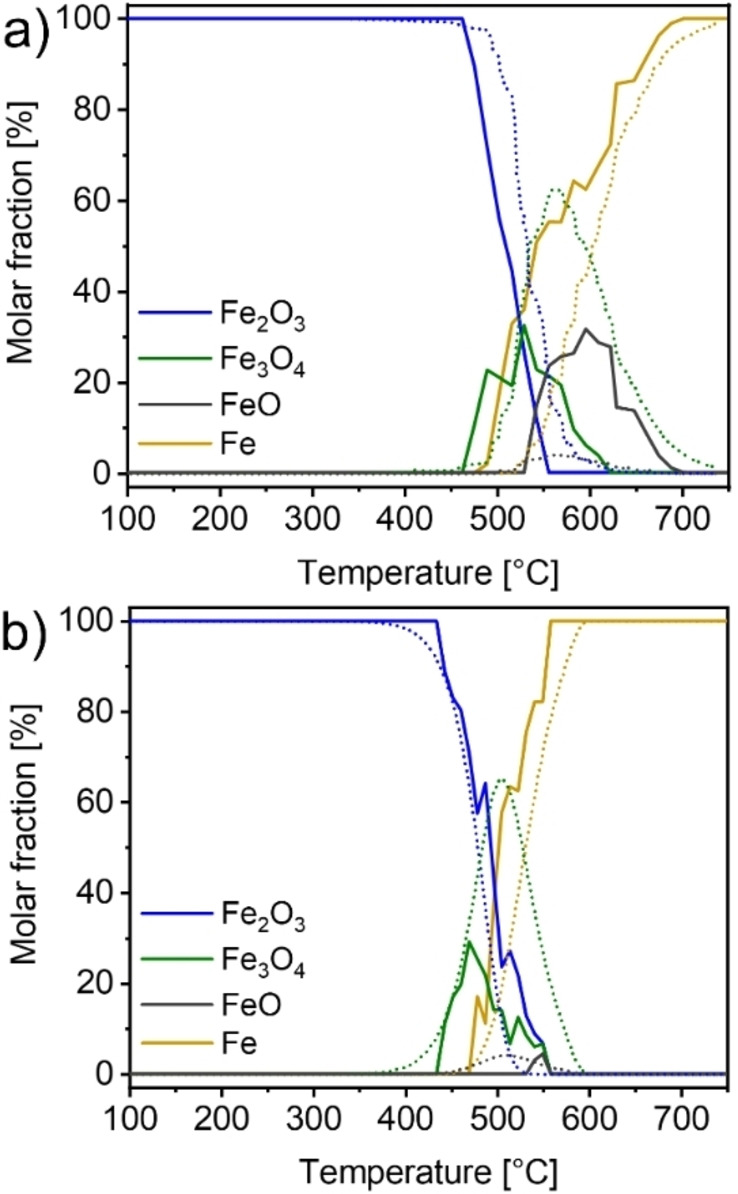
(a) Simulation of temperature‐programmed reduction with 15 K ⋅ min^−1^ heating rate, (b) Simulation of 2 K ⋅ min^−1^ reduction experiment in comparison to the original data from Mössbauer spectroscopy. The solid line represents the experimental data, the dotted line represents the results of the kinetic simulation.

Figure 6 presents a comparison between the simulation and the experimental results from Mössbauer spectroscopy. It should be noted that the kinetic model did not consider the super‐paramagnetic form of Fe_3_O_4_, as there was insufficient information available for this species. Therefore, its fraction was added to the fraction of bulk‐like Fe_3_O_4_. The kinetic parameters, E_a_ and A, for the three reaction equations were determined based on the experiments presented above. The identical set of kinetic parameters was applied to both experimental scenarios with different heating rates.

The comparison showed that the model effectively reproduces the onset and completion of the reduction process. The trends in α‐Fe_2_O_3_ consumption and metallic iron formation are in close agreement with the experimental data for both scenarios. However, in the kinetic model iron consumption started at slightly higher temperatures. Assuming a stepwise reaction mechanism, the reduction to iron could not occur prior to the formation of FeO. Furthermore, the simulation overestimated the formation of Fe_3_O_4_ for both heating rates, while it under‐estimated the formation of FeO at a heating rate of 15 K ⋅ min^−1^.

The experimental data suggest that the reduction of a particle followed a core‐shell mechanism. This implies a fast reduction of the particle surface, whereas the reduction within the particle was slower due to diffusion limitations.

**Figure 7:**
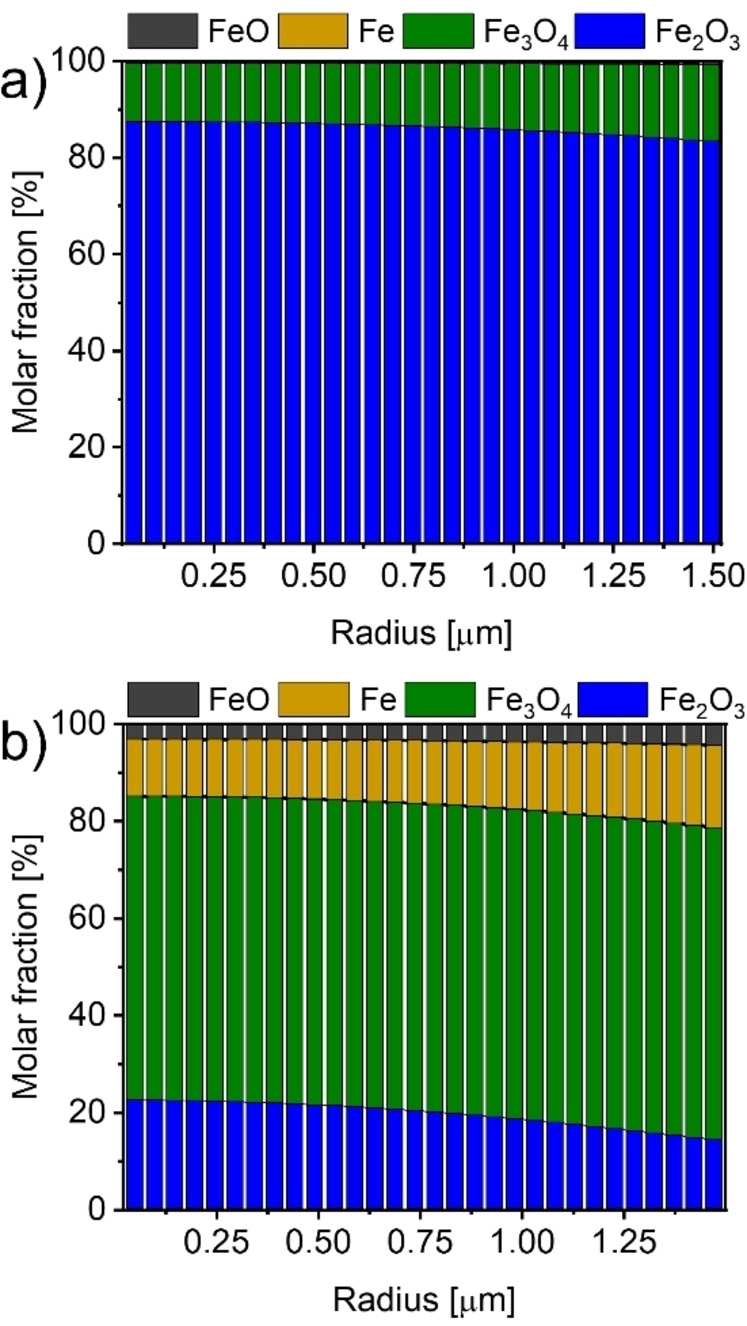
Simulated radial composition of a particle reduced at 500 °C with heating rates of (a) 2 K ⋅ min^−1^ and (b) 15 K ⋅ min^−1^.

However, accurate modeling of solid phase diffusion and the interaction between pore and solid phase diffusion presented significant challenges. Therefore, the particle model developed here simplified the scenario and considered a lump diffusion coefficient to cover all processes. Thus, this approach did not account for the formation of a dense iron layer. As a result, the model showed a homogeneous distribution of oxides over the entire particle radius rather than a distinct layer formation. However, with this initial approach, it was already evident that the degree of reduction was higher on the outer surface of the particle compared to the interior (Figure 7).

The observed deviations in FeO formation have been attributed to similar factors. At elevated temperatures, sintering became more pronounced, further limiting transport processes.[^66^, ^67^] This way, water produced during the reduction process could be trapped in the pores. This trapped water enhanced the stability of FeO, consequently further decelerating the reduction process.[^68^, ^69^, ^70^]

The experimental results indicated that the current model requires further refinement to accurately capture the underlying processes. Future refinement of the model should include descriptions of transport limitations and porosity changes due to reactions and sintering effects. Additional experimental data will be required to validate these changes, while theoretical calculations will be essential to determine the appropriate parameters.

## Conclusions

In this work we applied different synchrotron‐based techniques (XAS, XRD and SMS) to study *in situ* the changes during the reduction of hematite and correlated the results with data obtained by modeling and simulation. A single method was not able to describe the mechanism properly due to their individual limitations and therefore a study with complementary methods was used as a starting point.

XAS (in QEXAFS mode) offered a significantly better time resolution than most other techniques, with acquisition times of less than 100 ms possible, enabling a detailed analysis of the beginning and the end of the transformation. However, it is noted that it was difficult to accurately identify compositions in the middle of the reduction due to spectral dampening effects for the challenging inhomogeneous samples. This can be circumvented by XRD, which can easily be measured simultaneously and is not affected in a similar way. But neither of the two methods is able to measure magnetic effects to detect a species such as superparamagnetic Fe_3_O_4_.

Mössbauer spectroscopy, in contrast, is able to measure hyperfine interaction parameters and distinguish between bulk and small particles. Also, compositions can be determined. It is limited primarily by its lower time resolution that led to larger average errors compared to XAS and XRD. In consequence, it was more difficult to determine the starting point of the reactions by SMS.

We were able to show that iron oxide can be completely reduced to metallic iron regardless of the heating rate. The redox cycle can therefore continue with reoxidation without loss of efficiency due to unreduced oxidic species. Though, different species would be formed depending on the heating rate applied. We suggest that two different mechanisms could have occurred, depending on the reduction conditions, and that the differences most likely originate from the thermodynamic stability window of wüstite. The thermodynamic stability windows of the different phases could be extracted from the Baur Gläsner Diagram^[71]^ (Figure S19). The wüstite species is only stable above 570 °C[^72^, ^73^] and therefore becomes relevant only if the reduction process is complete at this point and some magnetite remains which can be converted to FeO.

**Scheme 1:**
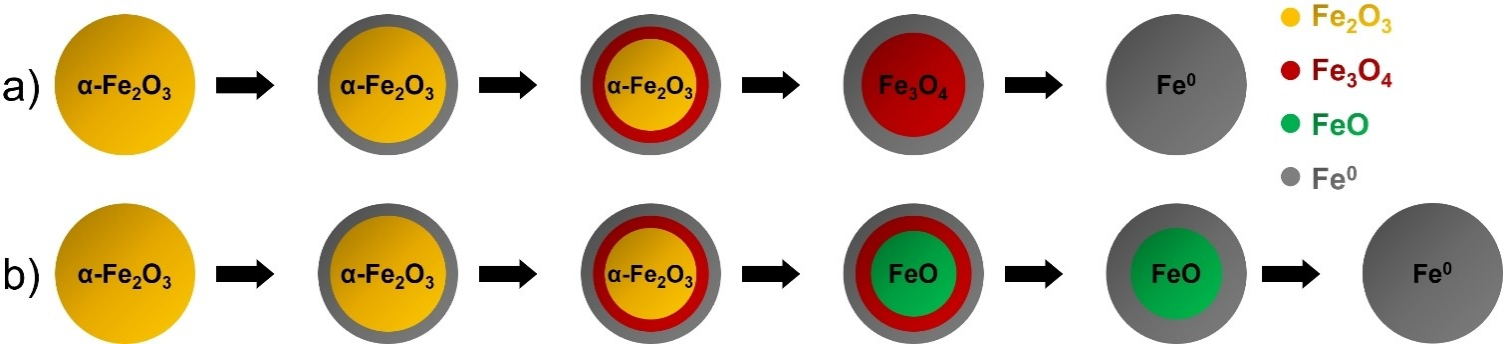
Schematic of the proposed mechanism with (a) three species involved (if the reduction is concluded below 570 °C), (b) four species involved including wüstite (in case of final reduction steps occurs above 570 °C).

For the slow heating rate, the reduction was completed at about 500 °C, a point at which wüstite had not yet reached thermo‐dynamic stability and thus did not form. An exemplary mechanism for this process is shown in Scheme 1, where after the formation of the metallic shell, a core of magnetite was formed, which gradually reduced over time.

In the case of the fast heating rate, the reduction of Fe_3_O_4_ was not completed below 620 °C. This means that the wüstite phase might have become thermodynamically stable and could be formed prior to full conversion to metallic iron, as displayed in Scheme 1. Water diffusion out of the particle was hindered due to the formation of the metallic iron shell. This shell trapped the water molecules inside the particles and accordingly stabilized the wüstite phase. DFT calculations showed a low favorability of hydrogen inside a pore, while the presence of water was highly favored by occupying oxygen vacancies. This also confirmed the observation by Mössbauer spectroscopy that water is trapped inside a porous Fe_3_O_4_ network at the beginning of the reduction after iron is formed.

The simulation represented the overall trends, especially starting and end points, quite well, but encountered difficulties in estimating the fractions of intermediate species. Our kinetic model so far only considered a lump diffusion coefficient for all processes, which might explain the deviations of intermediate species compared to experimental results.

The critical step of the reduction process for a future application according to Figure 1 and as demonstrated in this study is determined by the heating rate, but can be completed below 750 °C. Inorganic materials, such as iron and hydrogen, could work together to facilitate the transition to a carbon‐free circular energy economy. Renewable energy sources could be utilized to produce the green hydrogen, which can then be stored by reducing the (burned) iron oxide, thereby closing the cycle.

## Experimental Section

### Materials

Iron powder is obtained from Eckart TLS GmbH (99.8 % purity, Bitterfeld‐Wolfen, Germany). Isotopically enriched ^57^Fe metal powder (>95 % ^57^Fe) is purchased from CHEMGAS. Boron nitride powder (98 %, CAS−No: 10043–11–5, mean particle size of ~1 *μ*m), 35 wt % H_2_O_2_ solution (stabilized, for synthesis, CAS−No: 7722–84–1), 37 wt % HCl, Ethylene Glycol (98 %) and PVP (M_W_=55.000 g mol^−1^, CAS−No: 9003–39–8) are obtained from Sigma‐Aldrich. NaBH_4_ (99 %, CAS−No: 16940–66–2) is obtained from Arcos Organics.

Ethylene glycol was distilled under reduced pressure. All other chemicals were used without further purification.

Capillaries (Markröhrchen, 1 mm diameter, 0.02 mm wall thickness for XAS/XRD and 1.5 mm diameter, 0.01 mm wall thickness for SMS) are purchased from Hilgenberg GmbH, Germany.

### Preparation of Fe_2_O_3_ Particles with Small Diameter

The synthesis of iron oxide particles was adapted from Long *et al*.^[74]^ using partially enriched FeCl_3_ ⋅ 6 H_2_O.

^57^FeCl_3_ ⋅ 6H_2_O synthesis: 4.7 mg ^57^Fe and 42.2 mg Fe powder (total: 0.84 mmol, 1 eq.) were placed into a 50 mL Schlenk flask with a magnetic stirring rod and connected to a reflux cooler. The Schlenk flask was evacuated and flushed with N_2_ three times. 15 mL Millipore water and 0.735 mL conc. HCl (8.4 mmol, 10 eq.) were added, and the mixture was stirred at 60 °C for 1 h until bubble formation ceased and a clear solution was obtained. 0.77 mL 35 wt % H_2_O_2_ (8.4 mmol, 10 eq.) was added slowly and resulted in a bright yellow solution. After stirring for 30 min, the temperature was raised to 120 °C, and all liquid was removed at reduced pressure to yield 225.5 mg (0.834 mmol, 99 %) of a dark brown solid.

α‐^57^Fe_2_O_3_ particle synthesis: 255.5 mg of the partially enriched ^57^FeCl_3_ ⋅ 6H_2_O prepared in the previous step was dissolved in 13.44 mL of freshly distilled Et(OH)_2_ to give a 0.0625 M solution. 1.120 g PVP was dissolved in 26.87 mL freshly distilled Et(OH)_2_ to give a 0.375 M solution. In a 250 mL three‐neck flask connected to a reflux cooler, 26.87 mL Et(OH)_2_ and 250.9 mg NaBH_4_ (6.63 mmol, 7.5 eq.) were added. The solution was stirred at 1200 rpm while adding 200 μL PVP solution followed by 100 μL FeCl_3_‐solution in turn, until both solutions were fully added. The resulting solution was refluxed at 230 °C for 30 minutes, resulting in precipitation of solids. The mixture was then cooled down, the precipitate separated from the solution by centrifugation, and the resulting solid was washed three times with 15 mL acetone and dried in a drying oven. 90.5 mg of yellow powder was obtained. The powder was calcinated (700 °C, 1 h, air) to give 42.5 mg (0.266 mmol, 60 % with respect to Fe) of an orange‐brown powder. Mössbauer spectroscopy and XAS both revealed the powder to be pure α‐Fe_2_O_3_.

### Characterization and Modelling

The *in situ* quick‐scanning X‐ray absorption spectra (QEXAFS) spectra were recorded at the P64 beamline of the PETRA III synchrotron radiation source (DESY, Hamburg, Germany).^[75]^ Spectra were recorded in transmission mode at the Fe K absorption edge in the energy range of 6990 to 7761 eV. Higher harmonics were rejected by a pair of Si plane mirrors installed in front of the monochromator. The energy of the X‐ray photons was selected by an oscillating Si (111) channel‐cut monochromator (CCM) and the beam size was approx. 1 (vertical)×1 (horizontal) mm^2^. CCM oscillation frequency was set to 1 Hz.^[76]^ All measured X‐ray absorption spectra were first averaged to obtain 150 spectra per any single experiment using JAQ software 3.3.49v5.3 by Oliver Müller (BU Wuppertal).^[77]^ Energy alignment by using the spectra of Fe foil, which were recorded simultaneously with the sample spectra, and rebinning of the spectra were done in Fastosh 1.05.^[76]^ The rebinned spectra were then imported and normalized using the Athena 0.8.056 program from the IFEFFIT software package.^[76]^ Linear combination analysis (LCA) was performed in Athena on the normalized spectra in the range of 7110 and 7170 eV to obtain the bulk phase concentration of the different iron oxides. For this, Fe_3_O_4_ and FeO references were measured and used as they were, while the first and very last spectra of the corresponding reduction datasets were used as reference spectra were used as reference for α‐Fe_2_O_3_ and Fe^0^, respectively. The difference between the first/last *in situ* spectra and the corresponding α‐Fe_2_O_3_ and Fe^0^ reference spectra is only in slight dampening of spectral features (within 10 % of the maximum intensity) while relative intensities and peak positions stay the same as in the reference spectra. The dampening was caused by high dilution of rather large iron oxide particles, which led to an inhomogeneous sample density in the beam, and not due to chemical effects. At the same time, due to low fraction of Fe_3_O_4_ and FeO species in the spectral datasets the misfit which arises from using reference spectra without spectral dampening is negligible. Attempts were made to include other reference spectra such as γ‐Fe_2_O_3_ in the LCA, however their refined fractions were always close to zero.

The measurements were conducted in a plug flow quartz reactor with fixed heating rate. The iron oxide powder was diluted 1 : 10 with boron nitride (sample bed length 2–3 mm) and placed in 1.0‐mm‐diameter quartz capillaries (0.02 mm wall thickness, Markröhrchen, Hilgenberg GmbH, sample bed length 3 mm). A flow rate of 50 ml ⋅ min^−1^ (5 vol . % H_2_ in Helium) was set for all the experiments. The gases were dosed using mass flow controllers (El‐Flow Select, Bronkhorst) and monitored with a mass spectrometer (OmniStar GSD‐320 O1, Pfeiffer Vacuum GmbH) to ensure the correct gas concentrations. Samples were heated by means of a hot air blower (Leister LE mini sensor kit 800 W) from 20 to 750 °C.

X‐Ray diffraction (XRD) analysis was performed simultaneously with QEXAFS using a Pilatus 100 K diffractometer from the DESY detector loan pool. Diffractograms were taken every 2 seconds at an energy of 7700 eV. Data calibration, image integration and evaluation were performed with the Dioptas software by C. Prescher.^[78]^ For the calibration the Pilatus 100 k was selected as the detector, the wavelength was set to 1.61020 Å and the polarization to 0.990. A synthetic image with lanthanum hexaboride (LaB_6_) and boron nitride (BN) reflections was used as a reference to increase the number of available rings for the calibration. References used for the experimental reflection assignment: α‐Fe_2_O_3_ (ICSD collection code 164010), Fe_3_O_4_ (ICSD code 183969), FeO (ICSD code 27237), α‐Fe (ICSD code 64998), γ‐Fe_2_O_3_ (ICSD code 87119), and BN (ICSD code 83729).

Synchrotron Mössbauer source (SMS) measurements were performed at the ID18 beamline of the European Synchrotron Research Facility (ESRF, Grenoble, France) in transmission mode with 14.4 keV to excite iron nuclear levels.^[79]^ The beam size was 10 μm horizontal×5 μm vertical and was aimed at the center of the 1.5 mm diameter capillary to ensure homogeneous sample loading along the path of the beam. Four inclined avalanche photodiode detectors are used. Sample consists of a 1 : 10 mixture (by weight) of synthesized α‐^57^Fe_2_O_3_ particles and boron nitride. Usual bed length was 5–6 mm and usual sample mass 4 ±1 mg. The same experimental setup, including gas blower and MFCs, was used as for XAS (QEXAFS) measurements. The distance from gas blower to sample was 5–6 mm. Gas flow was 50 mL ⋅ min^−1^ to achieve equal flow rate as in the QEXAFS experiments with the smaller capillary.

Spectra were collected every minute under *in situ* conditions, while spectra with higher statistics were collected 1) prior to reduction at RT already under H_2_/N_2_ flow (BoT condition), 2) after reaching the final temperature under H_2_/N_2_ flow (EoT condition), 3) after cooling down under H_2_/N_2_ flow to RT, to ensure purity of the initial and final compounds. Two experiments were performed with different heating rates, i. e. 2 and 15 K ⋅ min^−1^. The 2 K ⋅ min^−1^ experiment was performed once and 5 consecutive spectra were summed up, resulting in a temperature resolution of 10 K for data analysis. The 15 K ⋅ min^−1^ experiment was performed twice and the corresponding spectra collected over 1 min were summed up, resulting in a temperature resolution of 15 K. Data analysis was performed using the SyncMoss software package,^[80]^ which allows the instrumental function to be taken into account when fitting SMS data. The experimental function was determined and checked via the single line absorber K_2_Mg^57^Fe(CN)_6_, with nominal area density of the ^57^Fe isotope of 0.5 mg cm^−2^.^[81]^ Calibration was performed with a standard 25 μm α‐Fe foil.

SEM images of the synthesized iron oxide powder were taken on a ZEISS EVO 10 with SmartSEM V6.03 software package with acceleration voltage of 10 kV and 13 kV.

Physisorption measurements (BET) to determine the specific surface areas, as well as the pore volume, were performed using a Belsorp Mini II device from Bel Japan Inc. Around 200 mg of sample were used for each experiment. Before the measurement, the samples were degassed by heating to 200 °C for 2 h under vacuum. The data was analyzed using the Belsorp Adsorption/Desorption Data Analysis Software according to the principles of Brunauer‐Emmet‐Teller‐Isotherm (BET‐Isotherm) as well as Barrett‐Joyner‐Halenda‐theory (BJH) to determine the specific surface area and pore volume, respectively. Physisorption measurements of the synthesized iron oxide powder were performed on a Quadrasorb SI by Quantachrome Instruments with equally around 200 mg and outgas time of 10 h at 350 °C. Quantachrome QuadraWin software package was used for data analysis.

Ex situ X‐ray diffraction (XRD) was done with a D8 Advance diffractometer by the company Bruker. Measurements were done with Cu−K‐α‐radiation (Cu−K‐_α1_=1.5406 Å; Cu−K‐_α2_=1.5666 Å) between the 2 theta angles of 20 and 80° with an increment of 0.017°. The tube current was 40 mA and tube voltage 40 kV. Background removal was performed using the Diffrac.EVA software.

### Computational Details

We employed the Vienna Ab initio Simulation Package (VASP) for spin‐polarized first‐principles calculations.^[82]^ VASP is a density functional theory (DFT) code using pseudopotentials or the projector‐augmented wave method and a plane wave basis set.[^83^, ^84^] The calculation was run with the GGA‐PBE exchange‐correlation functional .^[85]^ In order to account for dispersion interactions, the DFT−D3 method of Grimme was used.^[86]^ To enhance the description of strongly correlated systems using ab initio methods, the Hubbard correction in Dudarev′s implementation was utilized.^[87]^ The value U=4 was chosen based on the analysis of results from previous theoretical works,[^88^, ^89^] selected in such a way as to substantially reproduce the physical and chemical properties of hematite. All calculations were performed using pseudopotentials with the following electronic configurations: Fe (3d^7^s^1^), O (2 s^2^2p^4^) and H (1 s^1^).

We set the kinetic energy cutoff to 450 eV. For the sampling of reciprocal space, we used the Monkhorst Pack 3×3×2 and 3×3×1 meshes for the 2×2×1 supercell of hematite consisting of 128 atoms and the slab of the same size respectively. The surface simulations were carried out with slabs separated by a 15 Å vacuum layer used to avoid interactions between periodic images. Calculations were performed on the (0001) surface with R−Fe termination, identified as the most stable configuration.^[90]^ To properly describe the magnetic properties of the system, magnetic moments were aligned along the [0001] direction in accordance with antiferromagnetic ordering.^[91]^ These parameters ensure convergence in the calculations of hydrogen adsorption energy on the hematite surface to 0.05 eV, which is sufficient for the purposes of the present study. The criterion for the electronic optimization was set at 10^−6^ eV and the ionic optimization criterion at 10^−2^ eV/Å^−1^. Gibbs energy for a series of potential adsorbates and intermediates was calculated in the harmonic approximation, and the transition state was sought using the Improved Dimer Method (IDM).^[92]^

### Kinetic Modelling

A particle model has been developed within the DETCHEM package^[93]^ for the kinetic analysis of the reduction of iron oxide microparticles. For the simulation spherical particles are assumed which can be segmented into a defined number of layers *k*. Each layer is modeled as a multi‐phase tank reactor with multiple phases and a fixed number of iron ions *N*_Fe*,k*_. Thus, the molar amounts of the solid phase fulfil (Eq. 5:

(5) $$N_{Fe,k}=\;n_{Fe,k}+n_{FeO,k}+2\;n_{Fe2O3,k}+3\;n_{Fe3O4,k}$$

Each layer has a composition‐dependent porosity $\epsilon_{k}.$ The volume $V_{k}$ of a layer is then computed by (Eq. 6:

(6) $$V_{k}=\frac{\sum_{i}\frac{n_{i,k}\;M_{i}}{\rho_{i}}\;}{1-\epsilon_{k}}$$

where $M_{i}\;$ denotes molar mass and $\rho_{i}$ density of the solid‐phase species. The outer radius $r_{i}$ (Eq. 7) of a layer is given by the total volume of a sphere:

(7) $$r_{k}=\sqrt[{3}]{\frac{4\pi }{3}\;\sum_{j=1}^{k}V_{j}}$$

For gas‐phase species a reaction‐diffusion equation (Eq. 8:

(8) $$\frac{d\;n_{i,k}}{dt}=\gamma_{k}\;R_{i,k}^{het}\;+\;4\pi \left(J_{i,k-1}r_{k-1}^{2}-J_{i,k}r_{k}^{2}\right)$$

is solved. Here $\gamma_{k}$ is the specific area per volume, $R_{i,k}^{het}$ is the heterogeneous reaction rate (with respect to contact area) and $J_{i,k}$ is the molar diffusive flux. If we assume cylindrical pores of given diameter $d_{p}$ , then $\gamma_{k}$ can be expressed as in Eq. 9:

(9) $$\gamma_{k}=\;\frac{4\;\epsilon_{k}}{d_{p}}$$

Finally, the diffusive fluxes are calculated using Fick′s law (Eq. 10:

(10) $$J_{i,k}=\;-D_{i}^{eff}\;{\left.\frac{\partial c_{i}}{\partial r}\right|}_{r=r_{k}}$$

where $c_{i}=n_{i,k}/\left(\epsilon_{k}V_{k}\right)$ is the gas‐phase concentration and $D_{i}^{eff}$ the effective diffusion coefficient.

Porosity of the particles is calculated based on the measured BET area (Table 1). An average initial particle diameter of 3 μm is assumed. The temperature dependence of the diffusion coefficient is modelled by an Arrhenius approach with E_a_=22 kJ mol^−1^ and D_0_=2.2 ⋅ 10^−10^ m^2^ s^−1^.

## Conflict of Interests

The authors declare no conflict of interest.

1

## Supporting information

As a service to our authors and readers, this journal provides supporting information supplied by the authors. Such materials are peer reviewed and may be re‐organized for online delivery, but are not copy‐edited or typeset. Technical support issues arising from supporting information (other than missing files) should be addressed to the authors.

Supporting Information

## Data Availability

The data that support the findings of this study are available from the corresponding author upon reasonable request.
