# Supplementary material for: Following the Structural Changes of Iron Oxides during Reduction under Transient Conditions
Source: ChemSusChem. 2024 Sep 3;17(24):e202401045. doi: 10.1002/cssc.202401045 (PMC11660749; doi:10.1002/cssc.202401045)
Supplement: Supplementary file 1 — Supporting Information [file CSSC-17-e202401045-s001.pdf]

ChemSusChem

Supporting Information

Following the Structural Changes of Iron Oxides during Reduction under Transient Conditions

Lukas Braun, Jonas Spielmann, Dmitry E. Doronkin, Carola Kuhn, Aleksandr Maliugin, Dmitry I. Sharapa, Isabel Huck, Jianing Bao, Steffen Tischer, Felix Studt, Olaf Deutschmann, Ulrike I. Kramm,* and Jan-Dierk Grunwaldt*

Table of Contents

1 Setups used at the synchrotrons S2

2 *Ex situ* characterization S3

3 *In situ XAS/XRD and Mössbauer results* S5

3.1 *In situ* Mössbauer spectra during 15 K·min^{-1} reduction run S10

3.2 *In situ* Mössbauer spectra during 2 K·min^{-1} reduction run S13

3.3 Hyperfine interaction parameters obtained during fitting of 15 K·min^{-1} reduction run by *in situ* Mössbauer spectroscopy. S16

3.4 Hyperfine interaction parameters obtained during fitting of 2 K·min^{-1} reduction run by *in situ* Mössbauer spectroscopy. S17

3.5 Comparison of Chemical shift and magnetic hyperfine field obtained at both heating ramps. S18

4 DFT and Baur-Gläsner diagram S19

5 Mössbauer parameter and composition calculation S20

6 DFT-Simulation: Hydrogen Adsorption on the Hematite (0001) Surface (Fe_2O_3) and its Reduction S32

1 Setups used at the synchrotrons

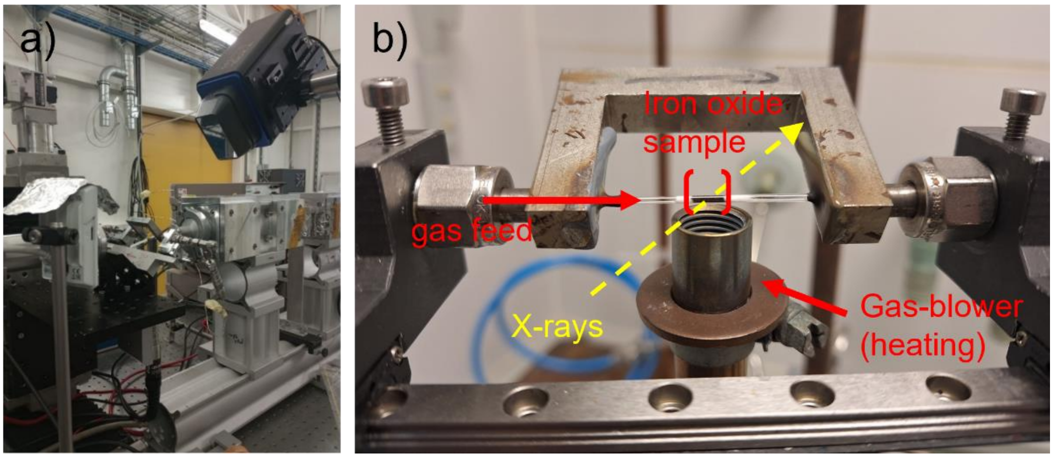

Setup S1 at P64, DESY for in situ QEXAFS and XRD measurements: a) Sample stage and detectors for simultaneous QEXAFS and XRD measurement, b) Sample and gas blower arrangement.

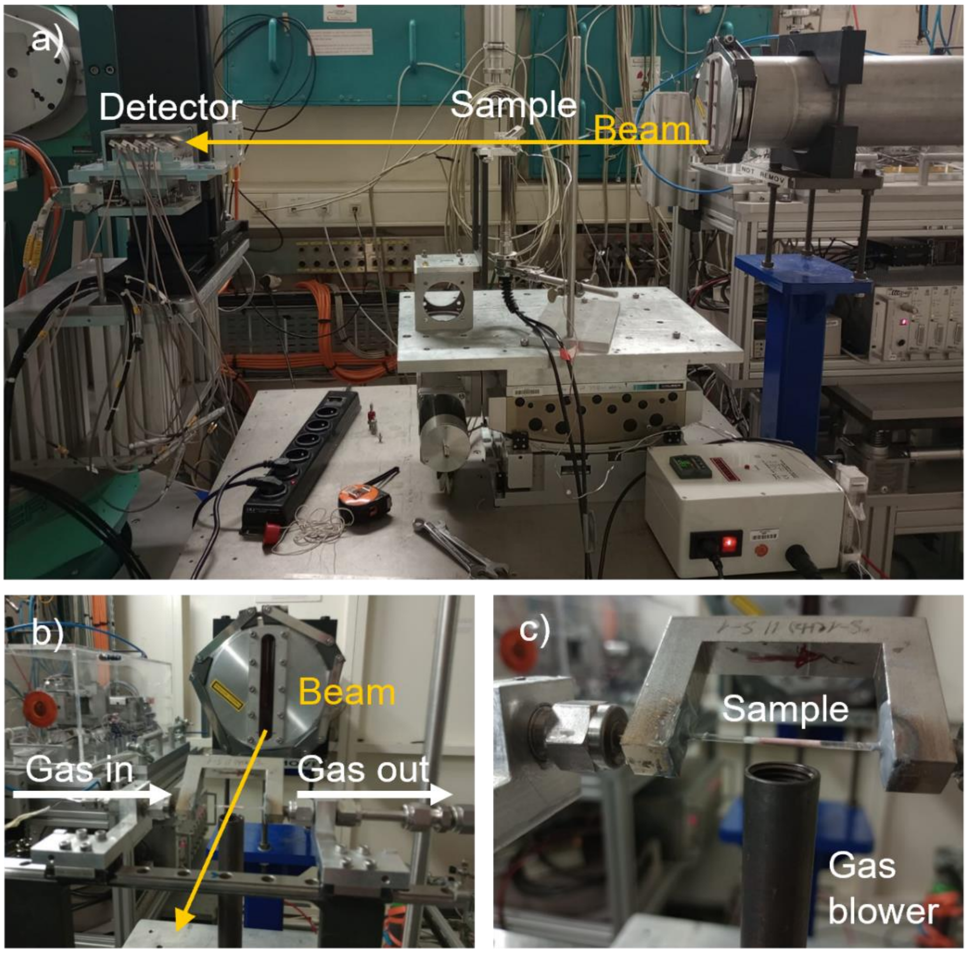

Setup S2 Setup at the ID18, ESRF for in situ Mössbauer measurements at a synchrotron source (a) Sample stage, beam source and detector, b) Gas connections, c) Sample and gas blower arrangement.

2 *Ex situ* characterization

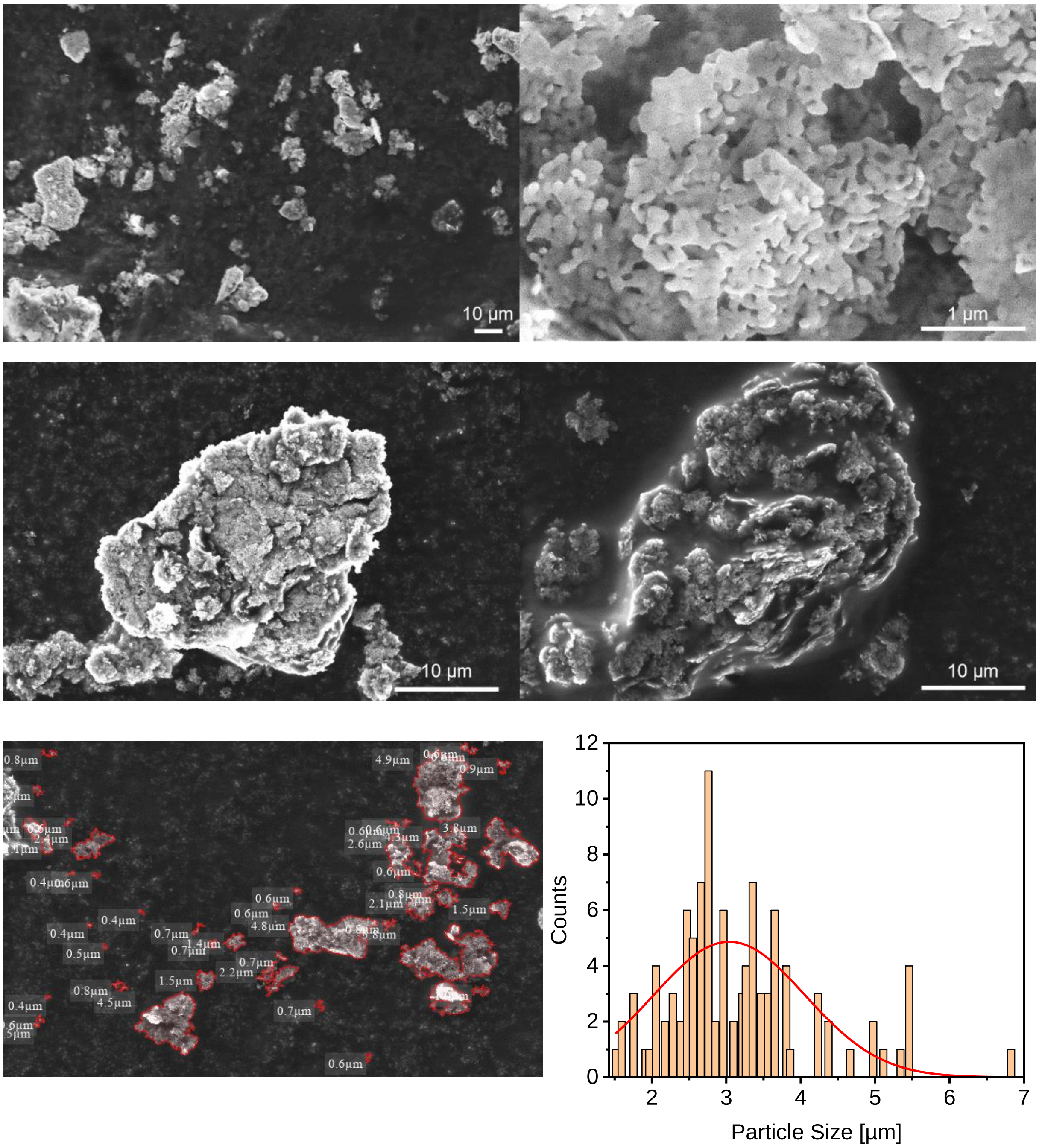

Figure S1. SEM figures of α-Fe_2O_3 with different magnifications.

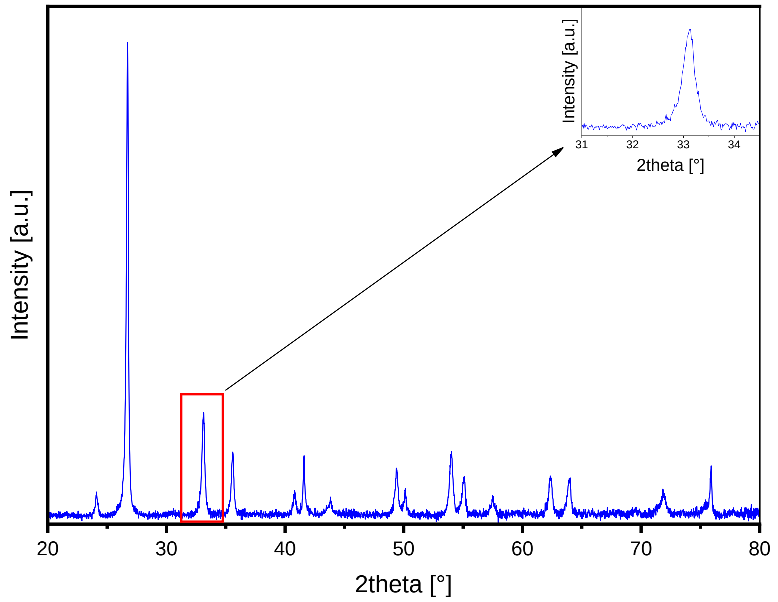

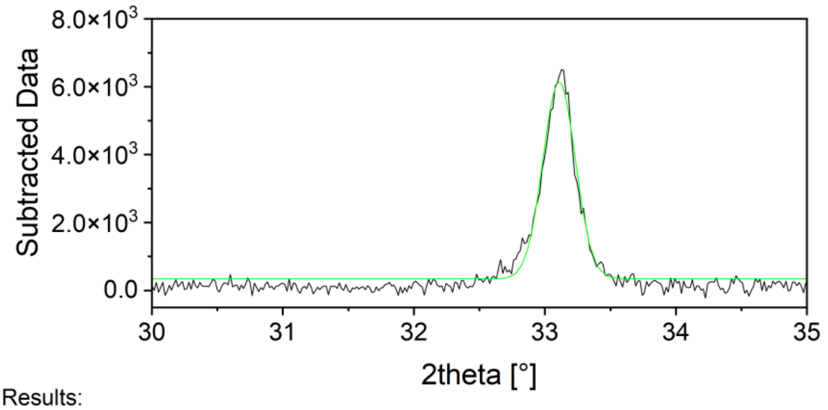

Fitting Results:

Peak Index	Peak Type	Area Intg	FWHM	Max Height	Center Grvty	Area IntgP
1	Gaussian	1232.22	0.29	5786.99	33.11	100.00

Figure S2. (a) Laboratory XRD pattern of α-Fe_2O_3 powder (10 wt%) in BN, b) Gauss fit of the (104)-reflex at 33.1° for calculation of the crystallite size via the Scherer equation (Cu-K-α1 = 1.5406 Å).

Particle size Calculations from BET

Estimated from the BET data by relating specific surface area to the reciprocal crystal size, considering the molar mass of Fe_2O_3, described in reference[1].

M(Al_2O_3) = 101,96 g/mol   density: 3,99 g/cm³

M(Fe_2O_3) = 159,69 g/mol density: 5,24 g/cm³

Ratio= 159,69/101,96 = 1,567

$S\_BET_{adjusted}$(Fe_2O_3) = 8,7 m^2/g *1,567 = 13,626 m^2/g

S_{BET}= A+D*b =-27,7834 +10686,91*b

D[$Å^{-1}$]= (S_{BET} -A)/b

D [$Å^{-1}$]= (13,626 +27,7834)/10686,91 =0,003875 $Å^{-1}$

Crystallise size = 1/D = 1/0,003875=258,08 Å = 25,8 nm

[1] S. Fogel, D. E. Doronkin, P. Gabrielsson, S. Dahl, *Appl. Catal. B Environ.* **2012**, *125*, 457–464.

3 *In situ XAS/XRD and Mössbauer results*

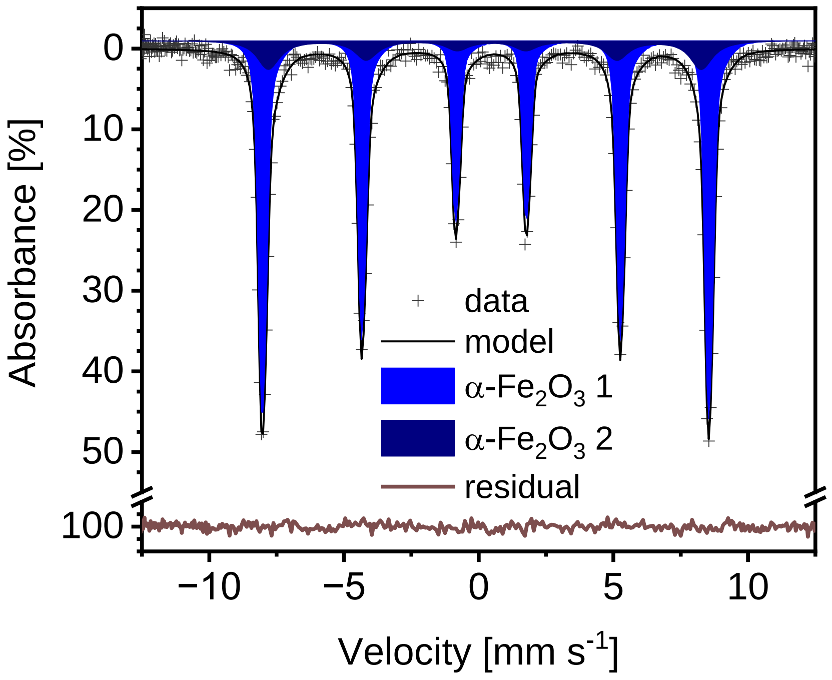

Figure S3. Mössbauer spectroscopy for phase identification of the α-Fe_2O_3 with particle size of 3 µm that was used for the *in situ* experiments.

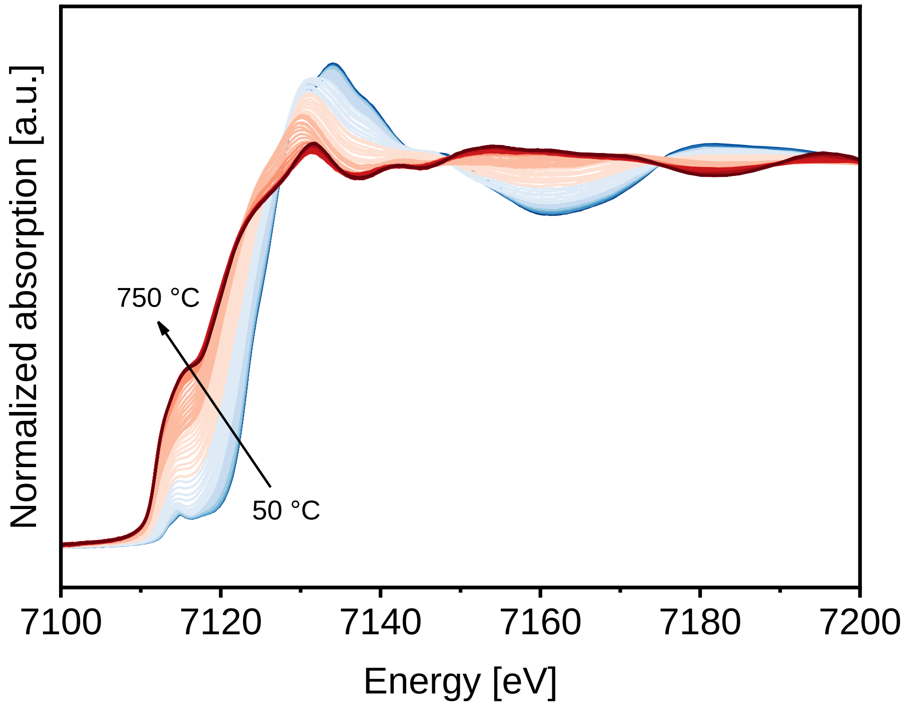

Figure S4. Normalized XANES spectra (Fe K-edge) of α-Fe_2O_3 3µm during TPR with a heating rate of 15 $K{\cdot}min^{-1}$.

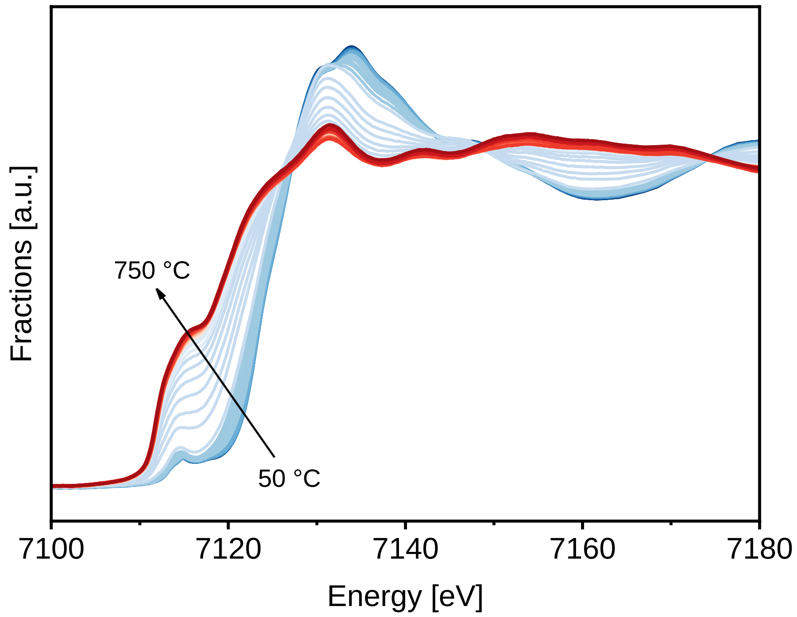

Figure S5. Normalized XANES spectra (Fe K-edge) of α-Fe_2O_3 3μm during TPR with a heating rate of 2 K·min^{-1}.

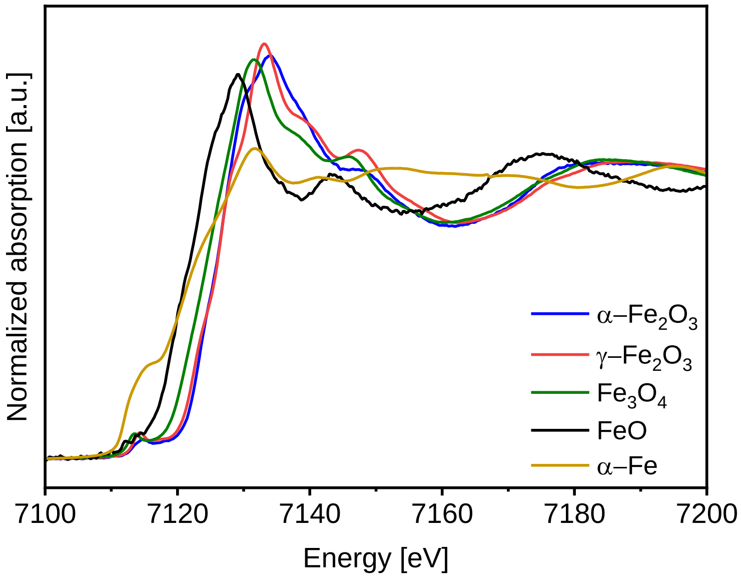

Figure S6. Normalized XANES spectra at the Fe K-edge of reference samples.

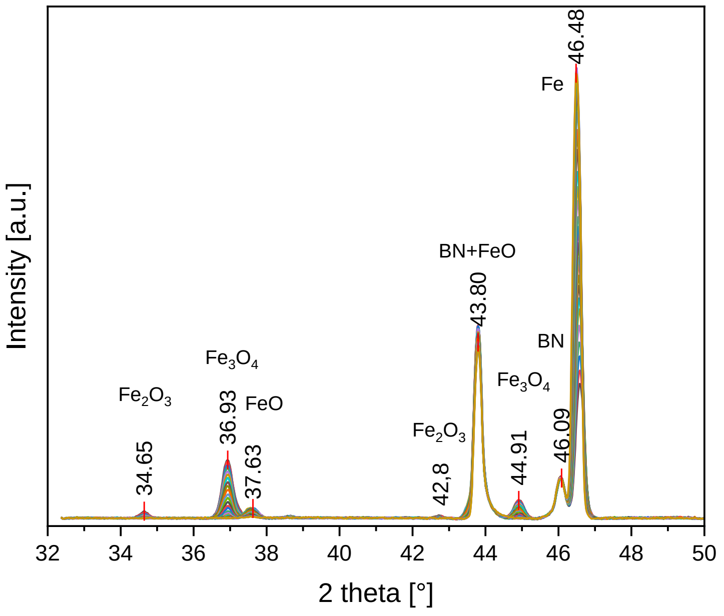

Figure S7. XRD reflections evolving as function of time during heating (15 K·min^{-1}) in 5% H_2 in He . Temperature from 545 °C to 677 °C.

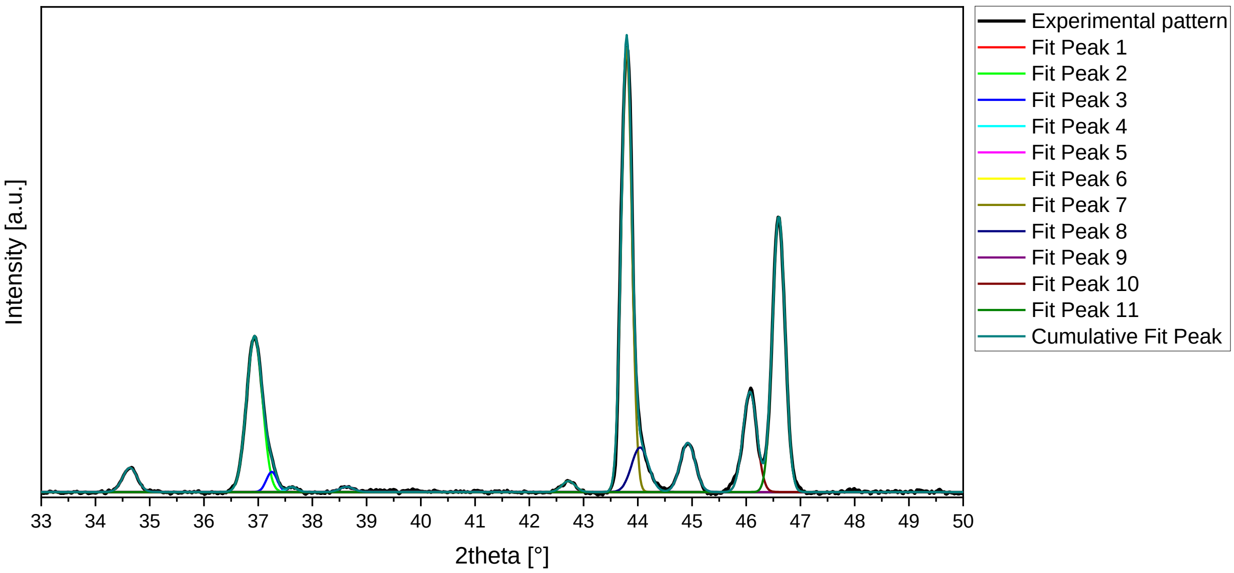

Figure S8. Multiple peak fitting results of the XRD spectra at 545 °C using a Gaussian model for fitting.

Peak1: 34.64° α-Fe_2O_3	Peak7: 43.79° BN
Peak2: 36.93° Fe_3O_4	Peak8: 44.04° FeO
Peak3: 37.25° α-Fe_2O_3	Peak9: 44.92° Fe_3O_4
Peak4: 37.62° FeO	Peak10: 46.07° BN
Peak5: 38.64° Fe_3O_4	Peak11: 46.60° Fe
Peak6: 42.72° α-Fe_2O_3	

Table S1: Assignment of XRD-signals from multiple peak fitting in Figure S8.

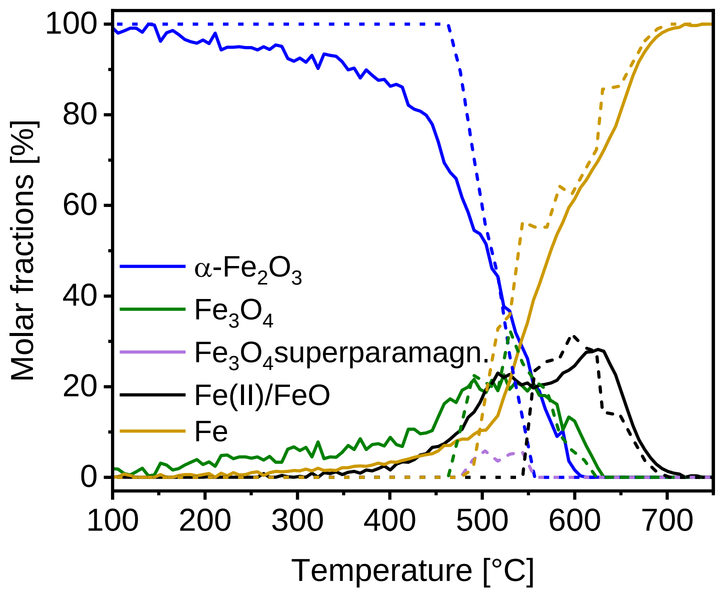

Figure S9. Comparison of the components according to XAS (solid line) and Mössbauer spectroscopy (dotted lines) during TPR with heating rate of 15 K·min^{-1}.

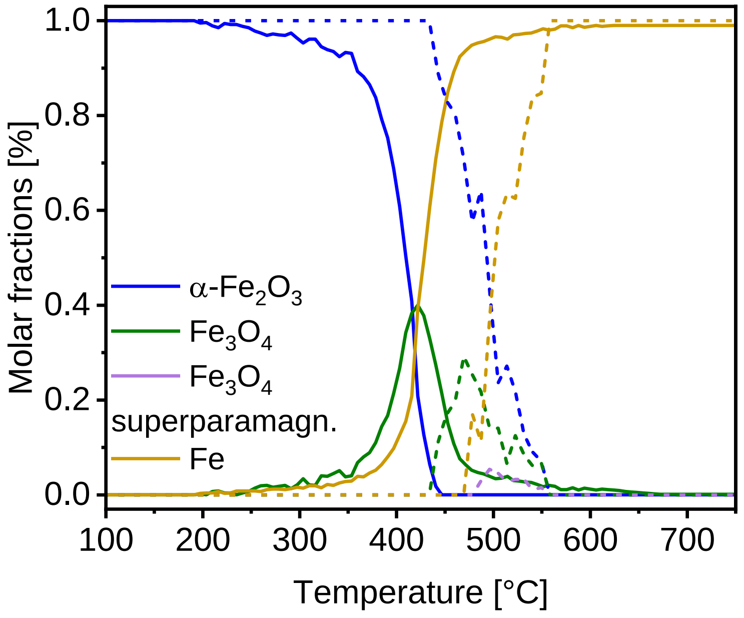

Figure S10. Comparison of XAS (solid line) and Mössbauer spectroscopy (dotted lines) for TPR with heating rate of 2 K·min^{-1}.

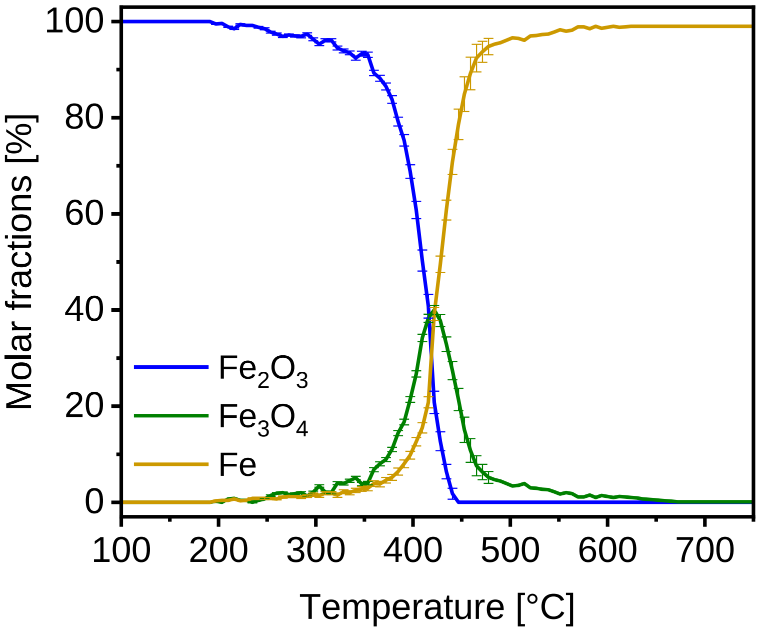

Figure S11. LCA of the XAS data during TPR with a heating rate of 2 K·min^{-1}.

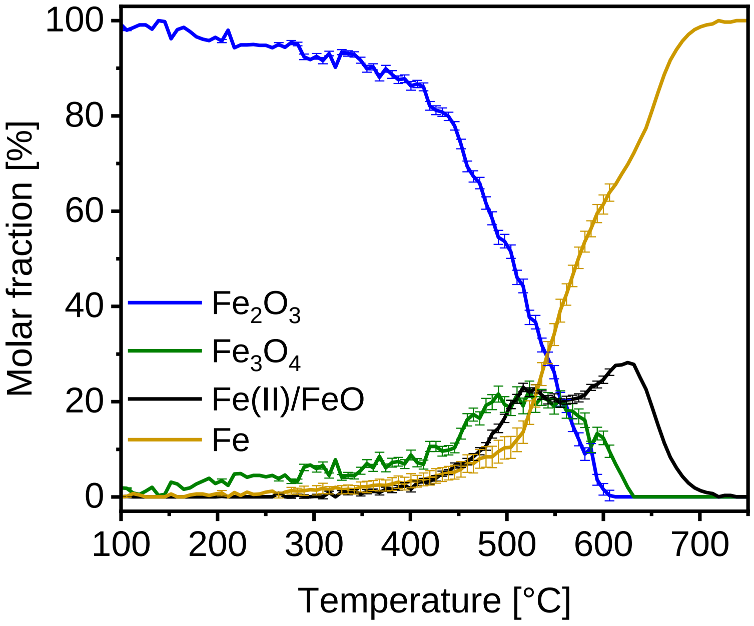

Figure S12. LCA of the XAS data for a TPR experiment with heating rate of 15 K·min^{-1}.

3.1 *In situ* Mössbauer spectra during 15 K·min^{-1} reduction run

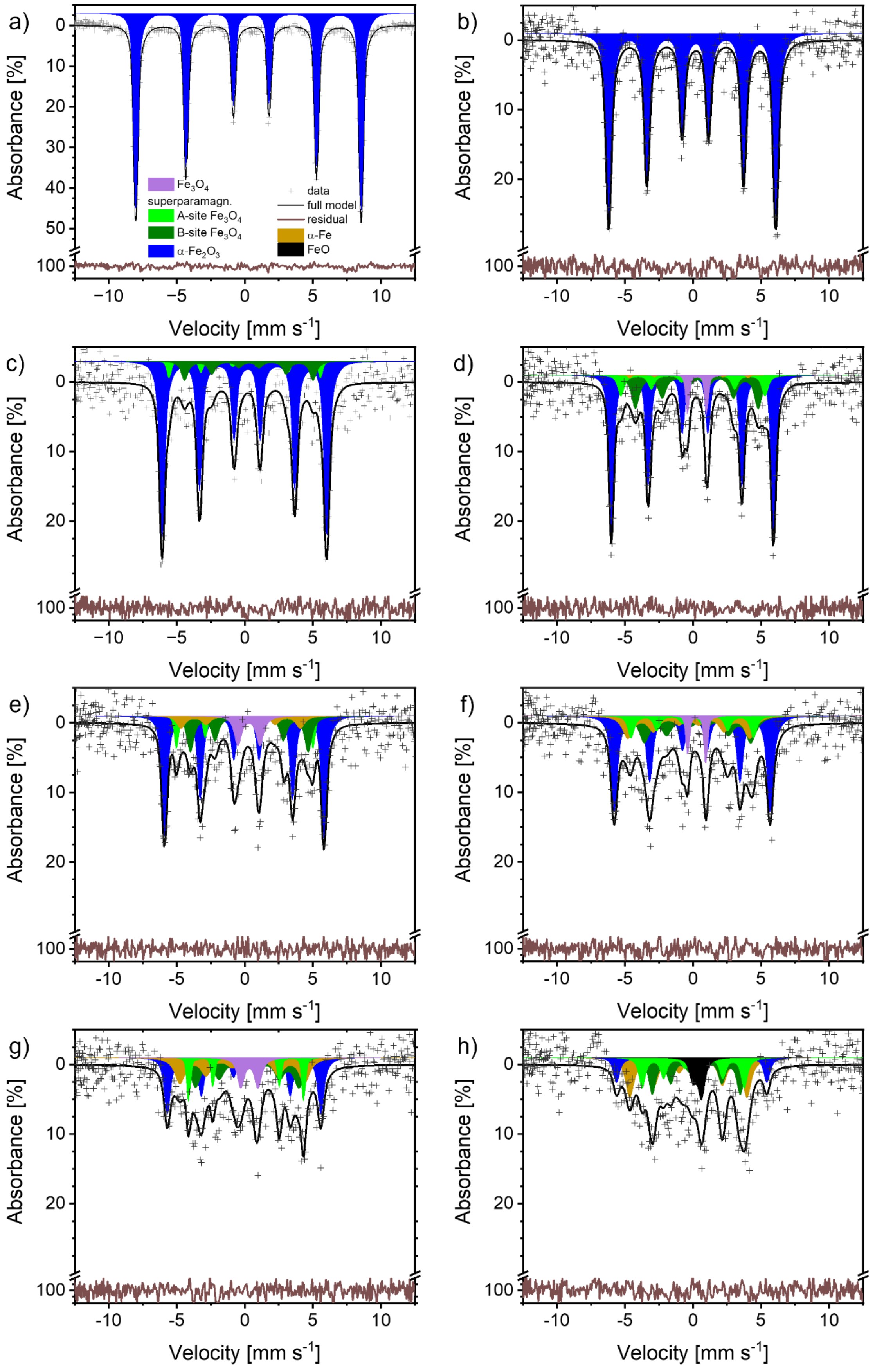

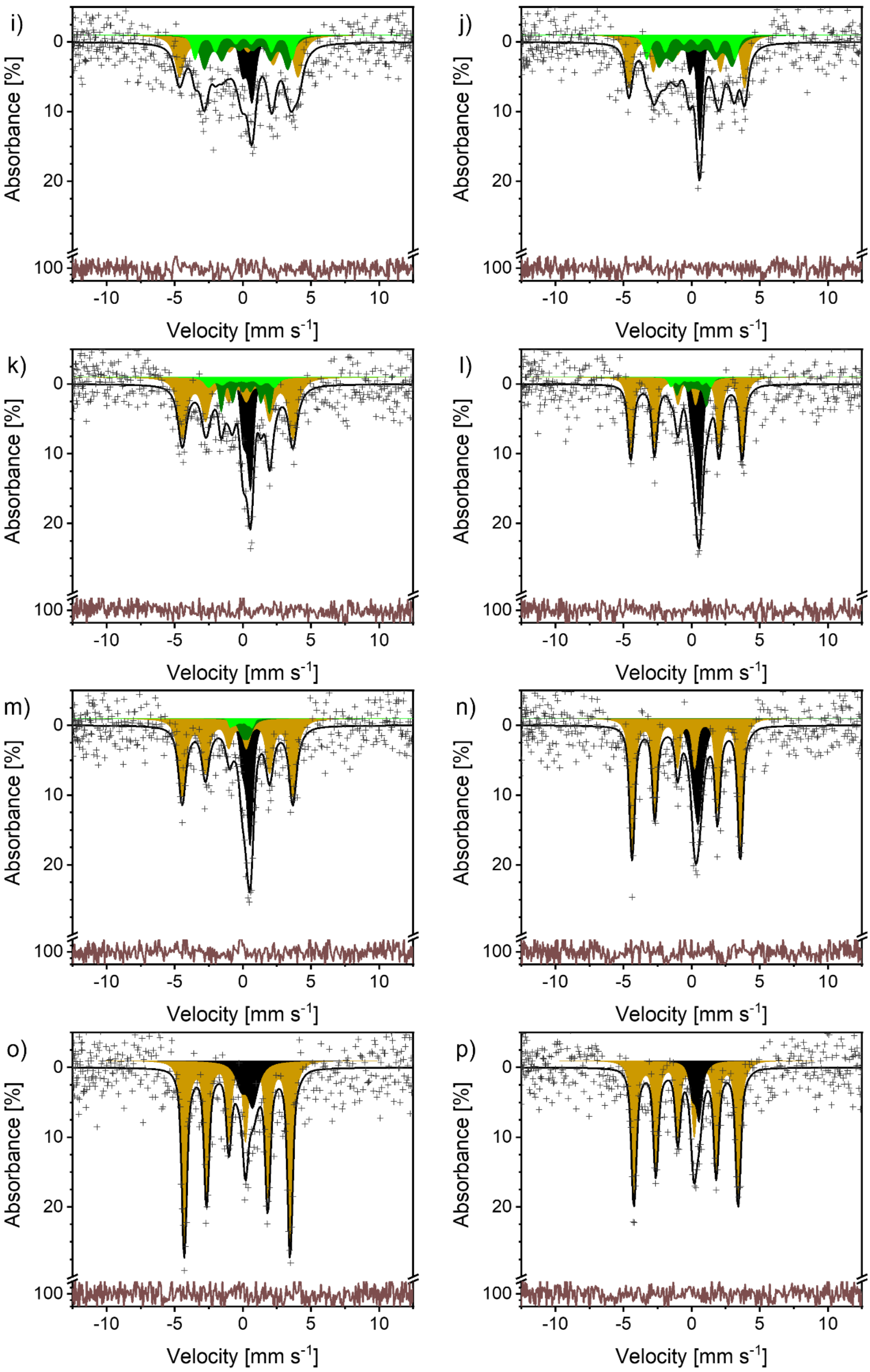

i)
j)
k)
l)
m)
n)
o)
p)
Absorbance [%]
0
10
20
100
-10
-5
0
5
10
Velocity [mm s-1]

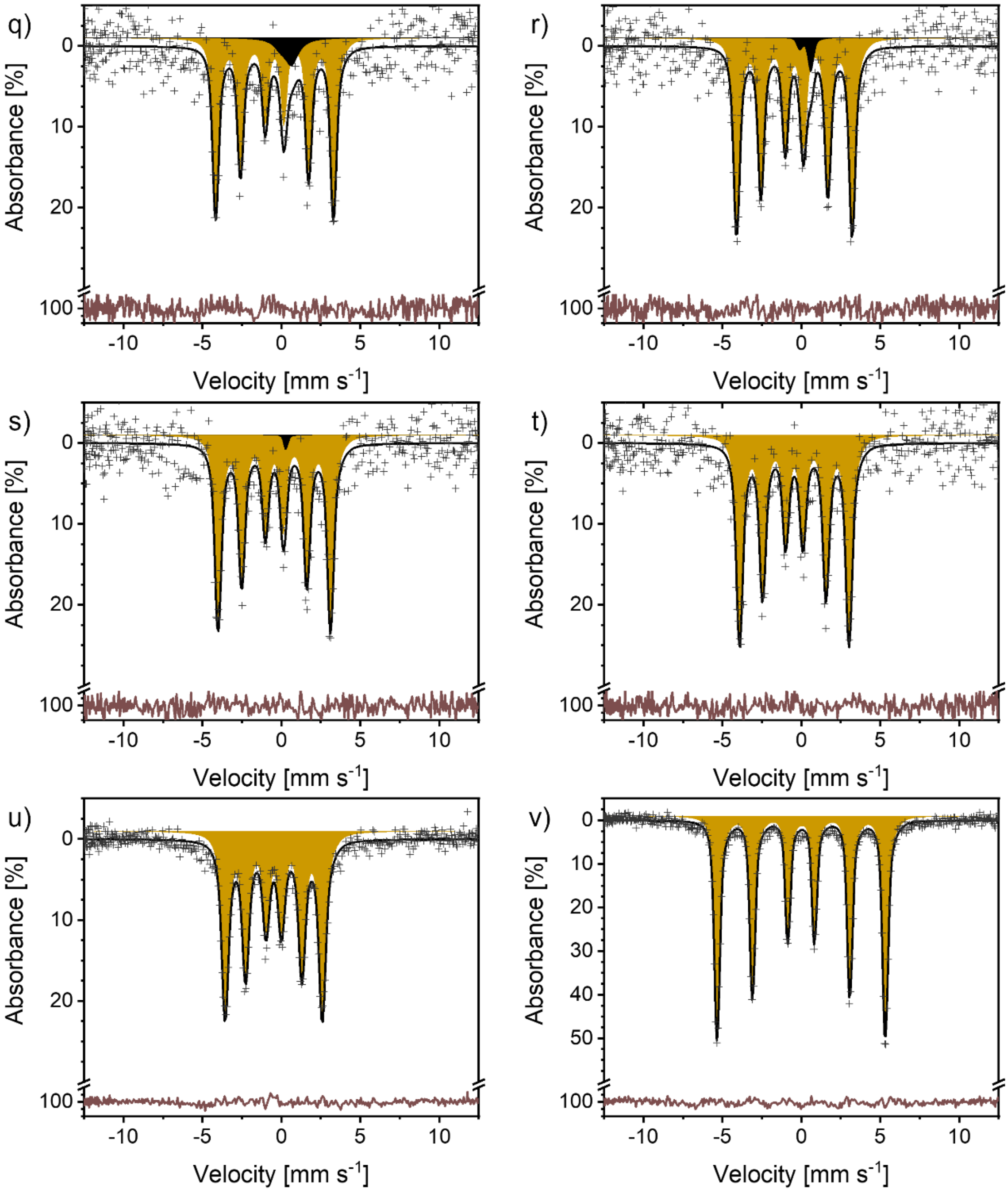

Figure S13: *In situ* Mössbauer spectra during a 15 K·min^{-1} reduction run of 3 µm sized α-Fe_2O_3 powder with 11 % enrichment in ^{57}Fe. a) Initial spectrum at RT with approx. 10.000 counts. b) – t) Spectra during ongoing reduction reaction acquired during two separate runs for 1 min and summed up at the following temperatures: b) 463 °C, c) 476 °C, d) 490 °C, e) 503 °C, f) 517 °C, g) 530 °C, h) 544 °C, i) 557 °C, j) 570 °C, k) 584 °C, l) 597 °C, m) 611 °C, n) 624 °C, o) 630 °C, p) 649 °C, q) 663 °C, r) 676 °C, s) 690 °C, t) 703 °C. u) measurement at final T = 754 °C for 30 min. v) Spectrum acquired at RT after cooling down.

3.2 In situ Mössbauer spectra during 2 K·min^{-1} reduction run

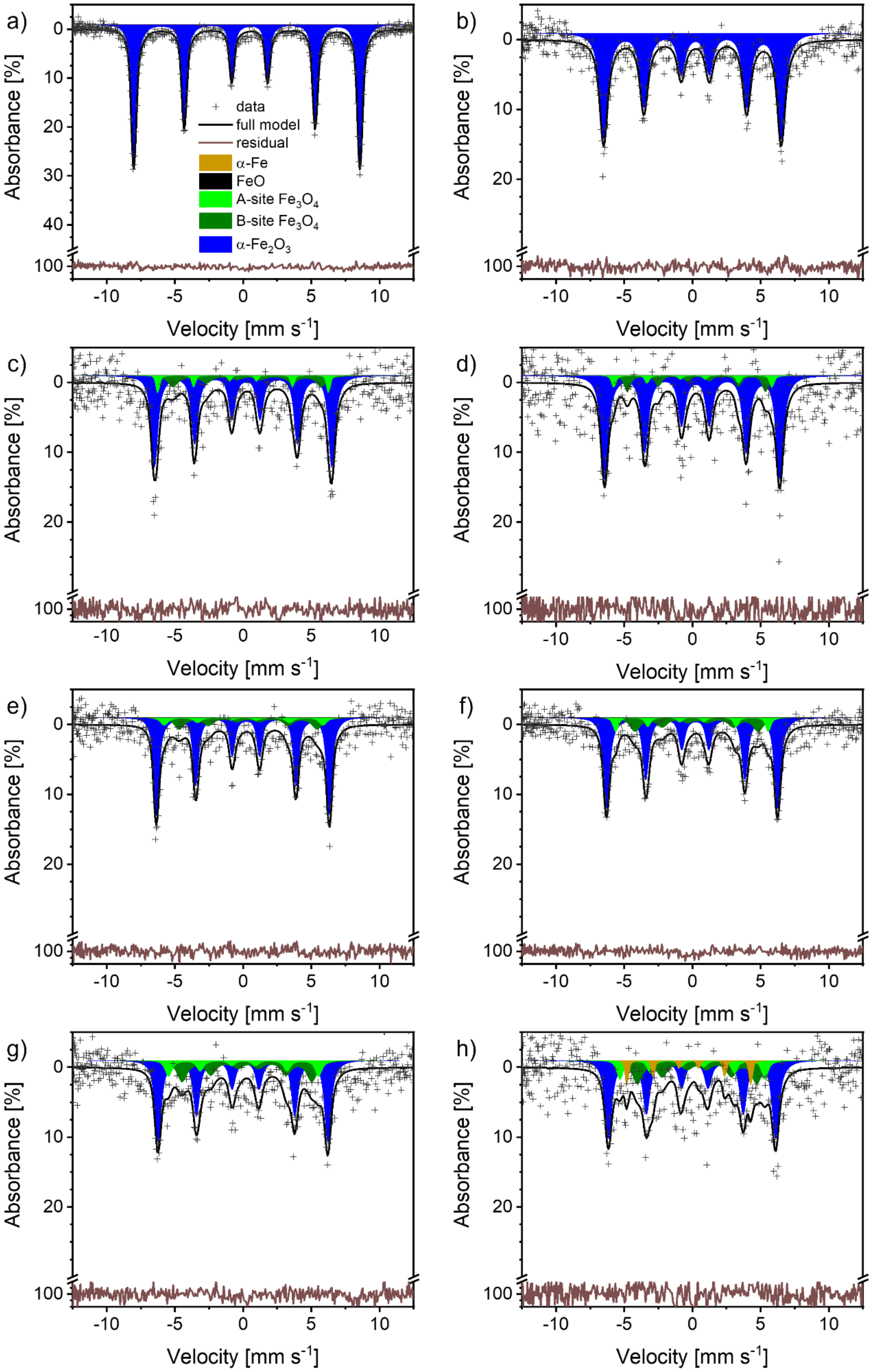

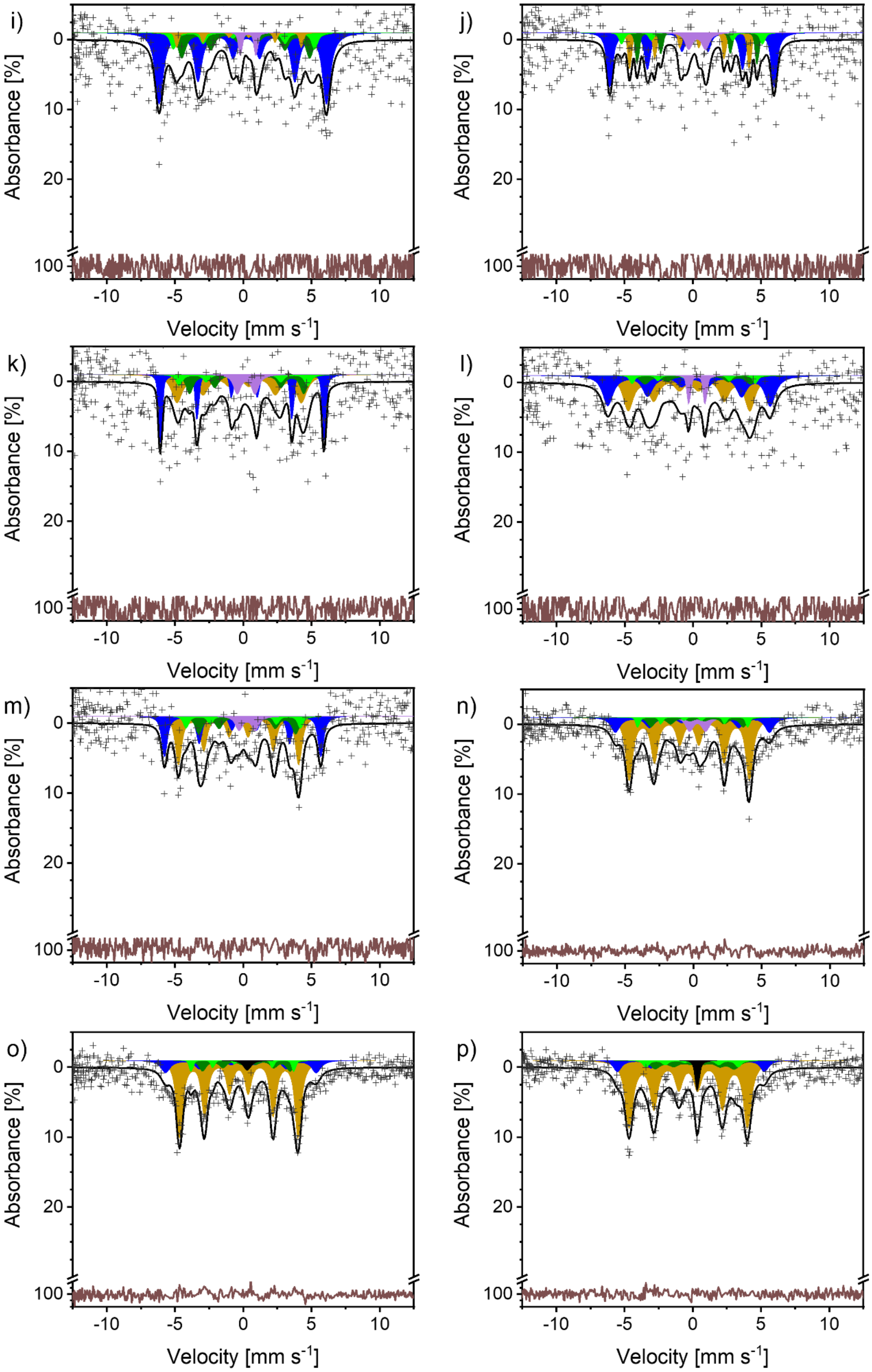
i)
j)
k)
l)
m)
n)
o)
p)
Absorbance [%]
0
10
20
100
-10
-5
0
5
10
Velocity [mm s-1]

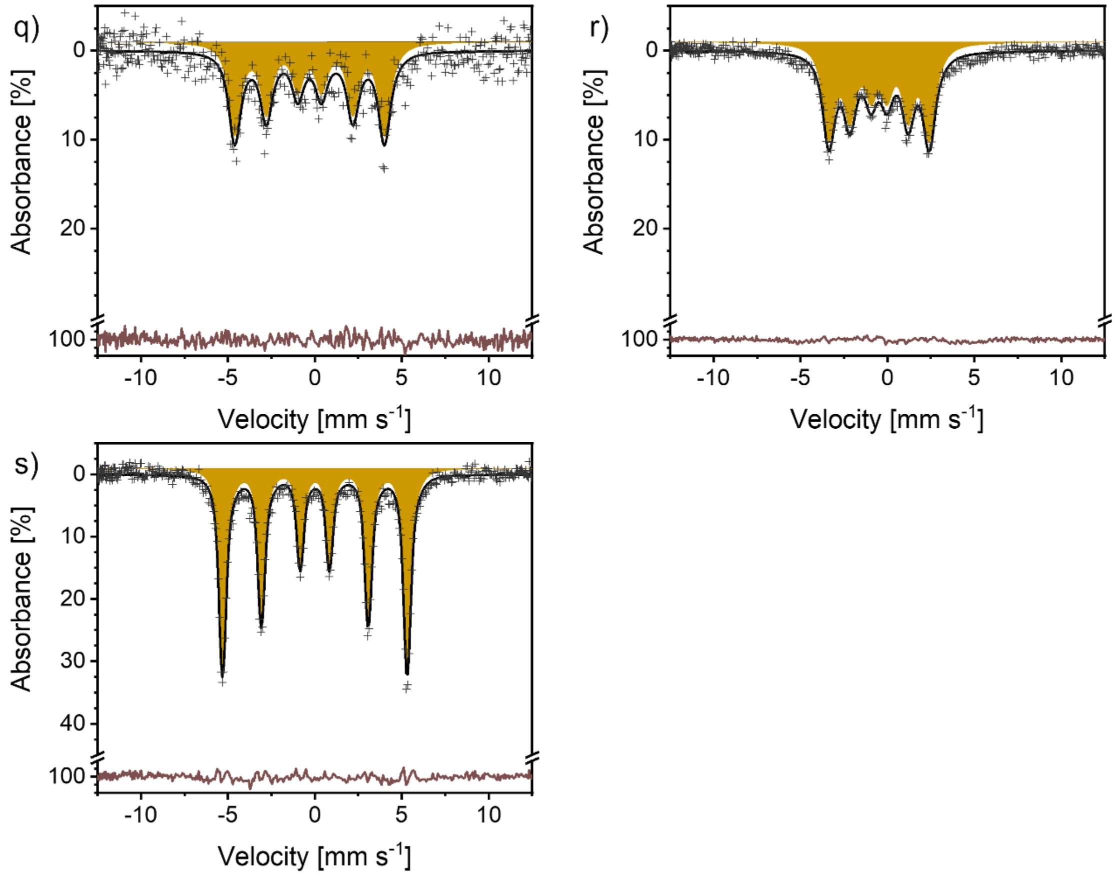

Figure S14: *In situ* Mössbauer spectra during a 2 K·min^{-1} reduction run of 3 µm sized α-Fe_2O_3 powder with 11 % enrichment in ^{57}Fe. a) Initial spectrum at RT with approx. 10.000 counts. b) – t) Spectra during ongoing reduction reaction acquired over 5 min at the following temperatures: b) 425 °C, c) 434 °C, d) 443 °C, e) 452 °C, f) 461 °C, g) 469 °C, h) 478 °C, i) 487 °C, j) 496 °C, k) 505 °C, l) 514 °C, m) 523 °C, n) 532 °C, o) 540 °C, p) 549 °C, q) 558 °C, r) measurement at final T = 754 °C for 30 min. s) Spectrum acquired at RT after cooling down.

3.3 Hyperfine interaction parameters obtained during fitting of 15 K·min^{-1} reduction run by *in situ* Mössbauer spectroscopy.

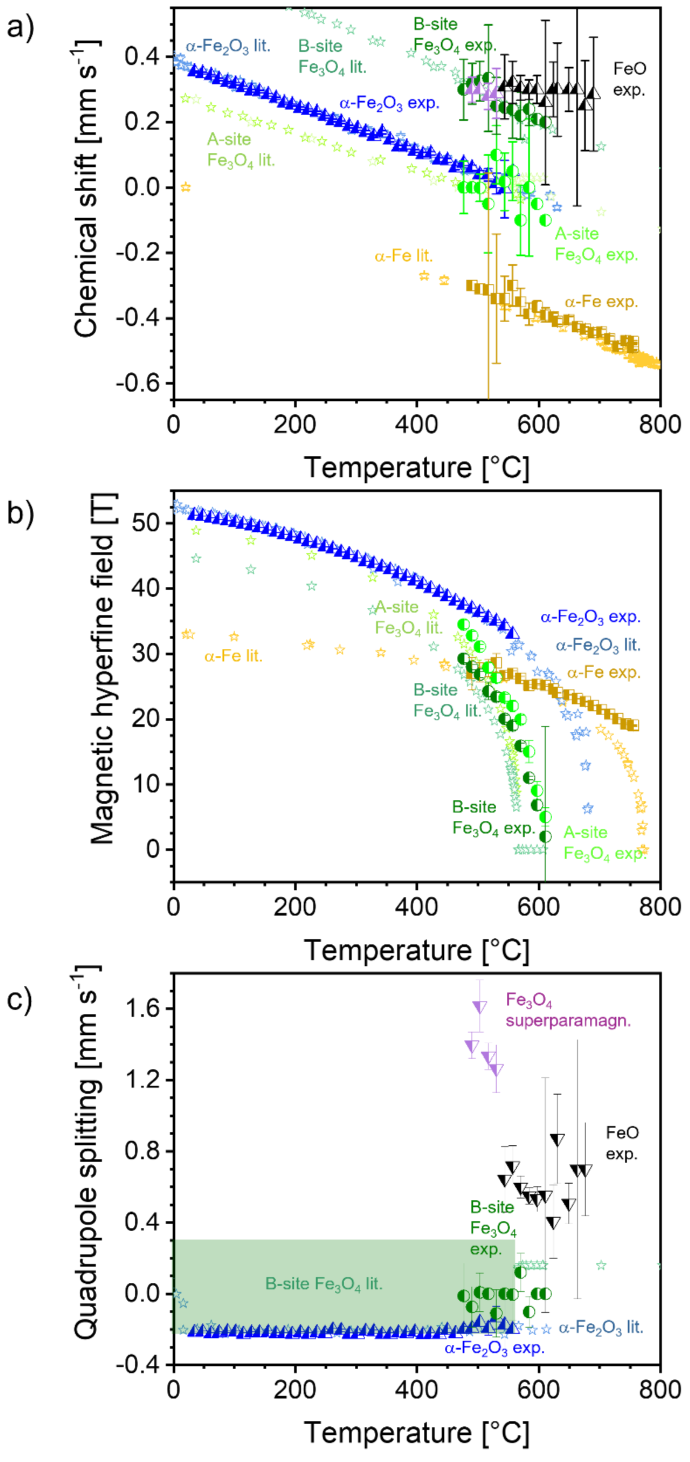

Figure S15: Hyperfine interaction parameters derived from Mössbauer spectroscopy of the reduction at a heating rate of 15 K·min^{-1}. a) chemical shift, b) magnetic hyperfine field and c) quadrupole splitting.

3.4 Hyperfine interaction parameters obtained during fitting of 2 K·min^{-1} reduction run by *in situ* Mössbauer spectroscopy.

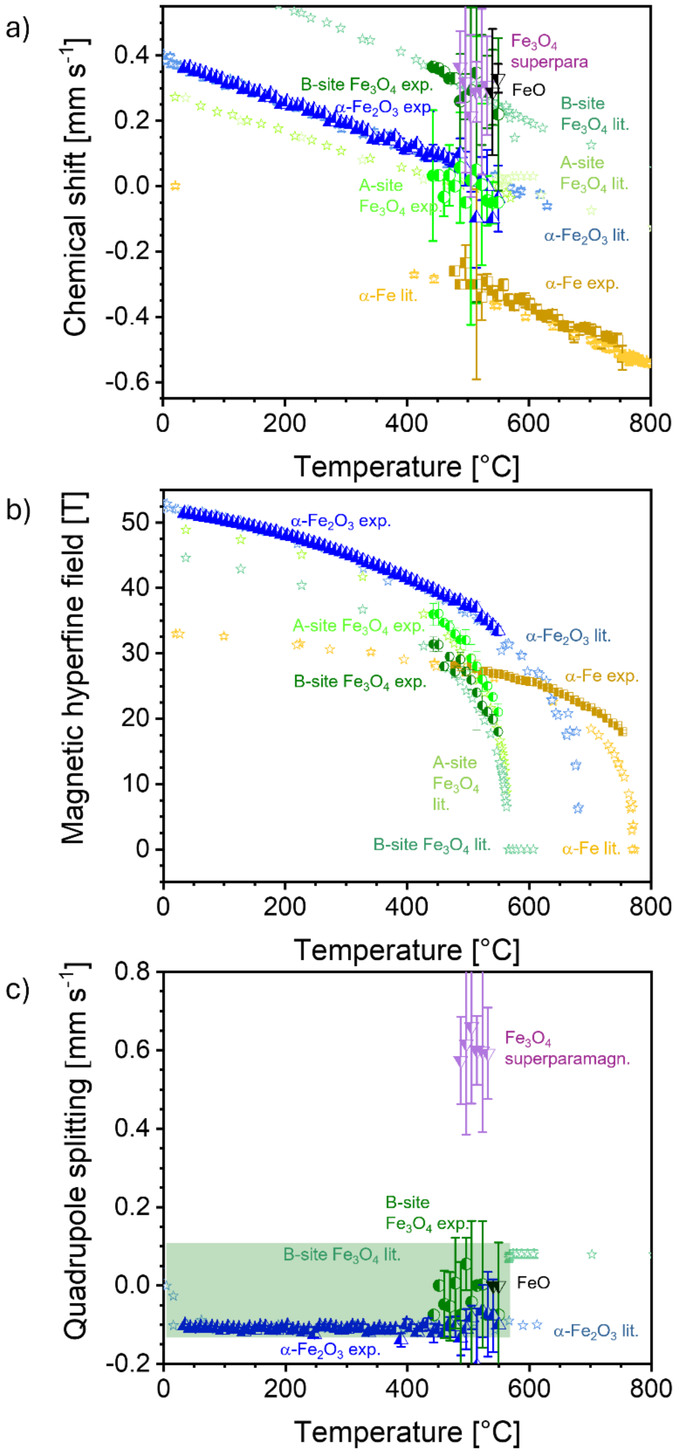

Figure S16. Hyperfine interaction parameters from Mössbauer spectroscopy for the reduction at 2 K·min^{-1}. a) chemical shift, b) magnetic hyperfine field and c) quadrupole splitting.

3.5 Comparison of Chemical shift and magnetic hyperfine field obtained at both heating ramps.

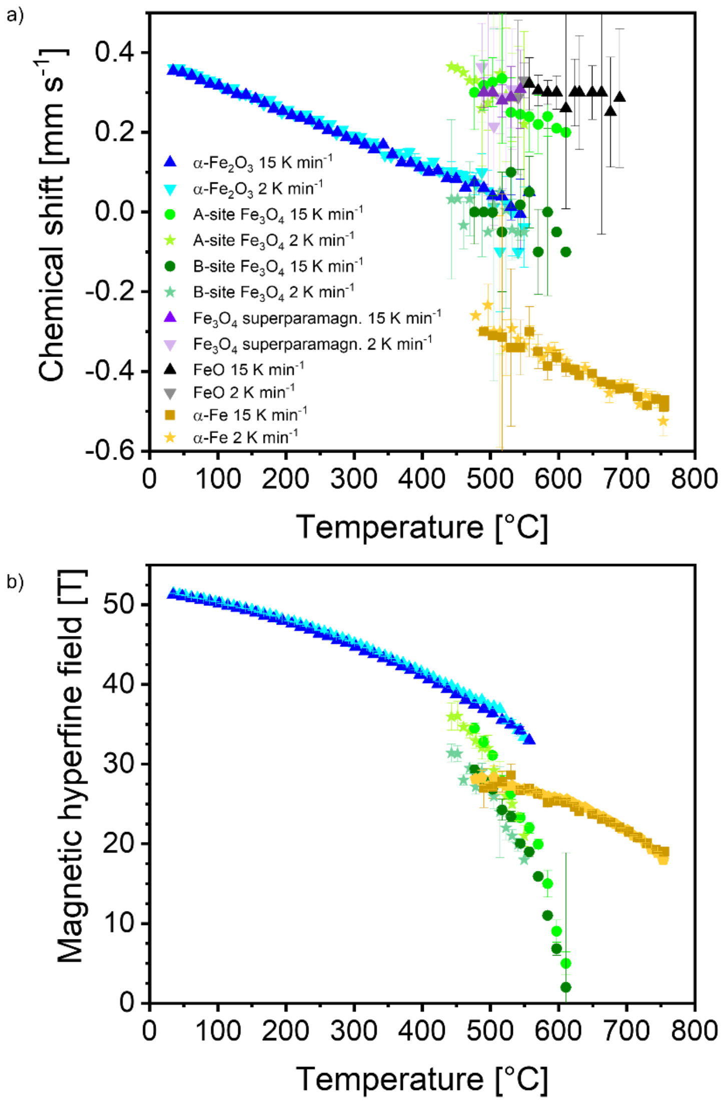

Figure S17. Comparison of Chemical shift a) and magnetic hyperfine field b) obtained at the experiments with the two different heating ramps, as indicated.

4 DFT and Baur-Gläsner diagram

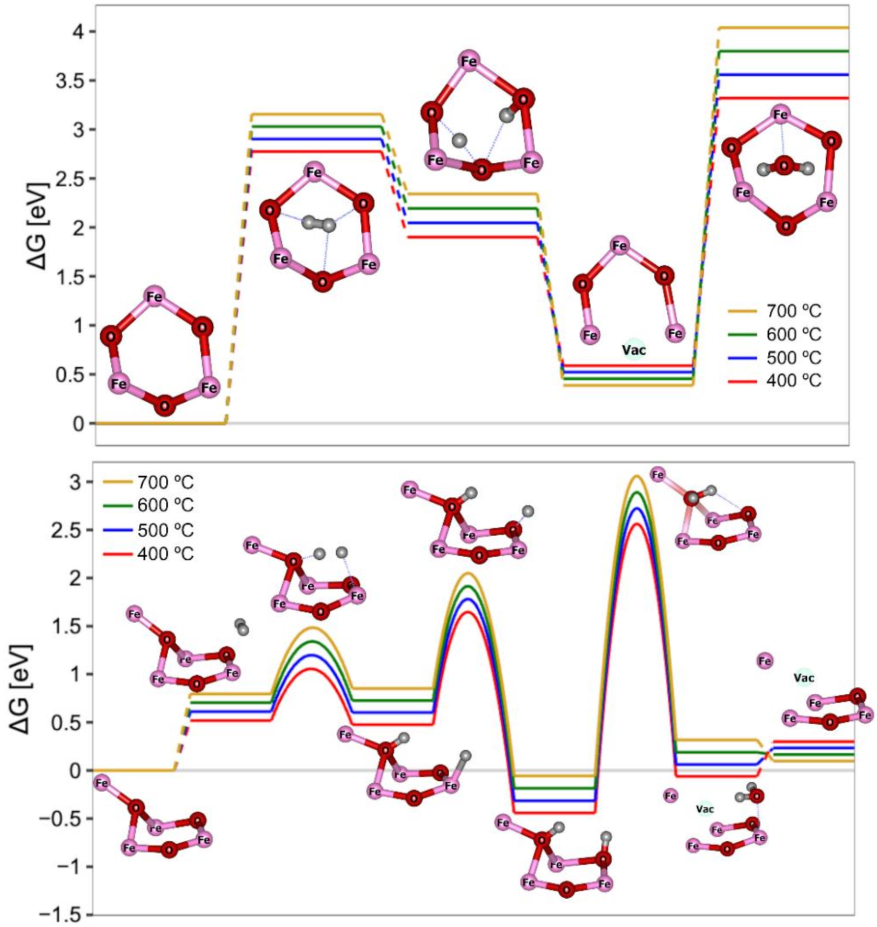

Figure S18. (a) Pathway of hydrogen adsorption on the hematite surface, b) Absorption of hydrogen and water in a hematite pore. Hydrogen bonds are depicted with dashed lines.

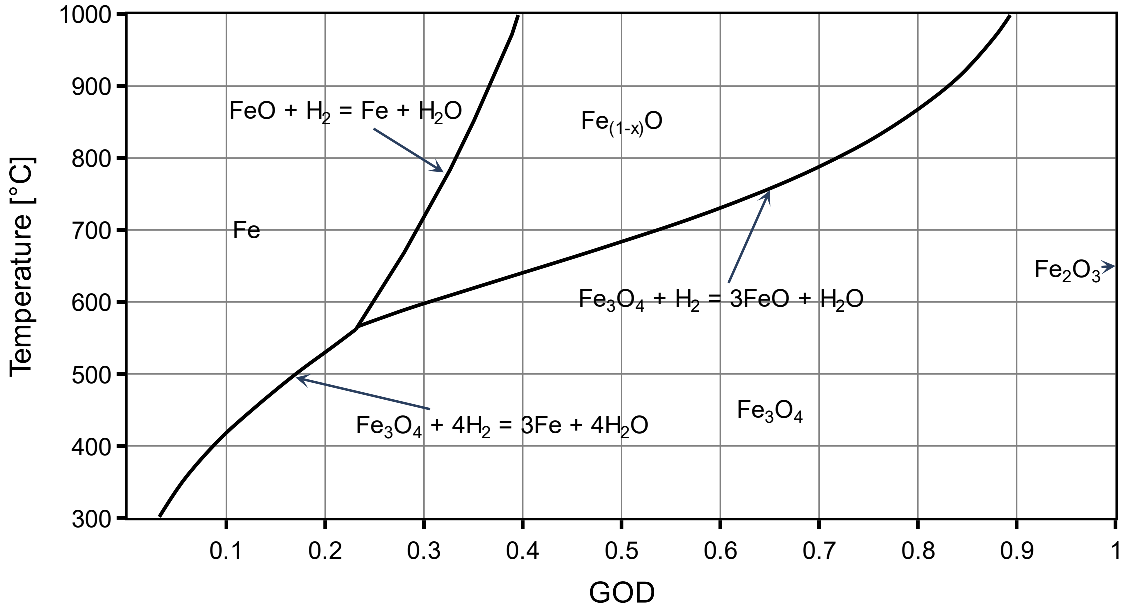

Figure S19. Baur-Gläsner diagram for the Fe-O-H system. GOD is defined as ratio of oxidized components to the sum of oxidized and oxidizable components (adapted from[2]).

[2] D. Spreitzer, J. Schenk, *Steel Res Int* **2019**, *90*.

5 Mössbauer parameter and composition calculation

Table S2: Hyperfine interaction parameters found for α-Fe_2O_3 during the reduction with a heating rate of 2 K·min^{-1} for the 3 µm particles by SMS. The temperature is a mean value over 5 min measurement time, so an uncertainty of ± 5 °C was assigned.

T / °C	CS / mm s^{-1}	error CS / mm s^{-1}	Lorentz width / mm s^{-1}	Error L / mm s^{-1}	Effective thickness	Error of effective thickness	QS / mm s^{-1}	Error of QS / mm s^{-1}	H / T	Error of H / T
RT - initial	0.381	0.002	0.248	0.006	5.820	0.059	-0.210	0.004	51.394	0.014
35.0	0.361	0.004	0.245	0.011	5.683	0.361	-0.204	0.008	51.425	0.025
43.9	0.361	0.004	0.242	0.012	5.841	0.361	-0.218	0.008	51.267	0.027
52.5	0.352	0.004	0.261	0.012	5.943	0.352	-0.220	0.008	51.140	0.028
61.1	0.349	0.004	0.249	0.012	5.705	0.349	-0.214	0.008	50.953	0.027
70.0	0.343	0.004	0.263	0.013	5.777	0.343	-0.218	0.008	50.828	0.029
79.3	0.336	0.004	0.274	0.012	5.565	0.336	-0.214	0.008	50.700	0.027
87.9	0.331	0.004	0.265	0.012	5.335	0.331	-0.218	0.008	50.532	0.028
96.6	0.326	0.004	0.267	0.012	5.510	0.326	-0.226	0.008	50.351	0.028
105.5	0.314	0.004	0.289	0.013	5.357	0.314	-0.216	0.008	50.169	0.029
114.6	0.311	0.004	0.265	0.012	5.089	0.311	-0.222	0.008	50.003	0.027
123.2	0.311	0.004	0.281	0.012	5.229	0.311	-0.222	0.008	49.831	0.027
131.9	0.296	0.004	0.299	0.013	5.232	0.296	-0.234	0.008	49.641	0.030
140.7	0.296	0.004	0.303	0.013	5.265	0.296	-0.218	0.008	49.513	0.030
149.8	0.287	0.004	0.291	0.013	5.332	0.287	-0.226	0.008	49.326	0.030
158.6	0.287	0.005	0.304	0.014	5.278	0.287	-0.212	0.010	49.109	0.032
167.3	0.271	0.005	0.312	0.016	5.097	0.271	-0.214	0.010	48.924	0.037
176.4	0.281	0.005	0.306	0.016	4.978	0.281	-0.226	0.010	48.703	0.035
185.3	0.268	0.008	0.330	0.023	5.032	0.268	-0.228	0.016	48.558	0.052
194.0	0.250	0.009	0.348	0.025	5.265	0.250	-0.218	0.016	48.198	0.058
203.2	0.257	0.006	0.287	0.017	4.742	0.257	-0.208	0.012	47.965	0.040
212.0	0.245	0.005	0.301	0.014	4.813	0.245	-0.228	0.010	47.838	0.032
220.7	0.245	0.006	0.360	0.017	4.969	0.245	-0.224	0.012	47.589	0.040
229.6	0.245	0.006	0.354	0.018	5.046	0.245	-0.236	0.012	47.346	0.041
238.5	0.230	0.007	0.397	0.022	5.040	0.230	-0.226	0.014	47.106	0.052
247.3	0.232	0.007	0.359	0.022	4.915	0.232	-0.250	0.014	46.826	0.053
256.2	0.221	0.007	0.346	0.021	4.920	0.221	-0.210	0.014	46.597	0.050
265.1	0.221	0.007	0.400	0.022	5.125	0.221	-0.212	0.014	46.346	0.053
274.0	0.197	0.007	0.349	0.020	4.665	0.197	-0.210	0.014	45.993	0.047
282.9	0.207	0.006	0.361	0.019	4.627	0.207	-0.218	0.012	45.753	0.044
291.7	0.193	0.006	0.367	0.018	4.660	0.193	-0.226	0.012	45.516	0.043
300.6	0.191	0.006	0.350	0.018	4.520	0.191	-0.224	0.012	45.168	0.042
309.5	0.189	0.007	0.337	0.021	4.254	0.189	-0.210	0.014	44.926	0.050
318.4	0.174	0.011	0.400	0.032	4.404	0.174	-0.214	0.022	44.610	0.077
327.3	0.175	0.008	0.344	0.023	4.217	0.175	-0.212	0.016	44.217	0.055
336.1	0.167	0.006	0.370	0.019	4.275	0.167	-0.238	0.012	43.873	0.044
345.0	0.143	0.007	0.381	0.021	4.300	0.143	-0.228	0.014	43.567	0.050
353.9	0.140	0.007	0.363	0.021	4.262	0.140	-0.218	0.014	43.265	0.050

362.8	0.149	0.008	0.400	0.025	4.345	0.149	-0.228	0.016	42.898	0.060
371.7	0.139	0.009	0.508	0.027	4.948	0.139	-0.228	0.018	42.677	0.067
380.5	0.151	0.010	0.464	0.028	4.435	0.151	-0.226	0.020	42.174	0.067
389.4	0.129	0.017	0.414	0.049	4.020	0.129	-0.278	0.034	41.990	0.120
398.3	0.111	0.012	0.358	0.035	3.747	0.111	-0.196	0.024	41.574	0.083
407.2	0.117	0.009	0.497	0.025	4.337	0.117	-0.214	0.018	41.211	0.063
416.1	0.127	0.009	0.436	0.025	4.250	0.127	-0.238	0.018	40.862	0.061
425.0	0.103	0.011	0.547	0.031	4.595	0.103	-0.212	0.022	40.353	0.077
433.9	0.093	0.016	0.513	0.045	4.169	0.093	-0.198	0.032	40.071	0.110
442.7	0.102	0.025	0.474	0.092	4.319	0.102	-0.242	0.048	39.791	0.217
451.6	0.095	0.010	0.311	0.044	2.941	0.095	-0.222	0.020	39.341	0.091
460.5	0.092	0.010	0.384	0.036	2.999	0.092	-0.240	0.020	38.873	0.072
469.4	0.083	0.017	0.401	0.064	2.754	0.083	-0.194	0.034	38.587	0.129
478.3	0.071	0.027	0.455	0.091	2.870	0.071	-0.210	0.054	38.010	0.192
487.2	0.100	0.046	0.547	0.152	2.952	0.100	-0.268	0.088	38.000	0.315
496.1	0.053	0.052	0.346	0.154	1.635	0.053	-0.222	0.102	37.307	0.333
504.9	0.013	0.030	0.098	fixed	1.234	0.013	-0.162	0.060	37.206	0.196
513.8	-0.098	0.152	0.798	0.465	1.631	-0.098	-0.410	0.292	36.880	0.967
522.7	0.023	0.066	0.323	0.157	1.164	0.023	-0.130	0.132	35.499	0.334
531.6	0.000	0.110	0.669	0.304	0.707	0.000	-0.146	0.216	34.986	0.625
540.5	-0.100	fixed	0.683	0.339	0.592	-0.100	-0.154	0.186	34.236	0.691
549.4	-0.038	0.101	0.600	0.369	0.487	-0.038	-0.200	fixed	33.420	0.739

Table S3: Hyperfine interaction parameters found for the A-site in Fe_3O_4 during the reduction with a heating rate of 2 K·min^{-1} by SMS. The temperature is a mean value over 5 min measurement time, so an uncertainty of ± 5 °C was assigned.

T / °C	CS / mm s^{-1}	error CS / mm s^{-1}	Lorentz width / mm s^{-1}	Error L / mm s^{-1}	Effective thickness	Error of effective thickness	QS / mm s^{-1}	Error of QS / mm s^{-1}	H / T	Error of H / T
442.7	0.032	0.200	0.198	0.446	0.259	0.189	0.000	fixed	35.954	1.686
451.6	0.033	fixed	0.544	0.373	0.314	0.109	0.000	fixed	36.062	1.793
460.5	-0.033	0.059	0.286	0.137	0.371	0.065	0.000	fixed	34.665	0.470
469.4	0.033	0.093	0.457	0.222	0.565	0.112	0.000	fixed	34.102	0.825
478.3	0.000	fixed	0.403	0.277	0.619	0.195	0.000	fixed	32.921	0.914
487.2	0.057	0.169	0.378	0.479	0.515	0.223	0.000	fixed	32.073	1.461
496.1	-0.050	fixed	0.250	0.452	0.282	0.082	0.000	fixed	32.000	1.618
504.9	0.018	0.442	0.400	0.764	0.359	0.438	0.000	fixed	29.238	2.098
513.8	0.050	0.409	0.248	0.856	0.201	0.575	0.000	fixed	28.000	2.332
522.7	0.005	0.247	0.303	0.469	0.328	0.228	0.000	fixed	25.997	1.830
531.6	-0.044	0.076	0.105	0.164	0.242	0.096	0.000	fixed	24.995	0.507
540.5	-0.051	0.064	0.107	0.153	0.200	0.069	0.000	fixed	23.321	0.438
549.4	-0.050	fixed	0.398	0.372	0.218	0.086	0.000	fixed	21.000	1.288

Table S4: Hyperfine interaction parameters found for the B-site in Fe_3O_4 during the reduction with a heating rate of 2 K·min^{-1} by SMS. The temperature is a mean value over 5 min measurement time, so an uncertainty of ± 5 °C was assigned.

T / °C	CS / mm s^{-1}	error CS / mm s^{-1}	Lorentz width / mm s^{-1}	Error L / mm s^{-1}	Effective thickness	Error of effective thickness	QS / mm s^{-1}	Error of QS / mm s^{-1}	H / T	Error of H / T
442.7	0.365	fixed	0.400	0.496	0.518	0.378	-0.150	fixed	31.398	1.134
451.6	0.360	fixed	0.800	0.374	0.628	0.219	0.000	fixed	31.317	1.016
460.5	0.350	fixed	0.800	0.241	0.741	0.130	-0.096	0.172	28.000	0.642
469.4	0.330	fixed	0.800	0.252	1.131	0.223	-0.106	0.174	29.500	0.684
478.3	0.330	fixed	0.800	0.388	1.238	0.391	0.012	0.232	27.146	0.928
487.2	0.260	0.144	0.559	0.454	1.030	0.446	-0.150	0.270	29.059	1.064
496.1	0.274	0.069	0.098	fixed	0.564	0.165	0.110	0.134	27.226	0.458
504.9	0.294	0.253	0.509	0.840	0.717	0.876	-0.084	0.414	25.930	1.559
513.8	0.347	0.702	0.688	1.968	0.402	1.151	0.000	fixed	24.000	5.703
522.7	0.294	0.168	0.500	0.480	0.656	0.456	0.006	0.322	22.010	1.487
531.6	0.288	0.111	0.500	0.288	0.484	0.191	-0.150	fixed	20.995	0.768
540.5	0.302	0.115	0.500	0.284	0.399	0.138	-0.150	fixed	19.904	0.796
549.4	0.220	0.233	0.800	0.540	0.437	0.172	-0.150	0.370	18.000	fixed

Table S5: Hyperfine interaction parameters found for the iron environment interpreted as superparamagnetic Fe_3O_4 formed during the reduction with a heating rate of 2 K·min^{-1} and identified by SMS. The temperature is a mean value over 5 min measurement time, so an uncertainty of ± 5 °C was assigned.

T / °C	CS / mm s^{-1}	error CS / mm s^{-1}	Lorentz width / mm s^{-1}	Error L / mm s^{-1}	Effective thickness	Error of effective thickness	QS / mm s^{-1}	Error of QS / mm s^{-1}
487.2	0.364	0.110	0.098	0.323	0.201	0.130	1.148	0.222
496.1	0.318	0.277	0.500	0.835	0.304	0.269	1.234	0.464
504.9	0.215	0.248	0.500	0.870	0.355	0.440	1.320	0.392
513.8	0.288	0.092	0.098	fixed	0.251	0.112	1.200	0.176
522.7	0.289	0.254	0.504	0.895	0.260	0.274	1.196	0.414
531.6	0.308	0.151	0.514	1.520	0.265	0.247	1.184	0.234

Table S6: Hyperfine interaction parameters found for FeO during the reduction with a heating rate of 2 K·min^{-1} by SMS. The temperature is a mean value over 5 min measurement time, so an uncertainty of ± 5 °C was assigned.

T / °C	CS / mm s^{-1}	error CS / mm s^{-1}	Lorentz width / mm s^{-1}	Error L / mm s^{-1}	Effective thickness	Error of effective thickness	QS / mm s^{-1}	Error of QS / mm s^{-1}
540.5	0.288	0.193	0.553	0.620	0.101	0.081	0.000	
549.4	0.330	0.044	0.159	0.139	0.157	0.053	0.000	

Table S7: Hyperfine interaction parameters found for α-Fe during the reduction with a heating rate of 2 K·min^{-1} by SMS. The temperature is a mean value over 5 min measurement time, so an uncertainty of ± 5 °C was assigned.

T / °C	CS / mm s^{-1}	error CS / mm s^{-1}	Lorentz width / mm s^{-1}	Error L / mm s^{-1}	Effective thickness	Error of effective thickness	QS / mm s^{-1}	Error of QS / mm s^{-1}	H / T	Error of H / T
478.3	-0.260	fixed	0.098	0.211	0.426	0.291	0.000	fixed	28.150	0.389
487.2	-0.300	fixed	0.098	fixed	0.257	0.257	0.000	fixed	28.306	0.937
496.1	-0.234	0.053	0.098	fixed	0.694	0.223	0.000	fixed	27.175	0.389
504.9	-0.300	fixed	0.800	0.621	1.509	1.489	0.000	fixed	28.281	1.498
513.8	-0.300	0.291	0.800	0.727	1.906	2.124	0.000	fixed	27.632	1.368
522.7	-0.340	0.070	0.403	0.243	1.671	0.833	0.000	fixed	27.298	0.433
531.6	-0.292	0.023	0.436	0.092	2.090	0.399	0.000	fixed	27.305	0.183
540.5	-0.319	0.013	0.407	0.056	2.747	0.294	0.000	fixed	26.921	0.095
549.4	-0.334	0.016	0.553	0.073	2.923	0.360	0.000	fixed	26.873	0.127
558.3	-0.302	0.017	0.695	0.043	3.953	0.137	0.000	fixed	26.688	0.128
567.1	-0.348	0.017	0.699	0.044	4.157	0.145	0.000	fixed	26.494	0.124
576.0	-0.365	0.014	0.620	0.036	3.988	0.129	0.000	fixed	26.189	0.105
584.9	-0.344	0.014	0.614	0.034	4.014	0.124	0.000	fixed	25.917	0.098
593.8	-0.349	0.022	0.653	0.056	4.047	0.194	0.000	fixed	25.823	0.159
601.6	-0.361	0.017	0.675	0.043	4.421	0.152	0.000	fixed	25.655	0.127
612.0	-0.376	0.013	0.653	0.170	4.024	0.208	0.000	fixed	25.576	0.096
620.9	-0.395	0.014	0.680	0.034	4.079	0.110	0.000	fixed	25.172	0.102
629.8	-0.401	0.014	0.705	0.035	4.371	0.114	0.000	fixed	24.755	0.101
638.6	-0.392	0.014	0.623	0.038	4.157	0.122	0.000	fixed	24.584	0.101
647.5	-0.409	0.015	0.679	0.036	4.273	0.118	0.000	fixed	24.051	0.106
656.4	-0.427	0.012	0.584	0.033	3.989	0.110	0.000	fixed	23.716	0.088
665.3	-0.430	0.013	0.565	0.032	3.834	0.113	0.000	fixed	23.372	0.094
674.2	-0.454	0.024	0.645	0.058	3.982	0.186	0.000	fixed	22.889	0.168
683.1	-0.439	0.017	0.656	0.047	3.925	0.135	0.000	fixed	22.521	0.126
692.0	-0.432	0.012	0.641	0.030	4.096	0.096	0.000	fixed	21.937	0.089
700.9	-0.437	0.015	0.654	0.037	3.958	0.111	0.000	fixed	21.726	0.106
709.7	-0.445	0.013	0.620	0.038	4.150	0.107	0.000	fixed	21.044	0.100
718.6	-0.483	0.014	0.636	0.035	4.046	0.103	0.000	fixed	20.769	0.100
727.5	-0.459	0.015	0.680	0.037	4.033	0.102	0.000	fixed	20.071	0.108
736.4	-0.465	0.015	0.626	0.036	3.919	0.103	0.000	fixed	19.329	0.105
745.3	-0.471	0.020	0.641	0.047	3.848	0.125	0.000	fixed	18.906	0.141
753.4	-0.525	0.037	0.730	0.089	4.401	0.221	0.000	fixed	17.979	0.259
30 min at 755 °C	-0.483	0.007	0.702	0.015	4.016	0.037	0.000	fixed	17.907	0.044
RT - final	0.003	0.002	0.303	0.006	7.9	0.074	0.004	0.004	32.918	0.016

Table S8: Hyperfine interaction parameters found for a-Fe_2O_3 during the reduction with a heating rate of 15 K·min^{-1} by SMS. The temperature is a mean value over 1 min measurement time, so an uncertainty of ± 7.5 °C is assigned.

T / °C	CS / mm s^{-1}	error CS / mm s^{-1}	Lorentz width / mm s^{-1}	Error L / mm s^{-1}	Effective thickness	Error of effective thickness	QS / mm s^{-1}	Error of QS / mm s^{-1}	H / T	Error of H / T
RT - initial	0.38	0.00	0.13	0.00	10.61	0.06	-0.200	0.000	51.36	0.01

34.5	0.354	0.003	0.154	0.01	10.501	0.158	-0.216	0.006	51.255	0.021
48.0	0.35	0.003	0.137	0.01	10.723	0.175	-0.214	0.006	51.061	0.021
61.8	0.342	0.003	0.131	0.01	10.558	0.187	-0.224	0.006	50.818	0.021
75.0	0.331	0.003	0.153	0.011	10.205	0.174	-0.218	0.006	50.595	0.024
88.5	0.321	0.003	0.149	0.01	10.1	0.165	-0.212	0.006	50.37	0.022
101.9	0.317	0.003	0.166	0.011	9.865	0.172	-0.214	0.006	50.144	0.024
114.7	0.306	0.004	0.19	0.011	9.996	0.176	-0.226	0.008	49.886	0.025
128.1	0.296	0.004	0.186	0.012	9.667	0.185	-0.220	0.008	49.586	0.027
141.3	0.294	0.004	0.14	0.013	9.505	0.194	-0.218	0.008	49.333	0.028
155.6	0.284	0.004	0.15	0.014	9.069	0.2	-0.216	0.008	49.024	0.03
168.4	0.273	0.004	0.172	0.012	9.021	0.172	-0.224	0.008	48.597	0.027
181.8	0.259	0.003	0.151	0.011	8.968	0.152	-0.224	0.006	48.277	0.023
195.0	0.253	0.003	0.164	0.011	8.956	0.156	-0.226	0.006	47.967	0.024
208.5	0.243	0.003	0.166	0.011	8.824	0.157	-0.218	0.006	47.607	0.025
221.7	0.237	0.004	0.164	0.012	8.762	0.163	-0.226	0.008	47.221	0.026
235.5	0.23	0.004	0.181	0.012	8.586	0.164	-0.218	0.008	46.871	0.027
248.6	0.217	0.004	0.17	0.012	8.68	0.165	-0.222	0.008	46.382	0.027
261.8	0.205	0.004	0.177	0.013	8.261	0.168	-0.202	0.008	46.07	0.028
275.5	0.2	0.004	0.184	0.013	8.445	0.178	-0.206	0.008	45.575	0.03
288.6	0.187	0.004	0.203	0.013	8.166	0.175	-0.226	0.008	45.225	0.03
302.2	0.18	0.004	0.204	0.013	8.094	0.17	-0.226	0.008	44.729	0.03
315.5	0.169	0.005	0.218	0.014	8.022	0.189	-0.210	0.010	44.158	0.034
329.0	0.157	0.005	0.226	0.015	7.858	0.192	-0.212	0.010	43.79	0.036
342.7	0.169	0.005	0.231	0.014	7.959	0.179	-0.228	0.010	43.292	0.033
356.0	0.145	0.005	0.238	0.015	7.622	0.184	-0.228	0.010	42.856	0.035
369.5	0.124	0.005	0.246	0.015	7.488	0.176	-0.220	0.010	42.269	0.035
382.7	0.123	0.005	0.235	0.015	7.396	0.178	-0.222	0.010	41.796	0.036
396.4	0.112	0.005	0.251	0.016	7.122	0.183	-0.214	0.010	41.205	0.038
409.6	0.101	0.006	0.262	0.017	7.247	0.192	-0.230	0.010	40.634	0.04
422.7	0.104	0.005	0.267	0.016	7.26	0.19	-0.226	0.010	40.023	0.04
436.5	0.085	0.006	0.291	0.017	7.09	0.19	-0.228	0.012	39.394	0.042
449.6	0.083	0.006	0.289	0.017	7.043	0.183	-0.206	0.012	38.749	0.042
463.1	0.061	0.006	0.293	0.019	6.592	0.196	-0.222	0.012	38.051	0.049
476.5	0.074	0.008	0.286	0.031	5.6	0.352	-0.200	0.016	37.48	0.067
490.1	0.06	0.009	0.229	0.03	4.235	0.257	-0.194	0.016	36.945	0.06
503.2	0.04	0.013	0.299	0.038	3.562	0.242	-0.160	0.024	36.36	0.08
516.9	0.039	0.019	0.398	0.064	3.259	0.368	-0.194	0.038	35.528	0.124
530.0	0.012	0.032	0.354	0.116	1.642	0.386	-0.134	0.064	34.97	0.212
543.7	-0.005	0.088	0.394	0.246	0.793	0.309	-0.174	0.174	34.246	0.494

Table S9: Hyperfine interaction parameters found for the A-site in Fe_3O_4 during the reduction with a heating rate of 15 K·min^{-1} by SMS. The temperature is a mean value over 1 min measurement time, so an uncertainty of ± 7.5 °C was assigned.

T / °C	CS / mm s^{-1}	error CS / mm s^{-1}	Lorentz width / mm s^{-1}	Error L / mm s^{-1}	Effective thickness	Error of effective thickness	QS / mm s^{-1}	Error of QS / mm s^{-1}	H / T	Error of H / T

476.5	0.000	0.080	0.164	0.176	0.345	0.120	0.000	fixed	34.500	0.680
490.1	0.000	fixed	0.400	0.193	0.680	0.119	0.000	fixed	32.805	0.666
503.2	-0.001	0.042	0.163	0.118	0.672	0.213	0.000	fixed	31.111	0.307
516.9	-0.050	0.150	0.398	0.284	0.710	0.414	0.000	fixed	27.903	0.702
530.0	0.100	0.089	0.400	0.197	0.998	0.249	0.000	fixed	26.346	0.753
543.7	0.018	0.088	0.398	0.349	0.979	0.726	0.000	fixed	23.287	0.562
556.9	0.050	0.090	0.397	0.224	0.788	0.158	0.000	fixed	22.028	0.680
570.0	-0.100	0.107	0.400	0.358	0.728	0.493	0.000	fixed	19.948	0.622
583.7	0.000	0.210	0.600	0.592	0.414	0.071	0.000	fixed	15.000	1.701
596.9	-0.050	fixed	0.262	0.512	0.233	0.079	0.000	fixed	9.043	1.422
610.6	-0.100	fixed	0.098	fixed	0.150	0.000	0.000	fixed	5.000	1.434

Table S10: Hyperfine interaction parameters found for the B-site in Fe_3O_4 during the reduction with a heating rate of 15 $K \cdot min^{-1}$ by SMS. The temperature is a mean value over 1 min measurement time, so an uncertainty of ± 7.5 °C was assigned.

T / °C	CS / mm s^{-1}	error CS / mm s^{-1}	Lorentz width / mm s^{-1}	Error L / mm s^{-1}	Effective thickness	Error of effective thickness	QS / mm s^{-1}	Error of QS / mm s^{-1}	H / T	Error of H / T
476.5	0.300	0.093	0.468	0.273	0.690	0.241	-0.012	0.184	29.276	0.642
490.1	0.318	0.062	0.554	0.176	1.361	0.237	-0.074	0.114	28.000	0.412
503.2	0.326	0.061	0.492	0.216	1.343	0.426	0.008	0.108	26.840	0.438
516.9	0.335	0.163	0.800	0.434	1.421	0.828	0.000	fixed	24.277	1.337
530.0	0.250	0.087	0.800	0.236	1.995	0.497	-0.110	0.134	23.421	0.647
543.7	0.245	fixed	0.500	0.278	1.362	0.588	0.000	fixed	20.055	0.522
556.9	0.239	0.088	0.690	0.272	1.576	0.317	-0.004	0.142	19.011	0.658
570.0	0.220	0.073	0.500	0.241	1.392	0.448	0.120	0.108	15.904	0.419
583.7	0.240	0.050	0.238	0.116	0.828	0.142	-0.102	0.086	11.004	0.317
596.9	0.210	fixed	0.293	0.289	0.465	0.158	0.000	fixed	6.843	0.844
610.6	0.200	fixed	0.500	3.526	0.300	0.000	0.000	fixed	2.000	16.873

Table S11: Hyperfine interaction parameters for the iron site interpreted as superparamagnetic Fe_3O_4 formed during the reduction with a heating rate of 15 K·min^{-1} by SMS. The temperature is a mean value over 1 min measurement time, so an uncertainty of ± 7.5 °C was assigned.

T / °C	CS / mm s^{-1}	error CS / mm s^{-1}	Lorentz width / mm s^{-1}	Error L / mm s^{-1}	Effective thickness	Error of effective thickness	QS / mm s^{-1}	Error of QS / mm s^{-1}
490.1	0.300	0.038	0.098	fixed	0.344	0.062	1.396	0.074
503.2	0.300	fixed	0.500	0.227	0.555	0.129	1.616	0.146
516.9	0.279	0.040	0.098	fixed	0.392	0.070	1.334	0.074
530.0	0.288	0.077	0.377	0.212	0.472	0.120	1.264	0.134

Table S12: Hyperfine interaction parameters found for FeO during the reduction with a heating rate of 15 K·min^{-1} by SMS. The temperature is a mean value over 1 min measurement time, so an uncertainty of ± 7.5 °C was assigned.

T / °C	CS / mm s^{-1}	error CS / mm s^{-1}	Lorentz width / mm s^{-1}	Error L / mm s^{-1}	Effective thickness	Error of effective thickness	QS / mm s^{-1}	Error of QS / mm s^{-1}
543.7	0.308	0.098	0.582	1.796	0.500	0.194	0.644	0.184
556.9	0.322	0.065	0.673	1.026	0.884	0.215	0.718	0.114
570.0	0.305	0.038	0.457	0.601	0.929	0.178	0.600	0.062
583.7	0.300	0.030	0.470	0.079	1.106	0.081	0.550	0.046
596.9	0.300	0.042	0.491	0.087	1.230	0.148	0.532	0.070
610.6	0.260	0.252	0.537	0.789	1.269	0.198	0.554	0.658
623.7	0.300	0.084	0.752	1.216	1.399	0.199	0.406	0.206
630.3	0.300	0.143	0.800	0.477	0.916	0.316	0.870	0.252
649.3	0.300	0.067	0.361	0.176	0.670	0.141	0.508	0.114
662.9	0.300	0.356	0.889	0.854	0.428	0.254	0.700	0.728
676.0	0.251	0.138	0.353	0.285	0.207	0.070	0.700	0.262

Table S13: Hyperfine interaction parameters found for a-Fe during the reduction with a heating rate of 15 K·min^{-1} by SMS. The temperature is a mean value over 1 min measurement time, so an uncertainty of ± 7.5 °C was assigned.

T / °C	CS / mm s^{-1}	error CS / mm s^{-1}	Lorentz width / mm s^{-1}	Error L / mm s^{-1}	Effective thickness	Error of effective thickness	QS / mm s^{-1}	Error of QS / mm s^{-1}	H / T	Error of H / T
490.1	-0.300	fixed	0.098	fixed	0.054	0.127	0.000	fixed	27.000	2.452
503.2	-0.310	fixed	0.627	0.765	0.550	0.700	0.000	fixed	27.186	1.441
516.9	-0.314	0.359	0.800	0.787	1.200	1.460	0.000	fixed	27.813	1.315
530.0	-0.340	0.198	0.800	0.692	1.107	0.972	0.000	fixed	28.624	1.428
543.7	-0.340	0.069	0.608	0.274	1.718	0.682	0.000	fixed	26.696	0.484
556.9	-0.300	0.063	0.729	0.199	2.076	0.465	0.000	fixed	26.966	0.436
570.0	-0.350	0.042	0.534	0.130	2.006	0.349	0.000	fixed	26.260	0.281
583.7	-0.387	0.034	0.600	0.086	2.702	0.253	0.000	fixed	25.149	0.217
596.9	-0.365	0.018	0.309	0.054	2.428	0.206	-0.026	fixed	25.351	0.136
610.6	-0.390	0.022	0.490	0.061	2.993	0.225	0.000	fixed	25.179	0.153
623.7	-0.396	0.012	0.255	0.035	3.654	0.217	0.000	fixed	24.613	0.083
630.3	-0.410	0.009	0.237	0.028	5.466	0.333	0.000	fixed	24.060	0.066
649.3	-0.405	0.010	0.247	0.028	4.230	0.216	0.000	fixed	23.723	0.073
662.9	-0.426	0.009	0.271	0.027	4.621	0.270	0.000	fixed	23.128	0.066
676.0	-0.433	0.007	0.268	0.021	5.397	0.173	0.000	fixed	22.692	0.056
689.6	-0.444	0.008	0.298	0.021	5.390	0.172	0.000	fixed	22.078	0.059
702.9	-0.442	0.008	0.299	0.020	5.887	0.167	0.000	fixed	21.491	0.057
716.0	-0.463	0.008	0.304	0.022	5.425	0.166	0.000	fixed	20.802	0.063
729.6	-0.486	0.008	0.317	0.021	5.742	0.162	0.000	fixed	20.086	0.062
742.8	-0.469	0.008	0.449	0.057	5.227	0.181	0.000	fixed	19.281	0.063
754.1	-0.490	0.009	0.332	0.023	5.720	0.168	0.000	fixed	19.088	0.067
754.9	-0.487	0.009	0.331	0.034	5.760	0.186	0.000	fixed	18.984	0.074
30 min at 755 °C	-0.472	0.003	0.337	0.008	5.549	0.059	-0.004	fixed	19.080	0.025
RT - final	-0.008	0.001	0.154	0.003	12.235	0.068	0.002	0.002	33.02	0.008

Calculation of sample composition and mass gain

Extraction of quantitative compositions from Mössbauer spectra is usually performed according to the following equation:

$$x_{i\%} = \frac{\frac{T_i}{f_i}}{\sum_j \frac{T_j}{f_j}} \cdot 100 \qquad \text{(Eq.1)}$$

where T_i represents the effective thickness of compound *i* and f_i is its Lamb-Mössbauer factor. To obtain the number percentage $x_{i\%}$ of iron atoms belonging to species *i,* the ratio $\frac{T_i}{f_i}$ is divided by the sum over all effective thicknesses, where each sub-spectrum T_j is weighted by their respective LMF f_j. In this study, f_i was set to 1 for all temperatures and species. Based on the similarity in LMF that are available at RT, we assume that the error in composition coming from this assumption is in the range of 2-3 wt%. With the stoichiometric coefficients v_i of 1 for Fe and FeO, 3 for Fe_3O_4 and 2 for Fe_2O_3, the number-% of iron atoms can be converted to mol-% according to:

$$n_{i\%} = \frac{\frac{x_{i\%}}{v_i}}{\sum_j \frac{x_{j\%}}{v_j}} \cdot 100 = \frac{\frac{x_{i\%}}{v_i}}{x_{Fe\%} + x_{FeO\%} + \frac{x_{Fe3O4\%}}{3} + \frac{x_{Fe2O3\%}}{2}} \cdot 100 \qquad \text{(Eq.2)}$$

.

Further weighting Eq.2 with the molar masses M_i of the compounds converts mol-% to mass-% $m_{i\%}$:

$$m_{i\%} = \frac{\frac{x_{i\%}}{v_i} \cdot M_i}{\sum_j (\frac{x_{j\%}}{v_j} \cdot M_j)} \cdot 100 \qquad \text{(Eq.3)}$$

From $x_{i\%}$ and the ratio of atoms "O" per atoms "Fe" in a species, the mass gain $\Delta m_{\%}$of a sample can be calculated:

$$\Delta m_{\%} = \frac{(x_{FeO\%} + x_{Fe3O4\%} \cdot \frac{4}{3} + x_{Fe2O3\%} \cdot \frac{3}{2}) \cdot M_O}{M_{Fe}} \qquad \text{(Eq.4)}$$

The values for $\boldsymbol{T_i}$, $\boldsymbol{x_{i\%}}$, $\boldsymbol{n_{i\%}}$, and $\boldsymbol{m_{i\%}}$ obtained for all samples are presented in Table S14 – S17.

Table S14: T_i and $x_{i\%}$ for the reduction with a heating rate 15 K·min^{-1} for the 3 µm particles. A) superparamagnetic Fe_3O_4 for T < 540 °C and FeO for T > 540 °C.

T_i									$x_{i\%}$							
**T**	**α-Fe**		**α-Fe_2O_3**		**Fe_3O_4**		**A**		**α-Fe**		**α-Fe_2O_3**		**Fe_3O_4**		**A**	
		error		error	sum	error		error		error		error	sum	error		error
°C																
463.1	0	0	6.6	0.2	0	0	0	0	0	0	100	0	0	0	0	0
476.5	0	0	5.6	0.4	1.0	0.4	0	0	0	0	84.4	5.0	15.6	4.8	0	0
490.1	0.1	0.1	4.2	0.3	2.0	0.4	0.3	0.1	0.8	2.0	63.5	4.9	30.6	4.4	5.2	0.0
503.2	0.6	0.7	3.6	0.2	2.0	0.6	0.6	0.1	8.2	10.1	53.3	8.4	30.2	7.7	8.3	0.1
516.9	1.2	1.5	3.3	0.4	2.1	1.2	0.4	0.1	17.2	17.9	46.7	13.5	30.5	14.1	5.6	0.2
530.0	1.1	1.0	1.6	0.4	3.0	0.7	0.5	0.1	17.8	13.3	26.4	7.3	48.2	10.4	7.6	0.3
543.7	1.7	0.7	0.8	0.3	2.3	1.3	0.5	0.2	32.1	12.5	14.8	6.6	43.7	15.4	9.3	1.1
556.9	2.1	0.5	0	0	2.4	0.5	0.9	0.2	39.0	9.4	0	0	44.4	7.1	16.6	1.3
570.0	2.0	0.3	0	0	2.1	0.9	0.9	0.2	39.7	11.5	0	0	41.9	11.7	18.4	1.1
583.7	2.7	0.3	0	0	1.2	0.2	1.1	0.1	53.5	12.5	0	0	24.6	5.5	21.9	0.7
596.9	2.4	0.2	0	0	0.7	0.2	1.2	0.1	55.7	16.4	0	0	16.0	6.7	28.2	1.4
610.6	3.0	0.2	0	0	0.5	0.0	1.3	0.2	63.5	17.4	0	0	9.6	5.8	26.9	2.0
623.7	3.7	0.2	0	0	0	0	1.4	0.2	72.3	20.2	0	0	0	0	27.7	2.1
630.3	5.5	0.3	0	0	0	0	0.9	0.3	85.6	12.4	0	0	0	0	14.4	3.6
649.3	4.2	0.2	0	0	0	0	0.7	0.1	86.3	11.9	0	0	0	0	13.7	2.1
662.9	4.6	0.3	0	0	0	0	0.4	0.3	91.5	7.8	0	0	0	0	8.5	4.2
676.0	5.4	0.2	0	0	0	0	0.2	0.1	96.3	3.6	0	0	0	0	3.7	1.2
689.6	5.4	0.2	0	0	0	0	0.1	0.1	99.0	1.0	0	0	0	0	1.0	1.1
702.9	5.9	0.2	0	0	0	0	0	0	100	0	0	0	0	0	0	0

Table S15: $n_{i,\%}$ and $m_{i\%}$ for the reduction with a heating rate of 15 K·min^{-1} for the 3 μm particles. A) superparamagnetic Fe_3O_4 for T < 540 °C and FeO for T > 540 °C.

	$n_{i,\%}$								$m_{i\%}$							
**T**	**α-Fe**		**α-Fe$_2$O$_3$**		**Fe$_3$O$_4$**		**A**		**α-Fe**		**α-Fe$_2$O$_3$**		**Fe$_3$O$_4$**		**A**	
		error		error	sum	error		error		error		error	sum	error		error
°C																
463.1	0	0	100	0	0	0	0	0	0	0	100	0	0	0	0	0
476.5	0	0	89.4	5.8	10.6	3.8	0	0	0	0	85.3	5.5	14.7	5.2	0	0
490.1	1.8	4.5	71.7	5.9	22.6	3.6	3.9	0.0	0.6	1.4	64.8	5.3	29.6	4.7	5.1	0.0
503.2	17.3	21.1	56.0	8.6	20.9	5.1	5.8	0.0	6.0	7.3	55.5	8.5	30.1	7.4	8.4	0.0
516.9	32.8	34.1	44.5	12.7	19.1	8.8	3.6	0.0	12.9	13.4	50.1	14.3	31.2	14.3	5.8	0.0
530.0	35.9	26.8	26.6	7.3	32.4	6.8	5.1	0.0	13.4	10.0	28.4	7.8	50.2	10.6	7.9	0.0
543.7	50.8	19.8	11.7	5.2	22.7	8.1	14.8	0.1	25.8	10.1	17.0	7.6	47.8	17.0	9.3	0.1
556.9	55.2	13.4	0	0	21.3	3.5	23.5	0.1	32.0	7.8	0	0	51.1	8.3	16.9	0.1
570.0	55.2	15.7	0	0	19.3	5.2	25.6	0.1	33.1	9.4	0	0	47.9	13.0	19.0	0.1
583.7	64.2	15.0	0	0	9.5	2.2	26.3	0.1	47.1	11.0	0	0	28.9	6.6	23.9	0.0
596.9	62.4	18.3	0	0	6.0	2.3	31.6	0.1	49.4	14.4	0	0	19.7	7.6	31.0	0.1
610.6	67.6	18.3	0	0	3.8	2.0	28.7	0.1	56.9	15.4	0	0	13.1	7.1	29.9	0.1
623.7	72.3	20.2	0	0	0	0	27.7	0.1	67.8	19.0	0	0	0	0	32.2	0.1
630.3	85.6	12.4	0	0	0	0	14.4	0.2	82.8	12.0	0	0	0	0	17.2	0.3
649.3	86.3	11.9	0	0	0	0	13.7	0.1	83.6	11.5	0	0	0	0	16.4	0.1
662.9	91.5	7.8	0	0	0	0	8.5	0.2	89.7	7.7	0	0	0	0	10.3	0.3
676.0	96.3	3.6	0	0	0	0	3.7	0.1	95.5	3.6	0	0	0	0	4.5	0.1
689.6	99.0	1.0	0	0	0	0	1.0	0.1	98.7	1.0	0	0	0	0	1.3	0.1
702.9	100	0	0	0	0	0	0	0	100	0	0	0	0	0	0	0

Table S16: T_i and $x_{i\%}$ for the reduction with a heating rate of 2 K·min^{-1} for the 3 µm particles. A) superparamagnetic Fe_3O_4 for T < 540 °C and FeO for T > 540 °C.

	T_i								$x_{i\%}$							
T	α-Fe		α-Fe_2O_3		Fe_3O_4		A		α-Fe		α-Fe_2O_3		Fe_3O_4		A	
°C		error		error	sum	error		error		error		error	sum	error		error
433.9	0	0	4.2	0.2	0	0	0	0	0	0	100	0	0	0	0	0
442.7	0	0	4.3	0.6	0.8	0.6	0	0	0	0	84.8	9.8	15.2	9.7	0	0
451.6	0	0	2.9	0.3	0.9	0.3	0	0	0	0	75.7	7.2	24.3	6.9	0	0
460.5	0	0	3.0	0.2	1.1	0.2	0	0	0	0	73.0	4.6	27.0	4.2	0	0
469.4	0	0	2.8	0.3	1.7	0.3	0	0	0	0	61.9	6.3	38.1	5.9	0	0
478.3	0.4	0.3	2.9	0.4	1.9	0.6	0	0	8.3	6.5	55.7	8.5	36.0	8.3	0	0
487.2	0.3	0.3	3.0	0.6	1.5	0.7	0.2	0.1	5.2	5.8	59.6	10.5	31.2	10.4	4.1	0.1
496.1	0.7	0.2	1.6	0.4	0.8	0.2	0.3	0.3	19.9	8.1	47.0	8.7	24.3	6.8	8.7	1.4
504.9	1.5	1.5	1.2	0.2	1.1	1.3	0.4	0.4	36.2	25.8	29.6	15.0	25.8	25.5	8.5	3.5
513.8	1.9	2.1	1.6	0.8	0.6	1.7	0.3	0.1	43.4	33.4	37.1	26.0	13.7	34.9	5.7	1.0
522.7	1.7	0.8	1.2	0.3	1.0	0.7	0.3	0.3	41.0	14.8	28.5	9.8	24.1	14.3	6.4	2.6
531.6	2.1	0.4	0.7	0.2	0.7	0.3	0.3	0.1	55.1	8.9	18.6	5.9	19.1	8.0	7.2	1.9
540.5	2.7	0.3	0.6	0.2	0.6	0.2	0.1	0.1	68.0	6.5	14.7	5.0	14.8	7.5	2.5	1.3
549.4	2.9	0.4	0.5	0.2	0.7	0.3	0.2	0.1	69.2	7.3	11.5	5.1	15.5	8.0	3.7	0.8
558.3	4.0	0.1	0	0	0	0	0	0	100	0	0	0	0	0	0	0

Table S17: $n_{i,\%}$ and $m_{i\%}$ for the reduction with a heating rate of 2 K·min^{-1} for the 3 µm particles. A) superparamagnetic Fe_3O_4 for T < 540 °C and FeO for T > 540 °C.

	$n_{i,\%}$								$m_{i\%}$							
T	α-Fe		α-Fe_2O_3		Fe_3O_4		A		α-Fe		α-Fe_2O_3		Fe_3O_4		A	
°C		error		error	sum	error		error		error		error	sum	error		error
433.9	0	0	100	0	0	0	0	0	0	0	100	0	0	0	0	0
442.7	0	0	89.0	10.8	11.0	7.1	0	0	0	0	84.8	10.3	15.2	9.9	0	0
451.6	0	0	83.1	7.3	16.9	4.7	0	0	0	0	77.2	6.8	22.8	6.3	0	0
460.5	0	0	80.4	5.1	19.6	3.1	0	0	0	0	73.8	4.7	26.2	4.1	0	0
469.4	0	0	70.8	6.8	29.2	4.2	0	0	0	0	62.6	6.0	37.4	5.4	0	0
478.3	17.1	13.4	57.5	8.9	25.4	5.8	0	0	6.0	4.7	57.3	8.9	36.7	8.4	0	0
487.2	11.2	12.6	64.2	11.6	21.7	7.8	2.9	0.0	3.8	4.2	61.8	11.2	30.4	10.8	4.1	0.0
496.1	36.9	15.0	43.5	7.8	14.2	3.7	5.4	0.0	15.2	6.2	51.3	9.2	24.3	6.2	9.2	0.1
504.9	57.8	41.2	23.6	11.9	14.0	13.4	4.5	0.1	28.6	20.4	33.4	16.8	28.8	27.5	9.3	0.2
513.8	63.4	48.6	27.1	18.9	6.7	16.7	2.8	0.0	35.2	27.0	43.1	30.0	15.3	38.5	6.4	0.1
522.7	62.5	22.8	21.8	7.5	12.5	7.4	3.2	0.1	32.9	12.0	32.8	11.3	27.2	16.1	7.1	0.1
531.6	75.5	12.3	12.8	4.1	8.4	3.8	3.3	0.0	47.0	7.6	22.7	7.2	21.8	9.8	8.5	0.1
540.5	82.1	7.8	8.9	3.0	6.0	3.0	3.0	0.1	60.4	5.7	18.6	6.3	18.2	9.0	2.8	0.1
549.4	82.2	9.1	6.8	3.1	6.6	3.4	4.4	0.0	61.1	6.8	14.6	6.5	20.2	10.4	4.1	0.0
558.3	100	0	0	0	0	0	0	0	100	0	0	0	0	0	0	0

6 DFT-Simulation: Hydrogen Adsorption on the Hematite (0001) Surface (Fe_2O_3) and its Reduction

Hydrogen Adsorption on the Hematite (0001)

1) Slab

DFT free energy: -820.506 eV

Frequencies: 635.302 cm^{-1}, 439.068 cm^{-1}, 309.349 cm^{-1}

Structure:

```
Fe2O3 slab
  1.00000000000000
   10.0776023865000006    0.0000000000000000    0.0000000000000000
   -5.0388011932000003    8.7274596759000005    0.0000000000000000
    0.0000000000000000    0.0000000000000000   28.7955398560000013
  Fe   O
   48    72
Selective dynamics
Direct
  0.0000000000000000  0.0000000000000000  0.2495813370000022
  0.0000000000000000  0.5000000000000000  0.2495813370000022
  0.5000000000000000  0.0000000000000000  0.2495813370000022
  0.5000000000000000  0.5000000000000000  0.2495813370000022
  0.0000000000000000  0.0000000000000000  0.3906557898659386
  0.0000000000000000  0.5000000000000000  0.3906557898659386
  0.5000000000000000  0.0000000000000000  0.3906557898659386
  0.5000000000000000  0.5000000000000000  0.3906557898659386
  0.0000000000000000  0.0000000000000000  0.1499616649999993
  0.0000000000000000  0.5000000000000000  0.1499616649999993
  0.5000000000000000  0.0000000000000000  0.1499616649999993
  0.5000000000000000  0.5000000000000000  0.1499616649999993
  0.0000000000000000  0.0000000000000000  0.4883290858473686
  0.0000000000000000  0.5000000000000000  0.4883290858473686
  0.5000000000000000  0.0000000000000000  0.4883290858473686
  0.5000000000000000  0.5000000000000000  0.4883290858473686
  0.3333333429999996  0.1666666719999981  0.4093525312582855
  0.3333333429999996  0.6666666870000029  0.4093525312582855
  0.8333333730000021  0.1666666719999981  0.4093525312582855
  0.8333333730000021  0.6666666870000029  0.4093525312582855
  0.3333333429999996  0.1666666719999981  0.5311880323100979
  0.3333333429999996  0.6666666870000029  0.5311880323100979
  0.8333333730000021  0.1666666719999981  0.5311880323100979
  0.8333333730000021  0.6666666870000029  0.5311880323100979
  0.3333333429999996  0.1666666719999981  0.3096570069999984
  0.3333333429999996  0.6666666870000029  0.3096570069999984
  0.8333333730000021  0.1666666719999981  0.3096570069999984
  0.8333333730000021  0.6666666870000029  0.3096570069999984
  0.3333333429999996  0.1666666719999981  0.1697336730000032
  0.3333333429999996  0.6666666870000029  0.1697336730000032
```

0.8333333730000021	0.1666666719999981	0.1697336730000032
0.8333333730000021	0.6666666870000029	0.1697336730000032
0.1666666719999981	0.3333333429999996	0.0898860100000007
0.1666666570000004	0.8333333730000021	0.0898860100000007
0.6666666870000029	0.3333333429999996	0.0898860100000007
0.6666666870000029	0.8333333730000021	0.0898860100000007
0.1666666719999981	0.3333333429999996	0.2298093140000006
0.1666666570000004	0.8333333730000021	0.2298093140000006
0.6666666870000029	0.3333333429999996	0.2298093140000006
0.6666666870000029	0.8333333730000021	0.2298093140000006
0.1666666719999981	0.3333333429999996	0.4759997728001437
0.1666666570000004	0.8333333730000021	0.4759997728001437
0.6666666870000029	0.3333333429999996	0.4759997728001437
0.6666666870000029	0.8333333730000021	0.4759997728001437
0.1666666719999981	0.3333333429999996	0.3299027079663119
0.1666666570000004	0.8333333730000021	0.3299027079663119
0.6666666870000029	0.3333333429999996	0.3299027079663119
0.6666666870000029	0.8333333730000021	0.3299027079663119
0.1533164379999974	0.0000000000000000	0.1997715090000014
0.1533164530000022	0.5000000000000000	0.1997715090000014
0.6533164379999974	0.0000000000000000	0.1997715090000014
0.6533164379999974	0.5000000000000000	0.1997715090000014
0.3439202819330092	0.9996499908857146	0.4409980346488780
0.3439202819330092	0.4996499908857146	0.4409980346488780
0.8439203709330130	0.9996499908857146	0.4409980346488780
0.8439203109330080	0.4996499908857146	0.4409980346488780
0.0000000000000000	0.1533164379999974	0.1997715090000014
0.0000000000000000	0.6533164379999974	0.1997715090000014
0.5000000000000000	0.1533164379999974	0.1997715090000014
0.5000000000000000	0.6533164379999974	0.1997715090000014
0.0003500091142854	0.3442702910472946	0.4409980346488780
0.0003500091142854	0.8442703200472934	0.4409980346488780
0.5003499791142829	0.3442702910472946	0.4409980346488780
0.5003500091142854	0.8442703200472934	0.4409980346488780
0.3466835919999980	0.3466835919999980	0.1997715090000014
0.3466835919999980	0.8466836209999968	0.1997715090000014
0.8466836209999968	0.3466835919999980	0.1997715090000014
0.8466836209999968	0.8466836209999968	0.1997715090000014
0.1557297389527008	0.1560797480669862	0.4409980346488780
0.1557297239527031	0.6560797480669862	0.4409980346488780
0.6557297389527008	0.1560797480669862	0.4409980346488780
0.6557297389527008	0.6560797480669862	0.4409980346488780
0.4866158232063498	0.1660040917926580	0.3600134567430686
0.4866157942063509	0.6660041067926628	0.3600134567430686
0.9866157942063509	0.1660040917926580	0.3600134567430686
0.9866157342063531	0.6660041067926628	0.3600134567430686
0.1800168899999974	0.1666666719999981	0.1199238370000018
0.1800168749999997	0.6666666870000029	0.1199238370000018
0.6800168749999997	0.1666666719999981	0.1199238370000018
0.6800168749999997	0.6666666870000029	0.1199238370000018

```
0.3339959232073397  0.3206117174136907  0.3600134567430686
0.3339959232073397  0.8206117474136931  0.3600134567430686
0.8339959532073422  0.3206117174136907  0.3600134567430686
0.8339959532073422  0.8206117474136931  0.3600134567430686
0.3333333429999996  0.0133502309999969  0.1199238370000018
0.3333333429999996  0.5133502480000018  0.1199238370000018
0.8333333730000021  0.0133502309999969  0.1199238370000018
0.8333333730000021  0.5133502480000018  0.1199238370000018
0.1793882675863046  0.0133841887936441  0.3600134567430686
0.1793882675863046  0.5133842057936491  0.3600134567430686
0.6793882525863069  0.0133841887936441  0.3600134567430686
0.6793882525863069  0.5133842057936491  0.3600134567430686
0.4866497519999982  0.3199830949999978  0.1199238370000018
0.4866497519999982  0.8199831250000003  0.1199238370000018
0.9866496920000003  0.3199830949999978  0.1199238370000018
0.9866497519999982  0.8199831250000003  0.1199238370000018
0.3270248619212381  0.3397952928436894  0.5207502034178648
0.3270248619212381  0.8397953228436918  0.5210974794213712
0.8270248919212406  0.3397952928436894  0.5210974794213712
0.8270248919212406  0.8397953228436918  0.5210974794213712
0.0133502199999995  0.3333333429999996  0.2796191569999991
0.0133502520000022  0.8333333730000021  0.2796191569999991
0.5133502480000018  0.3333333429999996  0.2796191569999991
0.5133502480000018  0.8333333730000021  0.2796191569999991
0.1602047221563083  0.4872295980775476  0.5210974794213712
0.1602047221563083  0.9872295690775488  0.5210974794213712
0.6602047371563131  0.4872295980775476  0.5210974794213712
0.6602047371563131  0.9872295690775488  0.5210974794213712
0.1666666719999981  0.1800168899999974  0.2796191569999991
0.1666666570000004  0.6800168749999997  0.2796191569999991
0.6666666870000029  0.1800168899999974  0.2796191569999991
0.6666666870000029  0.6800168749999997  0.2796191569999991
0.0127704079224529  0.1729751230787571  0.5210974794213712
0.0127703939224517  0.6729751080787594  0.5210974794213712
0.5127704309224512  0.1729751230787571  0.5210974794213712
0.5127704309224512  0.6729751080787594  0.5210974794213712
0.3199830949999978  0.4866497809999970  0.2796191569999991
0.3199830949999978  0.9866497519999982  0.2796191569999991
0.8199831250000003  0.4866497809999970  0.2796191569999991
0.8199831250000003  0.9866497519999982  0.2796191569999991
```

2) H_2 (molecule, ideal gas)

DFT free energy: -6.767 eV

Frequencies: 4313.674 cm^{-1}

Structure:

```
H2_gas
  1.00000000000000
   20.0000000000000000    0.0000000000000000    0.0000000000000000
    0.0000000000000000   20.0000000000000000    0.0000000000000000
```

0.0000000000000000 0.0000000000000000 20.0000000000000000

H

2

Direct

0.5000000000000000 0.5187634824812800 0.5000000000000000

0.5000000000000000 0.4812365175187200 0.5000000000000000

3) H_2 (molecule) on Fe atom of the Slab

DFT free energy: -827.487 eV

Frequencies: 4060.527 cm^{-1}, 780.244 cm^{-1}, 591.094 cm^{-1}, 464.243 cm^{-1}, 434.748 cm^{-1}, 373.503 cm^{-1}, 216.923 cm^{-1}, 171.558 cm^{-1}, 109.236 cm^{-1}

Structure:

H_2 on Fe Fe_2O_3

1.00000000000000

10.0776023865000006 0.0000000000000000 0.0000000000000000

-5.0388011932000003 8.7274596759000005 0.0000000000000000

0.0000000000000000 0.0000000000000000 28.7955398560000013

Fe O H

48 72 2

Selective dynamics

Direct

0.0000000000000000 0.0000000000000000 0.2495813370000022

0.0000000000000000 0.5000000000000000 0.2495813370000022

0.5000000000000000 0.0000000000000000 0.2495813370000022

0.5000000000000000 0.5000000000000000 0.2495813370000022

0.0008835002844094 0.9983299176298246 0.3918813501529215

0.9989043802886499 0.5017308135789165 0.3909508336230516

0.5009425188365171 0.9983522664339333 0.3917595858268967

0.4988319957895442 0.5015560080592749 0.3909618067521592

0.0000000000000000 0.0000000000000000 0.1499616649999993

0.0000000000000000 0.5000000000000000 0.1499616649999993

0.5000000000000000 0.0000000000000000 0.1499616649999993

0.5000000000000000 0.5000000000000000 0.1499616649999993

0.9991898710112821 0.9972005144486289 0.4905243603534544

0.0008695593178629 0.5027587450515654 0.4890149431058930

0.4988548779826019 0.9967565418383657 0.4904054013092747

0.5002635067389249 0.5029874512370540 0.4890646495478137

0.3350172665599302 0.1670561769956791 0.4072061101607645

0.3316120292468341 0.6660356923318815 0.4097121889420166

0.8351063374975425 0.1671402328173244 0.4071699725178988

0.8315708769677883 0.6660457126360413 0.4096647332372640

0.3335183800353718 0.1675511613891558 0.5094700308820137

0.3329062038395278 0.6662797085018184 0.5372664733741317

0.8331799347222599 0.1670487421254805 0.5092487270996600

0.8330169730584984 0.6661052511270213 0.5355427934582124

0.3333333429999996 0.1666666719999981 0.3096570069999984

0.3333333429999996 0.6666666870000029 0.3096570069999984

0.8333333730000021 0.1666666719999981 0.3096570069999984

0.8333333730000021	0.6666666870000029	0.3096570069999984
0.3333333429999996	0.1666666719999981	0.1697336730000032
0.3333333429999996	0.6666666870000029	0.1697336730000032
0.8333333730000021	0.1666666719999981	0.1697336730000032
0.8333333730000021	0.6666666870000029	0.1697336730000032
0.1666666719999981	0.3333333429999996	0.0898860100000007
0.1666666570000004	0.8333333730000021	0.0898860100000007
0.6666666870000029	0.3333333429999996	0.0898860100000007
0.6666666870000029	0.8333333730000021	0.0898860100000007
0.1666666719999981	0.3333333429999996	0.2298093140000006
0.1666666570000004	0.8333333730000021	0.2298093140000006
0.6666666870000029	0.3333333429999996	0.2298093140000006
0.6666666870000029	0.8333333730000021	0.2298093140000006
0.1697911375472856	0.3364414701805529	0.4774961800171980
0.1639837696614848	0.8307834873853466	0.4764936234795272
0.6696466847237943	0.3361199720414234	0.4775378482856567
0.6631354083484666	0.8305814345342739	0.4764862812010264
0.1666389404949484	0.3337236744987209	0.3299071477741364
0.1667759179776880	0.8330695620062301	0.3297891267033748
0.6666595627064353	0.3337123285228287	0.3299175716246907
0.6667901447841800	0.8330346487891944	0.3297940418369549
0.1533164379999974	0.0000000000000000	0.1997715090000014
0.1533164530000022	0.5000000000000000	0.1997715090000014
0.6533164379999974	0.0000000000000000	0.1997715090000014
0.6533164379999974	0.5000000000000000	0.1997715090000014
0.3442544666061593	0.0034146414426175	0.4423747841478090
0.3440789552368670	0.4994139655344298	0.4402719460778926
0.8442317929984426	0.0036785732877931	0.4425336980785133
0.8439698843790069	0.4997776413471513	0.4404624240984134
0.0000000000000000	0.1533164379999974	0.1997715090000014
0.0000000000000000	0.6533164379999974	0.1997715090000014
0.5000000000000000	0.1533164379999974	0.1997715090000014
0.5000000000000000	0.6533164379999974	0.1997715090000014
0.9965371947357866	0.3388307969621636	0.4426494149953371
0.9993275073910013	0.8450179765918122	0.4406132359033847
0.4963034947211966	0.3385733672225442	0.4427749180621063
0.4997163152421678	0.8453548759790763	0.4403698987561953
0.3466835919999980	0.3466835919999980	0.1997715090000014
0.3466835919999980	0.8466836209999968	0.1997715090000014
0.8466836209999968	0.3466835919999980	0.1997715090000014
0.8466836209999968	0.8466836209999968	0.1997715090000014
0.1633528025288911	0.1567841294940138	0.4427711277559538
0.1526925453282075	0.6549318382177205	0.4403757853476620
0.6635372005989666	0.1569431791253209	0.4427527008964987
0.6529732525009493	0.6549375755077733	0.4405152967413599
0.4884032940340859	0.1652794639921282	0.3603348580026164
0.4858025956999299	0.6661861220716006	0.3596902942368061
0.9884255463562681	0.1652563452307874	0.3603413498027379
0.9857463653688043	0.6662483096556144	0.3596786917232606
0.1800168899999974	0.1666666719999981	0.1199238370000018

0.1800168749999997 0.6666666870000029 0.1199238370000018
0.6800168749999997 0.1666666719999981 0.1199238370000018
0.6800168749999997 0.6666666870000029 0.1199238370000018
0.3347680017253225 0.3209284116939699 0.3596817088681021
0.3338824197750085 0.8214349861364241 0.3603028227334164
0.8348039968517327 0.3210651892768368 0.3596801675904473
0.8338712447200010 0.8215227619793595 0.3603549199293639
0.3333333429999996 0.0133502309999969 0.1199238370000018
0.3333333429999996 0.5133502480000018 0.1199238370000018
0.8333333730000021 0.0133502309999969 0.1199238370000018
0.8333333730000021 0.5133502480000018 0.1199238370000018
0.1793083054251170 0.0131873532955282 0.3598573114016901
0.1780290728259573 0.5128029674445216 0.3602279667380799
0.6794936823555915 0.0133503930322192 0.3598061314292025
0.6779970574811216 0.5127485499606195 0.3602429160201623
0.4866497519999982 0.3199830949999978 0.1199238370000018
0.4866497519999982 0.8199831250000003 0.1199238370000018
0.9866496920000003 0.3199830949999978 0.1199238370000018
0.9866497519999982 0.8199831250000003 0.1199238370000018
0.3282284829768827 0.3429160003614911 0.5210681112202451
0.3245043302250750 0.8343729382085812 0.5207651790346759
0.8284156920134365 0.3426695341432691 0.5209030236546965
0.8243787734809089 0.8350300260474413 0.5211019128295860
0.0133502199999995 0.3333333429999996 0.2796191569999991
0.0133502520000022 0.8333333730000021 0.2796191569999991
0.5133502480000018 0.3333333429999996 0.2796191569999991
0.5133502480000018 0.8333333730000021 0.2796191569999991
0.1628749401851834 0.4915107044940186 0.5207286707128347
0.1606652935071722 0.9842841461011034 0.5207291202613646
0.6618517953449867 0.4907515218545484 0.5210220255518010
0.6602148016975349 0.9841046343066537 0.5208006472596054
0.1666666719999981 0.1800168899999974 0.2796191569999991
0.1666666570000004 0.6800168749999997 0.2796191569999991
0.6666666870000029 0.1800168899999974 0.2796191569999991
0.6666666870000029 0.6800168749999997 0.2796191569999991
0.0141967106019507 0.1737868391175326 0.5204029740589888
0.0108026695877683 0.6734807620924883 0.5214931158593288
0.5141182301338745 0.1733893903414270 0.5203821258954306
0.5098814959971492 0.6737914615977303 0.5210793022946305
0.3199830949999978 0.4866497809999970 0.2796191569999991
0.3199830949999978 0.9866497519999982 0.2796191569999991
0.8199831250000003 0.4866497809999970 0.2796191569999991
0.8199831250000003 0.9866497519999982 0.2796191569999991
0.2876744711184784 0.6097233635171762 0.6095310717652609
0.3524421700215897 0.6928068522725611 0.6115151145631645

4) H_2 splitting (TS)

DFT free energy: -827.121 eV

Frequencies: 1444.887 cm^{-1}, 1380.381 cm^{-1}, 1187.433 cm^{-1}, 952.858 cm^{-1}, 576.415 cm^{-1}, 499.903 cm^{-1}, 423.765 cm^{-1}, 311.385 cm^{-1}

Structure:

H_2 splitting (TS)

1.00000000000000

10.0776023865000006 0.0000000000000000 0.0000000000000000

-5.0388011932000003 8.7274596759000005 0.0000000000000000

0.0000000000000000 0.0000000000000000 28.7955398560000013

Fe O H

48 72 2

Selective dynamics

Direct

0.0000000000000000 0.0000000000000000 0.2495813370000022

0.0000000000000000 0.5000000000000000 0.2495813370000022

0.5000000000000000 0.0000000000000000 0.2495813370000022

0.5000000000000000 0.5000000000000000 0.2495813370000022

0.9973039360687537 0.9960097693222139 0.3911276535845047

0.9999929281524942 0.5001403647719016 0.3898418093246931

0.5028461221097658 0.0008630955015150 0.3919504588967655

0.4984511734575676 0.5032364669734903 0.3900673971690907

0.0000000000000000 0.0000000000000000 0.1499616649999993

0.0000000000000000 0.5000000000000000 0.1499616649999993

0.5000000000000000 0.0000000000000000 0.1499616649999993

0.5000000000000000 0.5000000000000000 0.1499616649999993

0.0005379476746299 0.9991110154170144 0.4895080927396975

0.9987401553565363 0.5024431697817349 0.4870943379284327

0.4974776311848927 0.9934384897044879 0.4913194396742639

0.5065184287047302 0.5088840895863811 0.4876128688267727

0.3337057977447913 0.1651431467752786 0.4073367290934655

0.3320632301093127 0.6688017780475448 0.4100445219039557

0.8335223972901460 0.1666373593675843 0.4092592575893761

0.8342770912178921 0.6656286858532923 0.4096236266625084

0.3357401399460329 0.1735230801638821 0.5080869694999564

0.3339657667169575 0.6551974333445827 0.5409486745463212

0.8364713939457928 0.1688473939755681 0.5337893407425796

0.8335340765037103 0.6676996673586402 0.5346132941702848

0.3333333429999996 0.1666666719999981 0.3096570069999984

0.3333333429999996 0.6666666870000029 0.3096570069999984

0.8333333730000021 0.1666666719999981 0.3096570069999984

0.8333333730000021 0.6666666870000029 0.3096570069999984

0.3333333429999996 0.1666666719999981 0.1697336730000032

0.3333333429999996 0.6666666870000029 0.1697336730000032

0.8333333730000021 0.1666666719999981 0.1697336730000032

0.8333333730000021 0.6666666870000029 0.1697336730000032

0.1666666719999981 0.3333333429999996 0.0898860100000007

0.1666666570000004 0.8333333730000021 0.0898860100000007

0.6666666870000029 0.3333333429999996 0.0898860100000007

0.6666666870000029 0.8333333730000021 0.0898860100000007

0.1666666719999981	0.3333333429999996	0.2298093140000006
0.1666666570000004	0.8333333730000021	0.2298093140000006
0.6666666870000029	0.3333333429999996	0.2298093140000006
0.6666666870000029	0.8333333730000021	0.2298093140000006
0.1653173276349662	0.3401436179102291	0.4750077948470164
0.1671925032748902	0.8305711660663277	0.4782514842838594
0.6686253462100140	0.3299626238926265	0.4773654550476536
0.6679841002740190	0.8359947087993476	0.4752922247315396
0.1662462198254744	0.3330528498803034	0.3299033379481102
0.1672839545080080	0.8332917927788941	0.3299651064014029
0.6666005343958545	0.3334595074934228	0.3299000081580985
0.6669162902304535	0.8340739220384279	0.3295616000679900
0.1533164379999974	0.0000000000000000	0.1997715090000014
0.1533164530000022	0.5000000000000000	0.1997715090000014
0.6533164379999974	0.0000000000000000	0.1997715090000014
0.6533164379999974	0.5000000000000000	0.1997715090000014
0.3432109419756486	0.0024967668597782	0.4434920937032558
0.3455229050518085	0.4996165204968577	0.4394782888343087
0.8483349623887193	0.0009735540880484	0.4406107730756617
0.8417564327096656	0.4972388046474308	0.4404595954078019
0.0000000000000000	0.1533164379999974	0.1997715090000014
0.0000000000000000	0.6533164379999974	0.1997715090000014
0.5000000000000000	0.1533164379999974	0.1997715090000014
0.5000000000000000	0.6533164379999974	0.1997715090000014
0.0006307015803912	0.3455120298392060	0.4395079103343846
0.0010214923056679	0.8451870176156007	0.4409916070854152
0.4904499367416619	0.3332682305565484	0.4456631464174947
0.5020460394624067	0.8478052855819342	0.4403326184570133
0.3466835919999980	0.3466835919999980	0.1997715090000014
0.3466835919999980	0.8466836209999968	0.1997715090000014
0.8466836209999968	0.3466835919999980	0.1997715090000014
0.8466836209999968	0.8466836209999968	0.1997715090000014
0.1683651061967595	0.1616425346763322	0.4447732304999477
0.1528355923567446	0.6579720628647081	0.4396364414592426
0.6565368834694993	0.1553711843537187	0.4410842032574323
0.6551984377764128	0.6556377467156835	0.4397115770625675
0.4871060815769681	0.1659428987673195	0.3604006184343689
0.4864682358237999	0.6675807351035985	0.3595667469463066
0.9886335695558017	0.1653771206788193	0.3605000687144368
0.9854560835556825	0.6658551745513606	0.3595405428743703
0.1800168899999974	0.1666666719999981	0.1199238370000018
0.1800168749999997	0.6666666870000029	0.1199238370000018
0.6800168749999997	0.1666666719999981	0.1199238370000018
0.6800168749999997	0.6666666870000029	0.1199238370000018
0.3345883394764044	0.3207560041513986	0.3599212330231722
0.3346863839200722	0.8225409304747018	0.3604476703494015
0.8341499674702675	0.3200980478837394	0.3594754525885264
0.8334834888270919	0.8199288301024481	0.3597810192529849
0.3333333429999996	0.0133502309999969	0.1199238370000018
0.3333333429999996	0.5133502480000018	0.1199238370000018

```
0.8333333730000021  0.0133502309999969  0.1199238370000018
0.8333333730000021  0.5133502480000018  0.1199238370000018
0.1788330250042875  0.0131213182895991  0.3602400436313786
0.1799939835505953  0.5142899033663610  0.3596309974734027
0.6804849197763048  0.0138658341799029  0.3599426820470413
0.6771364346821400  0.5114755387644294  0.3605073441601334
0.4866497519999982  0.3199830949999978  0.1199238370000018
0.4866497519999982  0.8199831250000003  0.1199238370000018
0.9866496920000003  0.3199830949999978  0.1199238370000018
0.9866497519999982  0.8199831250000003  0.1199238370000018
0.3313194105022461  0.3540139838904253  0.5253622871680292
0.3276701226668521  0.8280227320669269  0.5226756560553127
0.8294960604784762  0.3403962253262520  0.5211623579482918
0.8270226566695840  0.8389629597920489  0.5211658767675473
0.0133502199999995  0.3333333429999996  0.2796191569999991
0.0133502520000022  0.8333333730000021  0.2796191569999991
0.5133502480000018  0.3333333429999996  0.2796191569999991
0.5133502480000018  0.8333333730000021  0.2796191569999991
0.1615806281723451  0.4932374889966837  0.5166210020781179
0.1644052977220909  0.9896363470885277  0.5206724674034930
0.6633167804529734  0.4907601130199595  0.5206071539341011
0.6656923397498034  0.9900202323860228  0.5211488839105627
0.1666666719999981  0.1800168899999974  0.2796191569999991
0.1666666570000004  0.6800168749999997  0.2796191569999991
0.6666666870000029  0.1800168899999974  0.2796191569999991
0.6666666870000029  0.6800168749999997  0.2796191569999991
0.0142733460828737  0.1761637678143728  0.5204171968785190
0.0106515881530029  0.6726442430261841  0.5213939110523995
0.5119891492159212  0.1712101694986785  0.5211607528776909
0.5138026714193273  0.6791693456962733  0.5178351112227020
0.3199830949999978  0.4866497809999970  0.2796191569999991
0.3199830949999978  0.9866497519999982  0.2796191569999991
0.8199831250000003  0.4866497809999970  0.2796191569999991
0.8199831250000003  0.9866497519999982  0.2796191569999991
0.3303691467085841  0.5103825912982813  0.5833148754614186
0.3263268287537642  0.4296269441971461  0.5589716453003989
```

5) 2H split and adsorbed on Fe and O

DFT free energy: -827.762 eV

Frequencies: 3721.503 cm^{-1}, 1605.703 cm^{-1}, 752.544 cm^{-1}, 534.335 cm^{-1}, 507.674 cm^{-1}, 471.883 cm^{-1}, 437.368 cm^{-1}, 383.403 cm^{-1}, 286.592 cm^{-1}

Structure:

```
H on Fe and H on O Fe2O3
   1.00000000000000
    10.0776023865000006    0.0000000000000000    0.0000000000000000
    -5.0388011932000003    8.7274596759000005    0.0000000000000000
     0.0000000000000000    0.0000000000000000   28.7955398560000013
```

Fe O H

48 72 2

Selective dynamics

Direct

0.0000000000000000 0.0000000000000000 0.2495813370000022

0.0000000000000000 0.5000000000000000 0.2495813370000022

0.5000000000000000 0.0000000000000000 0.2495813370000022

0.5000000000000000 0.5000000000000000 0.2495813370000022

0.9971482685254358 0.9957859541938205 0.3912838770971447

0.9995536924137198 0.5000268541051298 0.3904162058479415

0.5035618755539559 0.0014881663019537 0.3915856707906613

0.4988105674716437 0.5038619412021248 0.3903048082606873

0.0000000000000000 0.0000000000000000 0.1499616649999993

0.0000000000000000 0.5000000000000000 0.1499616649999993

0.5000000000000000 0.0000000000000000 0.1499616649999993

0.5000000000000000 0.5000000000000000 0.1499616649999993

0.9983649142372641 0.9976512405255846 0.4893297700564219

0.0046483983873316 0.5014722284454436 0.4890818642889343

0.5015153347517511 0.9939304917739449 0.4903159819247236

0.5086745326982296 0.5121511433599366 0.4882218769666267

0.3333457182240807 0.1674114021345332 0.4063487254286997

0.3326663399855860 0.6687176213479873 0.4102475578590301

0.8331711471708658 0.1653922159056549 0.4093187918850916

0.8341653269791962 0.6647792090681648 0.4098557669237479

0.3374966936676316 0.1585219232925610 0.5048468890793671

0.3334856938518342 0.6669628033833988 0.5442591135299253

0.8369990899262234 0.1675683201826388 0.5349895651355041

0.8346849356990802 0.6663361959735710 0.5356644515064204

0.3333333429999996 0.1666666719999981 0.3096570069999984

0.3333333429999996 0.6666666870000029 0.3096570069999984

0.8333333730000021 0.1666666719999981 0.3096570069999984

0.8333333730000021 0.6666666870000029 0.3096570069999984

0.3333333429999996 0.1666666719999981 0.1697336730000032

0.3333333429999996 0.6666666870000029 0.1697336730000032

0.8333333730000021 0.1666666719999981 0.1697336730000032

0.8333333730000021 0.6666666870000029 0.1697336730000032

0.1666666719999981 0.3333333429999996 0.0898860100000007

0.1666666570000004 0.8333333730000021 0.0898860100000007

0.6666666870000029 0.3333333429999996 0.0898860100000007

0.6666666870000029 0.8333333730000021 0.0898860100000007

0.1666666719999981 0.3333333429999996 0.2298093140000006

0.1666666570000004 0.8333333730000021 0.2298093140000006

0.6666666870000029 0.3333333429999996 0.2298093140000006

0.6666666870000029 0.8333333730000021 0.2298093140000006

0.1633999392300183 0.3392639002727975 0.4735585142883636

0.1655183271797327 0.8279366499555252 0.4776996223375960

0.6718491024782907 0.3320909191805654 0.4774996394190438

0.6662069086576849 0.8334625269562679 0.4767261008346892

0.1657352333902651 0.3332855145097255 0.3298487346076868

0.1670153241127039 0.8330312305742140 0.3299612376853034

0.6671302562995365	0.3337670162978341	0.3298799448366339
0.6671234377875876	0.8337926700178500	0.3295453368099430
0.1533164379999974	0.0000000000000000	0.1997715090000014
0.1533164530000022	0.5000000000000000	0.1997715090000014
0.6533164379999974	0.0000000000000000	0.1997715090000014
0.6533164379999974	0.5000000000000000	0.1997715090000014
0.3421775337050619	0.0063844847990921	0.4437289172739511
0.3457976928078637	0.4989893506028267	0.4396789689664473
0.8466455376468573	0.9994291418841499	0.4408432676257519
0.8433945594418049	0.4975509917000949	0.4409472858018901
0.0000000000000000	0.1533164379999974	0.1997715090000014
0.0000000000000000	0.6533164379999974	0.1997715090000014
0.5000000000000000	0.1533164379999974	0.1997715090000014
0.5000000000000000	0.6533164379999974	0.1997715090000014
0.0001712709617934	0.3450353880825787	0.4397865476613347
0.0002813525031300	0.8442172577444538	0.4407346913168553
0.4893348927447292	0.3328736711280413	0.4464427227262533
0.5029800201972208	0.8478420791745833	0.4401001915820402
0.3466835919999980	0.3466835919999980	0.1997715090000014
0.3466835919999980	0.8466836209999968	0.1997715090000014
0.8466836209999968	0.3466835919999980	0.1997715090000014
0.8466836209999968	0.8466836209999968	0.1997715090000014
0.1649174179786996	0.1599629222565042	0.4435238021271886
0.1528492954486822	0.6553668577039033	0.4400207770656763
0.6565525244615955	0.1555729263305707	0.4406998548821903
0.6545012263450758	0.6547670498148364	0.4402899287778084
0.4871448724707363	0.1662889500583944	0.3602362402891401
0.4865226373354616	0.6675533511565774	0.3596438645001641
0.9881653088672095	0.1652300756825227	0.3605117282060419
0.9854926581001564	0.6655831374661076	0.3596715992161066
0.1800168899999974	0.1666666719999981	0.1199238370000018
0.1800168749999997	0.6666666870000029	0.1199238370000018
0.6800168749999997	0.1666666719999981	0.1199238370000018
0.6800168749999997	0.6666666870000029	0.1199238370000018
0.3349564728848762	0.3218026411947079	0.3597373455490853
0.3344536077412812	0.8228609174961221	0.3607537479313692
0.8342918857275876	0.3201431047480483	0.3595966574114939
0.8329933958989173	0.8193012916681894	0.3598813328076531
0.3333333429999996	0.0133502309999969	0.1199238370000018
0.3333333429999996	0.5133502480000018	0.1199238370000018
0.8333333730000021	0.0133502309999969	0.1199238370000018
0.8333333730000021	0.5133502480000018	0.1199238370000018
0.1786708199033882	0.0132367182755786	0.3599569939431220
0.1799433283243133	0.5142974843709638	0.3598078012700370
0.6803472717184960	0.0137834026469221	0.3597769693541508
0.6770082250462082	0.5111430257201164	0.3607974906019180
0.4866497519999982	0.3199830949999978	0.1199238370000018
0.4866497519999982	0.8199831250000003	0.1199238370000018
0.9866496920000003	0.3199830949999978	0.1199238370000018
0.9866497519999982	0.8199831250000003	0.1199238370000018

0.3286297006514687 0.3401262278555208 0.5235476628563092

0.3265582979703154 0.8337001476242705 0.5196964253468010

0.8311490306433811 0.3390349463673203 0.5213815099095100

0.8266056668444435 0.8362586448959917 0.5212882857971834

0.0133502199999995 0.3333333429999996 0.2796191569999991

0.0133502520000022 0.8333333730000021 0.2796191569999991

0.5133502480000018 0.3333333429999996 0.2796191569999991

0.5133502480000018 0.8333333730000021 0.2796191569999991

0.1675251050624524 0.4896528518907743 0.5190835217681098

0.1603066627972538 0.9862786521202906 0.5206899716161644

0.6636833785321485 0.4902785276941657 0.5210341985037914

0.6670696146033990 0.9890272776144613 0.5213445050329355

0.1666666719999981 0.1800168899999974 0.2796191569999991

0.1666666570000004 0.6800168749999997 0.2796191569999991

0.6666666870000029 0.1800168899999974 0.2796191569999991

0.6666666870000029 0.6800168749999997 0.2796191569999991

0.0147715337406851 0.1751644764075024 0.5207318460581192

0.0109507917058451 0.6706403420846101 0.5217602796378529

0.5174247811801820 0.1710197011324155 0.5214565856768871

0.5095806628257478 0.6749584483774669 0.5198146697485697

0.3199830949999978 0.4866497809999970 0.2796191569999991

0.3199830949999978 0.9866497519999982 0.2796191569999991

0.8199831250000003 0.4866497809999970 0.2796191569999991

0.8199831250000003 0.9866497519999982 0.2796191569999991

0.3305919656642615 0.6518516584635137 0.6010266457332278

0.2896672371811349 0.3543278887310493 0.5526415631886255

6) H-migration from Fe to O (TS)

DFT free energy: -826.599 eV

Frequencies: 3724.179 cm^{-1}, 1208.828 cm^{-1}, 732.475 cm^{-1}, 532.749 cm^{-1}, 507.320 cm^{-1}, 484.951 cm^{-1}, 376.591 cm^{-1}, 288.426 cm^{-1}

Structure:

H migration Fe_2O_3

1.00000000000000

10.0776023865000006 0.0000000000000000 0.0000000000000000

-5.0388011932000003 8.7274596759000005 0.0000000000000000

0.0000000000000000 0.0000000000000000 28.7955398560000013

Fe O H

48 72 2

Selective dynamics

Direct

0.0000000000000000 0.0000000000000000 0.2495813370000022

0.0000000000000000 0.5000000000000000 0.2495813370000022

0.5000000000000000 0.0000000000000000 0.2495813370000022

0.5000000000000000 0.5000000000000000 0.2495813370000022

0.9980128206919616 0.9966535426596081 0.3912797471761209

0.9992338968200940 0.4992940157588990 0.3903871987469287

0.5030880558702364	0.0015369081576466	0.3915660059875776
0.4988637098134561	0.5036644824402785	0.3906540146925579
0.0000000000000000	0.0000000000000000	0.1499616649999993
0.0000000000000000	0.5000000000000000	0.1499616649999993
0.5000000000000000	0.0000000000000000	0.1499616649999993
0.5000000000000000	0.5000000000000000	0.1499616649999993
0.9994807808414308	0.9992288861246621	0.4890796573974043
0.0028863581360881	0.4976513896772872	0.4881797101159364
0.5037551059427017	0.9972207619113789	0.4899837311548652
0.5103960141632200	0.5115336023461481	0.4883202895395229
0.3324746361997626	0.1671990981079148	0.4062542450091371
0.3332998924435344	0.6687498804409771	0.4100598680576297
0.8331385616211051	0.1653925862884620	0.4091542527674932
0.8343900734051815	0.6651359423824204	0.4095934385681446
0.3376115284310401	0.1580848889348445	0.5047867417392808
0.3391263295809779	0.6730574164829051	0.5354370715459638
0.8366412153237661	0.1669719067524227	0.5348424337642754
0.8368753206739825	0.6671061822309596	0.5348272516513646
0.3333333429999996	0.1666666719999981	0.3096570069999984
0.3333333429999996	0.6666666870000029	0.3096570069999984
0.8333333730000021	0.1666666719999981	0.3096570069999984
0.8333333730000021	0.6666666870000029	0.3096570069999984
0.3333333429999996	0.1666666719999981	0.1697336730000032
0.3333333429999996	0.6666666870000029	0.1697336730000032
0.8333333730000021	0.1666666719999981	0.1697336730000032
0.8333333730000021	0.6666666870000029	0.1697336730000032
0.1666666719999981	0.3333333429999996	0.0898860100000007
0.1666666570000004	0.8333333730000021	0.0898860100000007
0.6666666870000029	0.3333333429999996	0.0898860100000007
0.6666666870000029	0.8333333730000021	0.0898860100000007
0.1666666719999981	0.3333333429999996	0.2298093140000006
0.1666666570000004	0.8333333730000021	0.2298093140000006
0.6666666870000029	0.3333333429999996	0.2298093140000006
0.6666666870000029	0.8333333730000021	0.2298093140000006
0.1627554957817239	0.3393323506202890	0.4746477085920944
0.1642010696529752	0.8280678010595608	0.4778087476115616
0.6724097212852911	0.3330003614525339	0.4772577067468831
0.6687029251250394	0.8343816710124798	0.4768010399873148
0.1657484231442226	0.3334048839485106	0.3298051863938340
0.1670111191418258	0.8329064027463886	0.3298538450050330
0.6669141462898196	0.3338353885349079	0.3297763019051203
0.6671794442861625	0.8337109182472915	0.3295527578667858
0.1533164379999974	0.0000000000000000	0.1997715090000014
0.1533164530000022	0.5000000000000000	0.1997715090000014
0.6533164379999974	0.0000000000000000	0.1997715090000014
0.6533164379999974	0.5000000000000000	0.1997715090000014
0.3421042801889698	0.0066332149210240	0.4436327458680509
0.3462295174318655	0.5018346472359596	0.4413510267631366
0.8462298088250506	0.9995836037670571	0.4409220518504569
0.8425816274645541	0.4975440515506904	0.4405056573985391

0.0000000000000000	0.1533164379999974	0.1997715090000014
0.0000000000000000	0.6533164379999974	0.1997715090000014
0.5000000000000000	0.1533164379999974	0.1997715090000014
0.5000000000000000	0.6533164379999974	0.1997715090000014
0.9986630262317959	0.3451385647607594	0.4395634099505230
0.0002465033324484	0.8433911045282088	0.4407933429895721
0.4892202359324571	0.3336671596513625	0.4461587582764395
0.5024996646051889	0.8470399986403407	0.4409113809108689
0.3466835919999980	0.3466835919999980	0.1997715090000014
0.3466835919999980	0.8466836209999968	0.1997715090000014
0.8466836209999968	0.3466835919999980	0.1997715090000014
0.8466836209999968	0.8466836209999968	0.1997715090000014
0.1624443062848826	0.1573123686964095	0.4427694368053494
0.1552971847516815	0.6548165038637066	0.4412787260679565
0.6562371071164890	0.1554910832860159	0.4406266203327718
0.6550134862994526	0.6551044212609227	0.4403505206314264
0.4870092728161239	0.1663525673557373	0.3602127219567990
0.4865886185068860	0.6674072648157576	0.3598709474813759
0.9877880965100729	0.1651865177690581	0.3603792468264828
0.9860384177002217	0.6655694071120521	0.3598036272345055
0.1800168899999974	0.1666666719999981	0.1199238370000018
0.1800168749999997	0.6666666870000029	0.1199238370000018
0.6800168749999997	0.1666666719999981	0.1199238370000018
0.6800168749999997	0.6666666870000029	0.1199238370000018
0.3348207857685424	0.3224880800700731	0.3599620877903931
0.3343229379078707	0.8227844134087618	0.3609034769512860
0.8339951664897995	0.3200599112599889	0.3594589215280948
0.8333618152471303	0.8196185320483593	0.3599207673518805
0.3333333429999996	0.0133502309999969	0.1199238370000018
0.3333333429999996	0.5133502480000018	0.1199238370000018
0.8333333730000021	0.0133502309999969	0.1199238370000018
0.8333333730000021	0.5133502480000018	0.1199238370000018
0.1787194446800342	0.0132354322999078	0.3597552854500137
0.1801700056654099	0.5143646322546971	0.3599579642003761
0.6797233117740831	0.0133219501576107	0.3598844254554052
0.6767212118787711	0.5113190372359924	0.3607298490066952
0.4866497519999982	0.3199830949999978	0.1199238370000018
0.4866497519999982	0.8199831250000003	0.1199238370000018
0.9866496920000003	0.3199830949999978	0.1199238370000018
0.9866497519999982	0.8199831250000003	0.1199238370000018
0.3304224962195121	0.3401618367672796	0.5238842419480392
0.3278263210867252	0.8441346002771937	0.5213624850503606
0.8300194719639791	0.3378558627772605	0.5214191107771455
0.8290459581173906	0.8377451313851765	0.5212640180936745
0.0133502199999995	0.3333333429999996	0.2796191569999991
0.0133502520000022	0.8333333730000021	0.2796191569999991
0.5133502480000018	0.3333333429999996	0.2796191569999991
0.5133502480000018	0.8333333730000021	0.2796191569999991
0.1607755464822702	0.4850958278893387	0.5245895693874445
0.1598752900264984	0.9873517960897615	0.5208947451092598

```
0.6655787004777309  0.4901768023483228  0.5209345825803240
0.6662676767776645  0.9880350764703181  0.5217451147630143
0.1666666719999981  0.1800168899999974  0.2796191569999991
0.1666666570000004  0.6800168749999997  0.2796191569999991
0.6666666870000029  0.1800168899999974  0.2796191569999991
0.6666666870000029  0.6800168749999997  0.2796191569999991
0.0149362937712070  0.1749831312228451  0.5212338628112434
0.0125137996750482  0.6700802903218275  0.5216566178699935
0.5185919948405200  0.1732852769395592  0.5213912932770626
0.5189988736906344  0.6752608746698741  0.5220551099839810
0.3199830949999978  0.4866497809999970  0.2796191569999991
0.3199830949999978  0.9866497519999982  0.2796191569999991
0.8199831250000003  0.4866497809999970  0.2796191569999991
0.8199831250000003  0.9866497519999982  0.2796191569999991
0.2944204584447405  0.3537152450243966  0.5539223868164598
0.2174133092817101  0.5395692510592910  0.5727707403226989
```

7) 2H adsorbed on 2O

DFT free energy: -828.820 eV

Frequencies: 3788.684 cm^{-1}, 3568.976 cm^{-1}, 836.360 cm^{-1}, 698.360 cm^{-1}, 519.510 cm^{-1}, 494.505 cm^{-1}, 403.370 cm^{-1}, 378.255 cm^{-1}, 309.870 cm^{-1}

Structure:

```
2H on 2O Fe2O3
   1.00000000000000
    10.0776023865000006    0.0000000000000000    0.0000000000000000
    -5.0388011932000003    8.7274596759000005    0.0000000000000000
     0.0000000000000000    0.0000000000000000   28.7955398560000013
   Fe   O    H
    48    72     2
Selective dynamics
Direct
  0.0000000000000000  0.0000000000000000  0.2495813370000022
  0.0000000000000000  0.5000000000000000  0.2495813370000022
  0.5000000000000000  0.0000000000000000  0.2495813370000022
  0.5000000000000000  0.5000000000000000  0.2495813370000022
  0.0027678311866879  0.9987515314851834  0.3925356024422939
  0.9980112538960526  0.4994078679996861  0.3908003108867746
  0.5013144241316283  0.9987827836223104  0.3925986614288277
  0.4987149591121707  0.5041701018823517  0.3905809908176323
  0.0000000000000000  0.0000000000000000  0.1499616649999993
  0.0000000000000000  0.5000000000000000  0.1499616649999993
  0.5000000000000000  0.0000000000000000  0.1499616649999993
  0.5000000000000000  0.5000000000000000  0.1499616649999993
  0.0011611796726285  0.0010840568752073  0.4910875711521783
  0.9964224019467451  0.5009181882634763  0.4879836053267965
  0.4984032147846875  0.9929215428965534  0.4914564109120789
  0.5088480097359778  0.5111781018535950  0.4883336315710380
  0.3341630244738454  0.1662974020862080  0.4062651948984453
```

0.3326684921865422	0.6676617207895958	0.4105130583693679
0.8333634857131784	0.1668588569483092	0.4063813044653202
0.8335009297881442	0.6673454951747075	0.4097378261430507
0.3365302920565796	0.1587259367216305	0.5052628743269878
0.3425317244010841	0.6774515761881474	0.5391828980335731
0.8327466350444297	0.1625727460417536	0.5060053021497453
0.8335298797647965	0.6674619734051674	0.5367124836128596
0.3333333429999996	0.1666666719999981	0.3096570069999984
0.3333333429999996	0.6666666870000029	0.3096570069999984
0.8333333730000021	0.1666666719999981	0.3096570069999984
0.8333333730000021	0.6666666870000029	0.3096570069999984
0.3333333429999996	0.1666666719999981	0.1697336730000032
0.3333333429999996	0.6666666870000029	0.1697336730000032
0.8333333730000021	0.1666666719999981	0.1697336730000032
0.8333333730000021	0.6666666870000029	0.1697336730000032
0.1666666719999981	0.3333333429999996	0.0898860100000007
0.1666666570000004	0.8333333730000021	0.0898860100000007
0.6666666870000029	0.3333333429999996	0.0898860100000007
0.6666666870000029	0.8333333730000021	0.0898860100000007
0.1666666719999981	0.3333333429999996	0.2298093140000006
0.1666666570000004	0.8333333730000021	0.2298093140000006
0.6666666870000029	0.3333333429999996	0.2298093140000006
0.6666666870000029	0.8333333730000021	0.2298093140000006
0.1685658495133922	0.3389265424227688	0.4739793957995957
0.1633506864827012	0.8286243118535381	0.4781595751988945
0.6698079905476106	0.3346906979108724	0.4786085874698713
0.6639131032576557	0.8304449045572682	0.4782719660832555
0.1662695807565413	0.3336341348679923	0.3299563919050783
0.1669064662884878	0.8324028086294106	0.3298488325873947
0.6659613860136844	0.3338986790484810	0.3297669141637414
0.6677648350940615	0.8337738246379089	0.3297653839072154
0.1533164379999974	0.0000000000000000	0.1997715090000014
0.1533164530000022	0.5000000000000000	0.1997715090000014
0.6533164379999974	0.0000000000000000	0.1997715090000014
0.6533164379999974	0.5000000000000000	0.1997715090000014
0.3434560686470078	0.0040667860669430	0.4431953722957260
0.3502496012537790	0.5042701323973731	0.4407579930439880
0.8443827382467575	0.0077990328804347	0.4441081641345335
0.8413029856059282	0.4999038899273600	0.4409364551218999
0.0000000000000000	0.1533164379999974	0.1997715090000014
0.0000000000000000	0.6533164379999974	0.1997715090000014
0.5000000000000000	0.1533164379999974	0.1997715090000014
0.5000000000000000	0.6533164379999974	0.1997715090000014
0.9890573314122690	0.3417708758145466	0.4409024545045384
0.9989173214487082	0.8436849308742751	0.4412656968273296
0.4886452397333585	0.3331686160437997	0.4463251355841820
0.5009439926025010	0.8458874457421715	0.4410943774487208
0.3466835919999980	0.3466835919999980	0.1997715090000014
0.3466835919999980	0.8466836209999968	0.1997715090000014
0.8466836209999968	0.3466835919999980	0.1997715090000014

0.8466836209999968	0.8466836209999968	0.1997715090000014
0.1617817930435734	0.1490908187870232	0.4418960101942062
0.1536383157246064	0.6510849396591993	0.4425945422416362
0.6621657868140858	0.1564014126563293	0.4428775788969048
0.6550676985591650	0.6559945590691143	0.4406819702089564
0.4887232959629060	0.1657180556225910	0.3606610314974361
0.4863521753779381	0.6675343184671334	0.3595869459964547
0.9882855816956067	0.1649009599055873	0.3604249398707964
0.9861239963308037	0.6659169525901731	0.3600376971035217
0.1800168899999974	0.1666666719999981	0.1199238370000018
0.1800168749999997	0.6666666870000029	0.1199238370000018
0.6800168749999997	0.1666666719999981	0.1199238370000018
0.6800168749999997	0.6666666870000029	0.1199238370000018
0.3352924402326352	0.3219543029142997	0.3598223947462245
0.3334538692954396	0.8216125208990803	0.3611067002471273
0.8338662941192538	0.3210056339555862	0.3590059061653221
0.8341892782989717	0.8219902047145098	0.3607214482021490
0.3333333429999996	0.0133502309999969	0.1199238370000018
0.3333333429999996	0.5133502480000018	0.1199238370000018
0.8333333730000021	0.0133502309999969	0.1199238370000018
0.8333333730000021	0.5133502480000018	0.1199238370000018
0.1794489533188326	0.0128587071561270	0.3593997765464891
0.1787916441431747	0.5131725041105142	0.3604539927717525
0.6785771021914826	0.0129022038294764	0.3598546623805703
0.6769969110951877	0.5121175159922444	0.3606436657747523
0.4866497519999982	0.3199830949999978	0.1199238370000018
0.4866497519999982	0.8199831250000003	0.1199238370000018
0.9866496920000003	0.3199830949999978	0.1199238370000018
0.9866497519999982	0.8199831250000003	0.1199238370000018
0.3348484579099562	0.3411622316157548	0.5256149446260636
0.3222356547419594	0.8420848309156028	0.5221063835944051
0.8300939607492310	0.3388739124342734	0.5209365120944369
0.8284165555501275	0.8374934848722688	0.5216568245382049
0.0133502199999995	0.3333333429999996	0.2796191569999991
0.0133502520000022	0.8333333730000021	0.2796191569999991
0.5133502480000018	0.3333333429999996	0.2796191569999991
0.5133502480000018	0.8333333730000021	0.2796191569999991
0.1507834794504817	0.4855908657344514	0.5316317306633849
0.1605167443704048	0.9870070856883473	0.5218180441296738
0.6645409650130389	0.4906019611870747	0.5215637992569384
0.6608864410579827	0.9830251402452319	0.5218811017290648
0.1666666719999981	0.1800168899999974	0.2796191569999991
0.1666666570000004	0.6800168749999997	0.2796191569999991
0.6666666870000029	0.1800168899999974	0.2796191569999991
0.6666666870000029	0.6800168749999997	0.2796191569999991
0.0162842078122480	0.1780525953434591	0.5218402229518091
0.0086999313373255	0.6712882768938329	0.5217591110292830
0.5164489081488171	0.1725034784531303	0.5215806232236275
0.5177612848978868	0.6719006773611440	0.5233990850484815
0.3199830949999978	0.4866497809999970	0.2796191569999991

0.3199830949999978 0.9866497519999982 0.2796191569999991

0.8199831250000003 0.4866497809999970 0.2796191569999991

0.8199831250000003 0.9866497519999982 0.2796191569999991

0.3081080490417136 0.3541517047949867 0.5569797992750765

0.0924666922089301 0.4033946672991107 0.5534799772772234

8) 2H adsorbed on 1O (TS)

DFT free energy: -827.396 eV

Frequencies: 3417.854 cm^{-1}, 2932.040 cm^{-1}, 1487.763 cm^{-1}, 843.264 cm^{-1}, 679.298 cm^{-1}, 440.877 cm^{-1}, 346.838 cm^{-1}, 244.325 cm^{-1}

Structure:

```
2H on 1O
  1.00000000000000
   10.0776020000000006    0.0000000000000000    0.0000000000000000
   -5.0388010000000003    8.7274600000000007    0.0000000000000000
    0.0000000000000000    0.0000000000000000   28.7955399999999990
  Fe   O    H
  48   72    2
Selective dynamics
Direct
  0.0000000000000000  0.0000000000000000  0.2501402199999987
  0.0000000000000000  0.5000000000000000  0.2501402199999987
  0.5000000000000000  0.0000000000000000  0.2501402199999987
  0.5000000000000000  0.5000000000000000  0.2501402199999987
  0.9997468199999986  0.9986491499999985  0.3879513700000032
  0.9990730700000015  0.5005550899999989  0.3901624299999966
  0.4997487899999982  0.9990208299999992  0.3881624899999991
  0.4995803099999989  0.4994165600000002  0.3906962900000011
  0.0000000000000000  0.0000000000000000  0.1492451100000025
  0.0000000000000000  0.5000000000000000  0.1492451100000025
  0.5000000000000000  0.0000000000000000  0.1492451100000025
  0.5000000000000000  0.5000000000000000  0.1492451100000025
  0.0036294399999974  0.0009280399999980  0.4841502000000020
  0.0010657500000022  0.5011288299999990  0.4908803300000031
  0.5040194600000021  0.0035516000000015  0.4842119600000032
  0.5140545599999982  0.5124023799999975  0.4858499999999992
  0.3350137999999987  0.1669688200000010  0.4096951200000021
  0.3341337800000019  0.6665083500000009  0.4100103600000011
  0.8326157899999984  0.1653084100000015  0.4092102999999980
  0.8335190000000026  0.6661458999999965  0.4100424800000013
  0.3352092599999992  0.1402454999999989  0.5349634399999985
  0.3379985700000034  0.6768220100000022  0.5321650899999995
  0.8350025499999987  0.1593986500000000  0.5339815199999975
  0.8368106900000001  0.6738950700000004  0.5321150400000008
  0.3333333400000029  0.1666666699999979  0.3088353600000033
  0.3333333400000029  0.6666666899999996  0.3088353600000033
  0.8333333699999983  0.1666666699999979  0.3088353600000033
  0.8333333699999983  0.6666666899999996  0.3088353600000033
```

0.3333333400000029	0.1666666699999979	0.1703451099999995
0.3333333400000029	0.6666666899999996	0.1703451099999995
0.8333333699999983	0.1666666699999979	0.1703451099999995
0.8333333699999983	0.6666666899999996	0.1703451099999995
0.1666666699999979	0.3333333400000029	0.0905499799999987
0.1666666699999979	0.8333333699999983	0.0905499799999987
0.6666666899999996	0.3333333400000029	0.0905499799999987
0.6666666899999996	0.8333333699999983	0.0905499799999987
0.1666666699999979	0.3333333400000029	0.2290402300000025
0.1666666699999979	0.8333333699999983	0.2290402300000025
0.6666666899999996	0.3333333400000029	0.2290402300000025
0.6666666899999996	0.8333333699999983	0.2290402300000025
0.1576445999999976	0.3344906100000031	0.4692983899999987
0.1691238800000008	0.8365218900000002	0.4758043100000009
0.6641615300000012	0.3324996899999988	0.4752334100000013
0.6687576500000034	0.8339553699999982	0.4756604100000033
0.1674234799999965	0.3342336099999983	0.3297496100000004
0.1660691299999968	0.8322600099999988	0.3298573399999967
0.6670636699999974	0.3341838500000023	0.3298116599999972
0.6659443699999983	0.8320811199999980	0.3298580100000024
0.1548490699999974	0.0000000000000000	0.1996926799999983
0.1548490699999974	0.5000000000000000	0.1996926799999983
0.6548490500000028	0.0000000000000000	0.1996926799999983
0.6548490500000028	0.5000000000000000	0.1996926799999983
0.3458368300000032	0.9992201299999977	0.4388633399999975
0.3420482000000007	0.4986423900000005	0.4416739099999987
0.8463596300000020	0.0007553499999986	0.4391666499999971
0.8457573199999970	0.4993626099999986	0.4418866500000007
0.0000000000000000	0.1548490699999974	0.1996926799999983
0.0000000000000000	0.6548490500000028	0.1996926799999983
0.4999999699999975	0.1548490699999974	0.1996926799999983
0.5000000000000000	0.6548490500000028	0.1996926799999983
0.9975628899999975	0.3452330799999999	0.4408874200000028
0.0025750799999997	0.8442375699999971	0.4393594200000024
0.4982868000000025	0.3437680700000030	0.4409448800000035
0.5026135000000025	0.8438713800000031	0.4396457799999993
0.3451509499999972	0.3451509499999972	0.1996926799999983
0.3451509499999972	0.8451509499999972	0.1996926799999983
0.8451509499999972	0.3451509499999972	0.1996926799999983
0.8451509499999972	0.8451509499999972	0.1996926799999983
0.1595468800000006	0.1610742299999970	0.4398615300000017
0.1567076700000030	0.6566376600000012	0.4413195999999999
0.6594044100000005	0.1590144299999992	0.4393805899999990
0.6614656699999983	0.6623900499999991	0.4396490600000007
0.4877819800000012	0.1671732100000014	0.3594826599999976
0.4864000299999987	0.6662941000000018	0.3602014299999965
0.9878201100000013	0.1672913399999985	0.3596055200000023
0.9866630100000009	0.6663886999999988	0.3601568500000027
0.1784842599999976	0.1666666699999979	0.1198975699999991
0.1784842599999976	0.6666666899999996	0.1198975699999991

0.6784842599999976	0.1666666699999979	0.1198975699999991
0.6784842599999976	0.6666666899999996	0.1198975699999991
0.3330223099999969	0.3203434200000004	0.3599843300000032
0.3331266900000003	0.8193081000000006	0.3593273700000026
0.8332283499999988	0.3202756299999976	0.3600200699999974
0.8333694100000031	0.8196328000000008	0.3592589499999974
0.3333333400000029	0.0118175899999997	0.1198975699999991
0.3333333400000029	0.5118175799999989	0.1198975699999991
0.8333334300000033	0.0118175899999997	0.1198975699999991
0.8333333699999983	0.5118175799999989	0.1198975699999991
0.1808748300000005	0.0128374800000017	0.3596684999999979
0.1786282799999981	0.5122468699999985	0.3602667299999993
0.6812065599999997	0.0129489300000003	0.3596656799999991
0.6790163800000002	0.5124051700000010	0.3602071400000000
0.4881823699999970	0.3215157400000024	0.1198975699999991
0.4881823999999995	0.8215157400000024	0.1198975699999991
0.9881824200000011	0.3215157400000024	0.1198975699999991
0.9881824200000011	0.8215157400000024	0.1198975699999991
0.3310514100000006	0.3375790600000030	0.5322773099999978
0.3318915199999992	0.8478493400000033	0.5192592800000000
0.8385023000000018	0.3360328799999976	0.5261176899999995
0.8315146099999993	0.8465593600000005	0.5194416900000007
0.0118176000000005	0.3333333400000029	0.2794877899999975
0.0118175899999997	0.8333333699999983	0.2794877899999975
0.5118175799999989	0.3333333400000029	0.2794877899999975
0.5118175799999989	0.8333333699999983	0.2794877899999975
0.1708028600000020	0.4868565399999980	0.5219824300000013
0.1527866799999984	0.9816777899999991	0.5183119000000005
0.6679546499999987	0.4885806799999983	0.5223931800000017
0.6602421699999965	0.9872020099999972	0.5185585999999986
0.1666666699999979	0.1784842599999976	0.2794877899999975
0.1666666899999996	0.6784842599999976	0.2794877899999975
0.6666666899999996	0.1784842599999976	0.2794877899999975
0.6666666899999996	0.6784842599999976	0.2794877899999975
0.0212967499999976	0.1758748800000021	0.5193099600000011
0.0144693900000021	0.6736299700000004	0.5225354199999970
0.5221282199999990	0.1734064500000017	0.5183252300000021
0.5124242400000014	0.6749259199999997	0.5240267399999965
0.3215157400000024	0.4881824200000011	0.2794877899999975
0.3215157400000024	0.9881824200000011	0.2794877899999975
0.8215157400000024	0.4881824200000011	0.2794877899999975
0.8215157400000024	0.9881824200000011	0.2794877899999975
0.4175390099999987	0.4153276400000010	0.5510730999999964
0.2334171600000019	0.3380182499999975	0.5410017339982278

9) H_2O formation on defect Fe_2O_3

DFT free energy: -828.437

Frequencies: 3502.336 cm^{-1}, 3270.260 cm^{-1}, 1522.346 cm^{-1}, 846.589 cm^{-1}, 712.785 cm^{-1}, 366.767 cm^{-1}, 307.634 cm^{-1}, 167.178 cm^{-1}, 26.845 cm^{-1}

Structure:

H_2O form

1.00000000000000

10.0776023865000006 0.0000000000000000 0.0000000000000000

-5.0388011932000003 8.7274596759000005 0.0000000000000000

0.0000000000000000 0.0000000000000000 28.7955398560000013

Fe O H

48 72 2

Selective dynamics

Direct

0.0000000000000000 0.0000000000000000 0.2495813370000022

0.0000000000000000 0.5000000000000000 0.2495813370000022

0.5000000000000000 0.0000000000000000 0.2495813370000022

0.5000000000000000 0.5000000000000000 0.2495813370000022

0.0005702361358075 0.9987818089790110 0.3908920156812741

0.9955448698262330 0.4968953455678573 0.3909278399311376

0.4978141043361859 0.0019103929086199 0.3920309623154594

0.5041278051520663 0.5020640359540423 0.3908333809923690

0.0000000000000000 0.0000000000000000 0.1499616649999993

0.0000000000000000 0.5000000000000000 0.1499616649999993

0.5000000000000000 0.0000000000000000 0.1499616649999993

0.5000000000000000 0.5000000000000000 0.1499616649999993

0.0055914153966796 0.9991803972793249 0.4899065350112650

0.0013991372766142 0.5019558472968768 0.4905958999998390

0.5019726881055391 0.0039205059611618 0.4913473515075921

0.5061546627173499 0.4985893551104965 0.4869698511964415

0.3362363166814362 0.1669347193221995 0.4100481822100193

0.3328616855584343 0.6681219672575907 0.4062117937611305

0.8268564569987973 0.1593231232560512 0.4089097599634783

0.8368251581826343 0.6680268564943930 0.4101468695193944

0.3383942955058856 0.1680014073469138 0.5431364309757143

0.3395129842317246 0.6701936243646216 0.5056000115673882

0.8420434795956595 0.1634807628301544 0.5311446892938605

0.8305969558604644 0.6615249050615333 0.5338895069732814

0.3333333429999996 0.1666666719999981 0.3096570069999984

0.3333333429999996 0.6666666870000029 0.3096570069999984

0.8333333730000021 0.1666666719999981 0.3096570069999984

0.8333333730000021 0.6666666870000029 0.3096570069999984

0.3333333429999996 0.1666666719999981 0.1697336730000032

0.3333333429999996 0.6666666870000029 0.1697336730000032

0.8333333730000021 0.1666666719999981 0.1697336730000032

0.8333333730000021 0.6666666870000029 0.1697336730000032

0.1666666719999981 0.3333333429999996 0.0898860100000007

0.1666666570000004 0.8333333730000021 0.0898860100000007

0.6666666870000029 0.3333333429999996 0.0898860100000007

0.6666666870000029 0.8333333730000021 0.0898860100000007

0.1666666719999981 0.3333333429999996 0.2298093140000006

0.1666666570000004 0.8333333730000021 0.2298093140000006

0.6666666870000029 0.3333333429999996 0.2298093140000006

0.6666666870000029 0.8333333730000021 0.2298093140000006

0.1600005568535181	0.3333876218328342	0.4667919521243036
0.1710139106336470	0.8402912877746331	0.4786840240744468
0.6663834193560376	0.3307186309402894	0.4784959571239682
0.6709701947342381	0.8299374906098222	0.4779421771256480
0.1668073448244627	0.3324694815192402	0.3298656962270030
0.1663104426822457	0.8335364547099999	0.3298599890099823
0.6663184969168299	0.3332806063041005	0.3295967526000965
0.6674885868023495	0.8334481364738267	0.3298315285555518
0.1533164379999974	0.0000000000000000	0.1997715090000014
0.1533164530000022	0.5000000000000000	0.1997715090000014
0.6533164379999974	0.0000000000000000	0.1997715090000014
0.6533164379999974	0.5000000000000000	0.1997715090000014
0.3466558810074645	0.9985807006700540	0.4407212747001950
0.3427739285225684	0.5019751782666617	0.4399438754669802
0.8461767995461429	0.0008133271326756	0.4424237036742582
0.8459728592048990	0.5001211186346950	0.4422293026643942
0.0000000000000000	0.1533164379999974	0.1997715090000014
0.0000000000000000	0.6533164379999974	0.1997715090000014
0.5000000000000000	0.1533164379999974	0.1997715090000014
0.5000000000000000	0.6533164379999974	0.1997715090000014
0.9937758838353048	0.3424562487382943	0.4387256704877913
0.0040663393100928	0.8462739196013942	0.4413710818419148
0.5005816442437521	0.3436648650462004	0.4419614202299655
0.4925090290884597	0.8296329888715377	0.4454553769086900
0.3466835919999980	0.3466835919999980	0.1997715090000014
0.3466835919999980	0.8466836209999968	0.1997715090000014
0.8466836209999968	0.3466835919999980	0.1997715090000014
0.8466836209999968	0.8466836209999968	0.1997715090000014
0.1576918701743253	0.1566303801150113	0.4402241406031777
0.1629255031560248	0.6562186788252049	0.4437847981940024
0.6533039893726027	0.1517424687936852	0.4415383930248424
0.6599137548178717	0.6610770199983804	0.4390112393009034
0.4869906010851963	0.1662527351959042	0.3601413873533161
0.4866913639298005	0.6657355133532405	0.3593716407388783
0.9856793644435342	0.1644939736466426	0.3599518048420762
0.9880944414609019	0.6659489739595230	0.3605823076810708
0.1800168899999974	0.1666666719999981	0.1199238370000018
0.1800168749999997	0.6666666870000029	0.1199238370000018
0.6800168749999997	0.1666666719999981	0.1199238370000018
0.6800168749999997	0.6666666870000029	0.1199238370000018
0.3353084820832279	0.3218403403886470	0.3602703068837201
0.3347079625370384	0.8216820297835952	0.3600331611039422
0.8317622771050495	0.3185981805129288	0.3597070239519056
0.8357295105242528	0.8213787791228171	0.3596904916385810
0.3333333429999996	0.0133502309999969	0.1199238370000018
0.3333333429999996	0.5133502480000018	0.1199238370000018
0.8333333730000021	0.0133502309999969	0.1199238370000018
0.8333333730000021	0.5133502480000018	0.1199238370000018
0.1803241504176754	0.0137235533933620	0.3599577170882355
0.1769357431686274	0.5125086523681119	0.3597054712733581

0.6761630021961551 0.0100141687598096 0.3610002541983803

0.6811429377748013 0.5133650608989910 0.3600508623505547

0.4866497519999982 0.3199830949999978 0.1199238370000018

0.4866497519999982 0.8199831250000003 0.1199238370000018

0.9866496920000003 0.3199830949999978 0.1199238370000018

0.9866497519999982 0.8199831250000003 0.1199238370000018

0.3278637966438467 0.8399526724241611 0.5216596447932247

0.8318598354348339 0.3347186995332123 0.5212864945350262

0.8298037478512441 0.8372948381977210 0.5218673250329502

0.0133502199999995 0.3333333429999996 0.2796191569999991

0.0133502520000022 0.8333333730000021 0.2796191569999991

0.5133502480000018 0.3333333429999996 0.2796191569999991

0.5133502480000018 0.8333333730000021 0.2796191569999991

0.1683582590872419 0.4798899977547606 0.5183602851355289

0.1665654773826972 0.9929339799835617 0.5215982255290399

0.6596324382163559 0.4793517580618030 0.5247664581381883

0.6656540787502152 0.9870209301836468 0.5214572945893039

0.1666666719999981 0.1800168899999974 0.2796191569999991

0.1666666570000004 0.6800168749999997 0.2796191569999991

0.6666666870000029 0.1800168899999974 0.2796191569999991

0.6666666870000029 0.6800168749999997 0.2796191569999991

0.0286599291158751 0.1781769985583566 0.5229507188724014

0.0120195408810702 0.6721607817075963 0.5225054214160494

0.5153139383138026 0.1754302770985632 0.5224143126316392

0.5087343125596036 0.6596015847352490 0.5236345211585842

0.3199830949999978 0.4866497809999970 0.2796191569999991

0.3199830949999978 0.9866497519999982 0.2796191569999991

0.8199831250000003 0.4866497809999970 0.2796191569999991

0.8199831250000003 0.9866497519999982 0.2796191569999991

0.3126492709879116 0.3437297512082296 0.5717363471263610

0.3944221401607990 0.4519546495945477 0.5694886237776018

0.2192136489700474 0.3417479863681408 0.5588035249864762

10) O-vacancy on the surface Fe_2O_3

DFT free energy: -812.652

Frequencies: -

Structure:

O-vac on Fe_2O_3

1.00000000000000

10.0776023865000006 0.0000000000000000 0.0000000000000000

-5.0388011932000003 8.7274596759000005 0.0000000000000000

0.0000000000000000 0.0000000000000000 28.7955398560000013

Fe O

48 71

Selective dynamics

Direct

0.0000000000000000 0.0000000000000000 0.2495813370000022

0.0000000000000000 0.5000000000000000 0.2495813370000022

0.5000000000000000	0.0000000000000000	0.2495813370000022
0.5000000000000000	0.5000000000000000	0.2495813370000022
-0.0015278065026105	-0.0025275906131902	0.3909544247047242
-0.0010402262057070	0.4999097804740393	0.3905430623902528
0.5002374264555652	-0.0002702214758023	0.3914290414307383
0.5000165151920385	0.5028961515119830	0.3900596855988233
0.0000000000000000	0.0000000000000000	0.1499616649999993
0.0000000000000000	0.5000000000000000	0.1499616649999993
0.5000000000000000	0.0000000000000000	0.1499616649999993
0.5000000000000000	0.5000000000000000	0.1499616649999993
-0.0007819994090132	-0.0045222987402533	0.4897838676356795
0.0015723093971142	0.5032410915149033	0.4889824825006509
0.5022973173234083	0.0010642203336386	0.4898033153262239
0.5027026566413836	0.5028425683934800	0.4874544018075303
0.3349313781948964	0.1643342268143094	0.4085277333371002
0.3325888170842873	0.6699472992906161	0.4093337962314258
0.8308014384426821	0.1645188104841494	0.4088044381631592
0.8335531015513113	0.6658037979403829	0.4096068520028741
0.3373542794746832	0.1784928459146704	0.5141400637898805
0.3329856527100288	0.6663162495471201	0.5331871002970997
0.8420094746124394	0.1664803070738569	0.5312349371892320
0.8275526622957339	0.6601532104594087	0.5328486261266646
0.3333333429999996	0.1666666719999981	0.3096570069999984
0.3333333429999996	0.6666666870000029	0.3096570069999984
0.8333333730000021	0.1666666719999981	0.3096570069999984
0.8333333730000021	0.6666666870000029	0.3096570069999984
0.3333333429999996	0.1666666719999981	0.1697336730000032
0.3333333429999996	0.6666666870000029	0.1697336730000032
0.8333333730000021	0.1666666719999981	0.1697336730000032
0.8333333730000021	0.6666666870000029	0.1697336730000032
0.1666666719999981	0.3333333429999996	0.0898860100000007
0.1666666570000004	0.8333333730000021	0.0898860100000007
0.6666666870000029	0.3333333429999996	0.0898860100000007
0.6666666870000029	0.8333333730000021	0.0898860100000007
0.1666666719999981	0.3333333429999996	0.2298093140000006
0.1666666570000004	0.8333333730000021	0.2298093140000006
0.6666666870000029	0.3333333429999996	0.2298093140000006
0.6666666870000029	0.8333333730000021	0.2298093140000006
0.1658964283547613	0.3368065774261092	0.4701879320233737
0.1670502754477817	0.8354311427759857	0.4771132265111167
0.6683973326592008	0.3299598163774989	0.4782765391470243
0.6668702750883040	0.8329441300300873	0.4756805611246361
0.1666968803603526	0.3332017180909198	0.3299112326371575
0.1664281045845964	0.8331404457434612	0.3298446222794623
0.6665838127053005	0.3332808117595503	0.3297547255289063
0.6669501167747465	0.8337237503999702	0.3296073319546691
0.1533164379999974	0.0000000000000000	0.1997715090000014
0.1533164530000022	0.5000000000000000	0.1997715090000014
0.6533164379999974	0.0000000000000000	0.1997715090000014
0.6533164379999974	0.5000000000000000	0.1997715090000014

0.3448639904341602	0.9969725855721996	0.4403901614520770
0.3466476288370633	0.5013853826800682	0.4401055518233735
0.8467610653767735	1.0014173283903420	0.4413430422247073
0.8417404470755083	0.4999909556857758	0.4421912871470651
0.0000000000000000	0.1533164379999974	0.1997715090000014
0.0000000000000000	0.6533164379999974	0.1997715090000014
0.5000000000000000	0.1533164379999974	0.1997715090000014
0.5000000000000000	0.6533164379999974	0.1997715090000014
-0.0036126516640357	0.3457194455968060	0.4395271897495626
0.0006973262495239	0.8448596015597029	0.4405158586717136
0.4915703345053001	0.3366744504072025	0.4454267469165432
0.5015890380573238	0.8438870796786049	0.4410561173601382
0.3466835919999980	0.3466835919999980	0.1997715090000014
0.3466835919999980	0.8466836209999968	0.1997715090000014
0.8466836209999968	0.3466835919999980	0.1997715090000014
0.8466836209999968	0.8466836209999968	0.1997715090000014
0.1628998406915294	0.1585449779663076	0.4421922965485668
0.1542788819448255	0.6555853594220693	0.4411809233632288
0.6559846407162183	0.1554695410377231	0.4408271594859046
0.6558995353396925	0.6588870888174472	0.4390152193959209
0.4871821413438079	0.1664737940174609	0.3602780610072264
0.4860690919531926	0.6666223485007499	0.3594398286127762
0.9876039520092432	0.1656448136938911	0.3603417608728619
0.9858746715759404	0.6656435307572307	0.3599184853120230
0.1800168899999974	0.1666666719999981	0.1199238370000018
0.1800168749999997	0.6666666870000029	0.1199238370000018
0.6800168749999997	0.1666666719999981	0.1199238370000018
0.6800168749999997	0.6666666870000029	0.1199238370000018
0.3348977790198226	0.3210750082157469	0.3601078784065452
0.3339405267039732	0.8207489596027852	0.3599988796015777
0.8335726062113366	0.3204690009927084	0.3596493001612213
0.8341937727770464	0.8206905708177522	0.3596044004642298
0.3333333429999996	0.0133502309999969	0.1199238370000018
0.3333333429999996	0.5133502480000018	0.1199238370000018
0.8333333730000021	0.0133502309999969	0.1199238370000018
0.8333333730000021	0.5133502480000018	0.1199238370000018
0.1791135588050468	0.0132683040912358	0.3599854626149785
0.1790943797229959	0.5134255575053623	0.3600462066116144
0.6794761970828573	0.0131229948472060	0.3599577745079158
0.6780952694784717	0.5116778150068989	0.3604924893079895
0.4866497519999982	0.3199830949999978	0.1199238370000018
0.4866497519999982	0.8199831250000003	0.1199238370000018
0.9866496920000003	0.3199830949999978	0.1199238370000018
0.9866497519999982	0.8199831250000003	0.1199238370000018
0.3274439051896065	0.8380651775937517	0.5209595119563188
0.8322887540211417	0.3383702337198110	0.5214976069723051
0.8238815040096176	0.8338458897378881	0.5208354519216526
0.0133502199999995	0.3333333429999996	0.2796191569999991
0.0133502520000022	0.8333333730000021	0.2796191569999991
0.5133502480000018	0.3333333429999996	0.2796191569999991

0.5133502480000018 0.8333333730000021 0.2796191569999991

0.1640820216188145 0.4848060674224505 0.5204129467052948

0.1610327080482209 0.9875008958092986 0.5201702129294301

0.6511445312963882 0.4809656500244344 0.5238182085717954

0.6663604755230580 0.9881956630557817 0.5207321769531128

0.1666666719999981 0.1800168899999974 0.2796191569999991

0.1666666570000004 0.6800168749999997 0.2796191569999991

0.6666666870000029 0.1800168899999974 0.2796191569999991

0.6666666870000029 0.6800168749999997 0.2796191569999991

0.0250206428776447 0.1761883160839835 0.5220956446607237

0.0097059837844925 0.6715582586867448 0.5220624014308203

0.5185534946655140 0.1739814252374183 0.5213257868883796

0.5090990444341533 0.6671955831008576 0.5222908908194686

0.3199830949999978 0.4866497809999970 0.2796191569999991

0.3199830949999978 0.9866497519999982 0.2796191569999991

0.8199831250000003 0.4866497809999970 0.2796191569999991

0.8199831250000003 0.9866497519999982 0.2796191569999991

Table S18: Gibbs free energy calculation at 400 °C.

No	State	DFT free energy [eV]	ZPE [eV]	U [eV]	S [meV/K]	Δ G [eV]
1	Slab	-820.506	0.086	-820.317	0.28	-820.508
2	H_2 (gas)	-6.767	0.267	-6.296	1.60	-7.371
3	H_2 on Fe	-827.487	0.446	826.737	0.95	-827.380
4	H_2 split (TS)	-827.121	0.420	-826.514	0.48	-826.836
5	H on Fe and H on O	-827.762	0.539	-826.981	0.65	-827.418
6	H-migration (TS)	-826.599	0.487	-825.900	0.57	-826.282
7	2H on 2O	-828.820	0.682	-827.913	0.60	-828.320
8	2H on 1O (TS)	-827.396	0.664	-826.578	0.48	-826.898
9	H_2O formation	-828.437	0.666	827.535	0.81	-828.078
10	O-vacancy on surface	-812.652	0.000	-812.652	0.00	-812.652

Absorption of hydrogen and water in a Fe_2O_3 interstitial site (IS)

1) Fe_2O_3 (bulk)

DFT free energy: -839.745 eV

Frequencies: 547.588 cm^{-1}, 449.322 cm^{-1}, 345.621 cm^{-1}

Structure:

```
alpha-Fe2O3
   1.00000000000000
    10.0776023865000006    0.0000000000000000    0.0000000000000000
    -5.0388011932000003    8.7274596759000005    0.0000000000000000
     0.0000000000000000    0.0000000000000000   13.7955408096000003
   Fe   O
    48    72
Direct
  0.0000000000000000  0.0000000000000000  0.3539679940000013
  0.0000000000000000  0.5000000000000000  0.3539679940000013
  0.5000000000000000  0.0000000000000000  0.3539679940000013
  0.5000000000000000  0.5000000000000000  0.3539679940000013
  0.0000000000000000  0.0000000000000000  0.6460310220000025
  0.0000000000000000  0.5000000000000000  0.6460310220000025
  0.5000000000000000  0.0000000000000000  0.6460310220000025
  0.5000000000000000  0.5000000000000000  0.6460310220000025
  0.0000000000000000  0.0000000000000000  0.1460320059999987
  0.0000000000000000  0.5000000000000000  0.1460320059999987
  0.5000000000000000  0.0000000000000000  0.1460320059999987
  0.5000000000000000  0.5000000000000000  0.1460320059999987
  0.0000000000000000  0.0000000000000000  0.8539689779999975
  0.0000000000000000  0.5000000000000000  0.8539689779999975
  0.5000000000000000  0.0000000000000000  0.8539689779999975
  0.5000000000000000  0.5000000000000000  0.8539689779999975
  0.3333329860000021  0.1666669990000003  0.6873019929999984
  0.3333329860000021  0.6666669849999991  0.6873019929999984
  0.8333330150000009  0.1666669990000003  0.6873019929999984
  0.8333330150000009  0.6666669849999991  0.6873019929999984
  0.3333329860000021  0.1666669990000003  0.9793649909999971
  0.3333329860000021  0.6666669849999991  0.9793649909999971
  0.8333330150000009  0.1666669990000003  0.9793649909999971
  0.8333330150000009  0.6666669849999991  0.9793649909999971
  0.3333329860000021  0.1666669990000003  0.4793649909999971
  0.3333329860000021  0.6666669849999991  0.4793649909999971
  0.8333330150000009  0.1666669990000003  0.4793649909999971
  0.8333330150000009  0.6666669849999991  0.4793649909999971
  0.3333329860000021  0.1666669990000003  0.1873019929999984
  0.3333329860000021  0.6666669849999991  0.1873019929999984
  0.8333330150000009  0.1666669990000003  0.1873019929999984
  0.8333330150000009  0.6666669849999991  0.1873019929999984
  0.1666669990000003  0.3333329860000021  0.0206349990000021
  0.1666669990000003  0.8333330150000009  0.0206349990000021
  0.6666669849999991  0.3333329860000021  0.0206349990000021
  0.6666669849999991  0.8333330150000009  0.0206349990000021
  0.1666669990000003  0.3333329860000021  0.3126980070000016
  0.1666669990000003  0.8333330150000009  0.3126980070000016
  0.6666669849999991  0.3333329860000021  0.3126980070000016
  0.6666669849999991  0.8333330150000009  0.3126980070000016
  0.1666669990000003  0.3333329860000021  0.8126980070000016
```

0.1666669990000003	0.8333330150000009	0.8126980070000016
0.6666669849999991	0.3333329860000021	0.8126980070000016
0.6666669849999991	0.8333330150000009	0.8126980070000016
0.1666669990000003	0.3333329860000021	0.5206350090000029
0.1666669990000003	0.8333330150000009	0.5206350090000029
0.6666669849999991	0.3333329860000021	0.5206350090000029
0.6666669849999991	0.8333330150000009	0.5206350090000029
0.1533160059999972	0.0000000000000000	0.2500000000000000
0.1533160059999972	0.5000000000000000	0.2500000000000000
0.6533160210000020	0.0000000000000000	0.2500000000000000
0.6533160210000020	0.5000000000000000	0.2500000000000000
0.3466840090000005	0.0000000000000000	0.7500000000000000
0.3466840090000005	0.5000000000000000	0.7500000000000000
0.8466839789999980	0.0000000000000000	0.7500000000000000
0.8466839789999980	0.5000000000000000	0.7500000000000000
0.0000000000000000	0.1533160059999972	0.2500000000000000
0.0000000000000000	0.6533160210000020	0.2500000000000000
0.5000000000000000	0.1533160059999972	0.2500000000000000
0.5000000000000000	0.6533160210000020	0.2500000000000000
0.0000000000000000	0.3466840090000005	0.7500000000000000
0.0000000000000000	0.8466839789999980	0.7500000000000000
0.5000000000000000	0.3466840090000005	0.7500000000000000
0.5000000000000000	0.8466839789999980	0.7500000000000000
0.3466840090000005	0.3466840090000005	0.2500000000000000
0.3466840090000005	0.8466839789999980	0.2500000000000000
0.8466839789999980	0.3466840090000005	0.2500000000000000
0.8466839789999980	0.8466839789999980	0.2500000000000000
0.1533160059999972	0.1533160059999972	0.7500000000000000
0.1533160059999972	0.6533160210000020	0.7500000000000000
0.6533160210000020	0.1533160059999972	0.7500000000000000
0.6533160210000020	0.6533160210000020	0.7500000000000000
0.4866499899999965	0.1666669990000003	0.5833330150000009
0.4866499899999965	0.6666669849999991	0.5833330150000009
0.9866499899999965	0.1666669990000003	0.5833330150000009
0.9866499899999965	0.6666669849999991	0.5833330150000009
0.1800169939999989	0.1666669990000003	0.0833330009999997
0.1800169939999989	0.6666669849999991	0.0833330009999997
0.6800169939999989	0.1666669990000003	0.0833330009999997
0.6800169939999989	0.6666669849999991	0.0833330009999997
0.3333329860000021	0.3199830060000011	0.5833330150000009
0.3333329860000021	0.8199830060000011	0.5833330150000009
0.8333330150000009	0.3199830060000011	0.5833330150000009
0.8333330150000009	0.8199830060000011	0.5833330150000009
0.3333329860000021	0.0133500000000026	0.0833330009999997
0.3333329860000021	0.5133500100000035	0.0833330009999997
0.8333330150000009	0.0133500000000026	0.0833330009999997
0.8333330150000009	0.5133500100000035	0.0833330009999997
0.1800169939999989	0.0133500000000026	0.5833330150000009
0.1800169939999989	0.5133500100000035	0.5833330150000009
0.6800169939999989	0.0133500000000026	0.5833330150000009

0.6800169939999989 0.5133500100000035 0.5833330150000009

0.4866499899999965 0.3199830060000011 0.0833330009999997

0.4866499899999965 0.8199830060000011 0.0833330009999997

0.9866499899999965 0.3199830060000011 0.0833330009999997

0.9866499899999965 0.8199830060000011 0.0833330009999997

0.3199830060000011 0.3333329860000021 0.9166669849999991

0.3199830060000011 0.8333330150000009 0.9166669849999991

0.8199830060000011 0.3333329860000021 0.9166669849999991

0.8199830060000011 0.8333330150000009 0.9166669849999991

0.0133500000000026 0.3333329860000021 0.4166670139999979

0.0133500000000026 0.8333330150000009 0.4166670139999979

0.5133500100000035 0.3333329860000021 0.4166670139999979

0.5133500100000035 0.8333330150000009 0.4166670139999979

0.1666669990000003 0.4866499899999965 0.9166669849999991

0.1666669990000003 0.9866499899999965 0.9166669849999991

0.6666669849999991 0.4866499899999965 0.9166669849999991

0.6666669849999991 0.9866499899999965 0.9166669849999991

0.1666669990000003 0.1800169939999989 0.4166670139999979

0.1666669990000003 0.6800169939999989 0.4166670139999979

0.6666669849999991 0.1800169939999989 0.4166670139999979

0.6666669849999991 0.6800169939999989 0.4166670139999979

0.0133500000000026 0.1800169939999989 0.9166669849999991

0.0133500000000026 0.6800169939999989 0.9166669849999991

0.5133500100000035 0.1800169939999989 0.9166669849999991

0.5133500100000035 0.6800169939999989 0.9166669849999991

0.3199830060000011 0.4866499899999965 0.4166670139999979

0.3199830060000011 0.9866499899999965 0.4166670139999979

0.8199830060000011 0.4866499899999965 0.4166670139999979

0.8199830060000011 0.9866499899999965 0.4166670139999979

2) H_2O (molecule, ideal gas)

DFT free energy: -14.217 eV

Frequencies: 3836.931 cm^{-1}, 3744. 719 cm^{-1}, 1588,542 cm^{-1}

Structure:

H₂O_molecule

1.00000000000000

20.0000000000000000 0.0000000000000000 0.0000000000000000

0.0000000000000000 20.0000000000000000 0.0000000000000000

0.0000000000000000 0.0000000000000000 20.0000000000000000

H O

2 1

Direct

0.5465866165038946 0.5384438543789969 0.5000000000000000

0.5465866165038946 0.4615561456210031 0.5000000000000000

0.5168267669922173 0.5000000000000000 0.4999500000000001

3) H_2 in IS of Fe_2O_3

DFT free energy: -844.746 eV

Frequencies: 4127.648 cm^{-1}, 1022.032 cm^{-1}, 950.558 cm^{-1}, 870.045 cm^{-1}, 854.661 cm^{-1}, 521.353 cm^{-1}, 429.679 cm^{-1}, 351.805 cm^{-1}, 181.462 cm^{-1}

Structure:

```
H2 in Fe2O3
   1.00000000000000
    10.0776023865000006    0.0000000000000000    0.0000000000000000
    -5.0388011932000003    8.7274596759000005    0.0000000000000000
     0.0000000000000000    0.0000000000000000   13.7955408096000003
   Fe   O    H
    48    72     2
Selective dynamics
Direct
  0.0006795741729988  0.0010813348877363  0.3535878276258870
  0.9991976846355399  0.4996091272758250  0.3536611781730770
  0.5001921350873957  0.9994025749539475  0.3537010738599164
  0.5009146242756302  0.5003773730353061  0.3523030805237397
  0.9993204258270012  0.9989186651122637  0.6464111883741168
  0.0008023153644601  0.5003908727241750  0.6463378378269198
  0.4998078649126043  0.0005974250460525  0.6462979421400874
  0.4990853757243698  0.4996226269647011  0.6476959354762641
  0.0000621498957187  0.0000912618996551  0.1457734533888484
  0.9999660166018245  0.4999736168997799  0.1458400794056232
  0.5000373216552703  0.9999633056347150  0.1458127801160245
  0.4999839835332409  0.5000132359449907  0.1455004511015119
  0.9999378501042813  0.9999087381003449  0.8542275306111407
  0.0000339833981755  0.5000263831002201  0.8541609045943730
  0.4999626783447297  0.0000366943652850  0.8541882038839717
  0.5000160164667520  0.4999867640550093  0.8545005328984843
  0.3318868728380195  0.1659936481935560  0.6878925500525028
  0.3339600851170488  0.6680396103658452  0.6879398453171106
  0.8332393690970079  0.1665132354234231  0.6875601195555348
  0.8337422929019596  0.6659779642220442  0.6877327845757435
  0.3333328083400886  0.1665764103175107  0.9794385363086278
  0.3332453313644592  0.6666636146824132  0.9794448978341421
  0.8333014977308792  0.1666758650178295  0.9794303918891529
  0.8334265527245677  0.6667454142482470  0.9794227260756898
  0.3328265077248957  0.1652594607838296  0.4796156720733435
  0.3315419624346774  0.6685098452309859  0.4796666744917246
  0.8331495501021990  0.1660282026385076  0.4793549468990790
  0.8367861601487974  0.6685976931461965  0.4792838860272539
  0.3334561605521245  0.1667135893393166  0.1871527526338070
  0.3332585222403992  0.6665818186239676  0.1871890488090315
  0.8333527579864679  0.1666456747311855  0.1870818749540319
  0.8334998774973030  0.6668695797972930  0.1870890080980558
  0.1665734612754335  0.3332545567517542  0.0205772639243094
  0.1666985162691219  0.8333241489821717  0.0205695981108462
  0.6667546396355419  0.3333363563175880  0.0205550921658570
  0.6666671626599125  0.8334236036824905  0.0205614536913714
```

0.1662577210980416	0.3340220067779569	0.3122672154242565
0.1667606449029932	0.8334867785765780	0.3124398804444652
0.6660398858829524	0.3319603606341559	0.3120601546828894
0.6681130981619816	0.8340063658064452	0.3121074499474972
0.1665001365026981	0.3331303912027082	0.8129109919019442
0.1666472560135333	0.8333543392688156	0.8129181250459681
0.6667414487596020	0.3334181523760336	0.8128109511909685
0.6665438104478767	0.8332864246606846	0.8128472473661930
0.1632138538512038	0.3314022778538046	0.5207161139727461
0.1668504638978021	0.8339718113614936	0.5206450531009210
0.6684580085653238	0.3314901257690153	0.5203333255082754
0.6671734632751054	0.8347405532161716	0.5203843279266565
0.1537875568340539	0.0004889752346102	0.2499442306296871
0.1522610398077262	0.4999114190665566	0.2490948077814608
0.6534181853663270	0.9999862271580966	0.2497533847483950
0.6531905095646522	0.4998893791756700	0.2497253479709300
0.3465818446336755	0.0000137728419034	0.7502466152516050
0.3468095204353503	0.5001106208243300	0.7502746520290700
0.8462124281659413	0.9995110247653898	0.7500557693703129
0.8477389451922690	0.5000885809334434	0.7509051922185463
0.0001509335717174	0.1535662327181555	0.2497692401236833
0.9996894797525044	0.6533886040402663	0.2499621597766222
0.5001259412695944	0.1521463840553707	0.2488958179368836
0.5002100535004175	0.6531976500513395	0.2498505211646815
0.0003105202474956	0.3466114259597362	0.7500378402233778
0.9998490664282826	0.8464337522818397	0.7502307598763167
0.4997899464995825	0.3468023799486630	0.7501494788353185
0.4998740587304056	0.8478536009446245	0.7511041820631164
0.3469352849837151	0.3470490938327657	0.2499055939055665
0.3466829989739750	0.8462769191443940	0.2500099799963138
0.8466945981472165	0.3466165181687515	0.2497427089588200
0.8479617870461311	0.8480509186795047	0.2488282791455489
0.1520381979538641	0.1519490663204905	0.7511717208544511
0.1533053868527787	0.6533835118312510	0.7502572910411800
0.6533170310260203	0.1537230658556012	0.7499900200036862
0.6530647450162945	0.6529509361672368	0.7500944060944335
0.4863424245636878	0.1671404627996367	0.5831736833920900
0.4875503241114956	0.6744760320691299	0.5874175653801217
0.9851740186602314	0.1651192563958048	0.5836520771297273
0.9882487210875226	0.6673132389722767	0.5834912450271261
0.1802761863458002	0.1668678542164415	0.0833496786524250
0.1800936866478935	0.6666938589538560	0.0832460161771564
0.6800999852946106	0.1666364462247643	0.0831246000526207
0.6800396244409939	0.6667099451938228	0.0832204769329437
0.3246499955090343	0.3131401668929783	0.5874526769543564
0.3326495017696516	0.8211899471962454	0.5836106358303610
0.8345825191874354	0.3198257164469211	0.5836123292386048
0.8328135920948583	0.8189690885502685	0.5831451481925853
0.3332902614810180	0.0133657809981855	0.0832112645627987
0.3332741950934661	0.5133248789392582	0.0832460479517465

0.8333876625814369	0.0134908223435488	0.0831228857400177
0.8332014454563108	0.5134123170291076	0.0833465896241421
0.1789072573581052	0.0118749790377137	0.5836469225870786
0.1805524640952356	0.5136916803018252	0.5832172773455966
0.6798291907168164	0.0140150272283321	0.5834686795557360
0.6860793809922470	0.5114641800048574	0.5867041684697014
0.4866780977126908	0.3199720726836830	0.0832358773012558
0.4865802135376995	0.8197278587869263	0.0833610878479902
0.9865497373584304	0.3199360243499143	0.0831440840020363
0.9866545721808535	0.8199273296912537	0.0831836427217993
0.3199603755590061	0.3332900258061784	0.9167795090670552
0.3199000147053894	0.8333635677752369	0.9168753859473782
0.8199063133521065	0.3333061120461451	0.9167539698228424
0.8197238136541998	0.8331321597835597	0.9166503073475738
0.0117512689124766	0.3326867320277245	0.4165087839728727
0.0148259713397678	0.8348807576041963	0.4163479518702715
0.5124496758885044	0.3255239389308713	0.4125824636198772
0.5136575754363122	0.8328595512003645	0.4168263456079089
0.1667985685436904	0.4865876829708924	0.9166533963758567
0.1666123514185642	0.9865091676564504	0.9168771002599811
0.6667257759065350	0.4866751210607418	0.9167539380482523
0.6667097095189831	0.9866342090018136	0.9167887214372001
0.1671864219051429	0.1810309114497315	0.4168548808074135
0.1654174948125657	0.6801742835530789	0.4163876997613940
0.6673504692303496	0.1788100528037546	0.4163893931696379
0.6753499754909669	0.6868598331070217	0.4125473520456424
0.0133454178191457	0.1800726703087463	0.9168163432781995
0.0134502526415687	0.6800639756500857	0.9168559019979625
0.5134197864623005	0.1802721412130737	0.9166388981520086
0.5133219022873092	0.6800279273163170	0.9167641086987430
0.3139206190077530	0.4885358199951426	0.4132958605302974
0.3201708092831836	0.9859849627716670	0.4165313494442628
0.8194475359047644	0.4863083196981748	0.4167827516544023
0.8210927426418948	0.9881250109622854	0.4163531064129202
0.4625033853966514	0.4994641296699598	0.4987981832345696
0.5374966436033475	0.5005358703300403	0.5004769448566002

4) 2H split in IS

DFT free energy: -845.660 eV

Frequencies: 3319.991 cm^{-1}, 1479.245 cm^{-1}, 1327.636 cm^{-1}, 893.829 cm^{-1}, 670.004 cm^{-1}, 523.721 cm^{-1},479.367 cm^{-1}, 393.607 cm^{-1}

Structure:

2H attached in Fe_2O_3

1.00000000000000

10.0776023865000006	0.0000000000000000	0.0000000000000000
-5.0388011932000003	8.7274596759000005	0.0000000000000000
0.0000000000000000	0.0000000000000000	13.7955408096000003

Fe O H

48 72 2

Selective dynamics

Direct

0.9965992065705365 0.9972124712368000 0.3531308680069571

0.9974700632023143 0.4971889828491314 0.3531887724853249

0.5015483606974414 0.0002556564488501 0.3515390654082182

0.5138041646157774 0.5021495178272275 0.3431580499895261

0.9969071764889819 0.9988374504216893 0.6492675475566401

0.9991552872505665 0.5000899357805366 0.6459850361699750

0.5012429415538264 0.9991221152895164 0.6475989523224541

0.5086297754957485 0.5108457400262907 0.6532155124555032

0.0001351929988687 0.9995669138070440 0.1454909869975651

0.0003750142203671 0.4991728488909857 0.1459434393401366

0.4993061224414319 0.9985237628506667 0.1454747583070173

0.5016162098307149 0.5007704555702901 0.1442361789606252

0.0010307059421848 0.0005200923535398 0.8549404482472625

0.9999768111958005 0.4993391087209389 0.8541173914652589

0.4999941299367734 0.9999415271042835 0.8549882112748506

0.4998302202080538 0.4993053576101403 0.8563011882081497

0.3354791746188255 0.1638032612810392 0.6876272138105861

0.3317994760923426 0.6660341399674294 0.6878440819809200

0.8328213481236872 0.1675371471280940 0.6881481412355583

0.8368979849651268 0.6674708847453843 0.6873367633323113

0.3337172616097064 0.1664594161086796 0.9799256142889448

0.3333300054789632 0.6661929155499919 0.9793130877897909

0.8337447725792941 0.1667268722068300 0.9793207975247924

0.8342653814427621 0.6668429697677851 0.9785294070581969

0.3121760913594969 0.1382111222807438 0.4769726228322639

0.3307374269748991 0.6828583952873402 0.4821254652248257

0.8273789406948211 0.1643369112838329 0.4792748281404613

0.8322373058208683 0.6653778816048330 0.4798690927500999

0.3332917597169924 0.1661066741591597 0.1875686370726939

0.3335940512227467 0.6648703274317427 0.1865291789067740

0.8316393163376077 0.1660357341303325 0.1863636603880821

0.8363035409633284 0.6676805245759638 0.1850521287114759

0.1666048702581904 0.3327207554479870 0.0205681310724657

0.1675347374552913 0.8328195954374564 0.0208183594744966

0.6672860695474654 0.3333633966909986 0.0208663844579107

0.6670651013655373 0.8334461984252286 0.0206841366134398

0.1638890628078258 0.3262198565892760 0.3099308601883877

0.1670087610149196 0.8305155416189010 0.3114150076441149

0.6670648612331078 0.3361271221893389 0.3135946682130921

0.6653484042335549 0.8314845885007927 0.3119144876217703

0.1670429030455480 0.3336188754583915 0.8128757613673869

0.1664185508717395 0.8326650754703522 0.8137235418936299

0.6664979762756502 0.3332318557890659 0.8131070847785011

0.6672628923200250 0.8346839396007510 0.8138831163573741

0.1525823994388489 0.3278371761498065 0.5203483580328907

0.1642396784598006 0.8390973536750224 0.5203644658338717

0.6626343304157345 0.3299554477715247 0.5228912623078585

0.6677883234570672	0.8326554764107073	0.5192304963786398
0.1530477131373118	0.9991671982175490	0.2502914880738913
0.1552467079109121	0.4975924909116216	0.2513643778047268
0.6531306553348131	0.0001830894572308	0.2492018758212282
0.6597729687922680	0.4962433234358912	0.2419779304124035
0.3471271368421185	0.9997048290105397	0.7521204578978455
0.3476493637288556	0.5004984173182621	0.7502759321315438
0.8472037660024938	0.0013590033082238	0.7514154004016760
0.8469846637295007	0.4996024846940728	0.7506531554527527
0.9982613179671773	0.1516147639243073	0.2480905686525006
0.0003607469285356	0.6512732608570033	0.2490191644930562
0.4992231820157613	0.1549731955117082	0.2520963835873218
0.5048231953296138	0.6589300038991723	0.2436688071507618
0.0001579853440674	0.3467136622942775	0.7503573018587346
0.9999274776003517	0.8472384263632406	0.7519601347729434
0.4994262187647678	0.3468741673052520	0.7502270641102271
0.4997901056792955	0.8459051019150863	0.7506211845964614
0.3456133351530610	0.3454669610651777	0.2488611295457588
0.3467629415401632	0.8453882017489605	0.2472573498543227
0.8463838136762902	0.3451618684978328	0.2504211480167129
0.8470085256855526	0.8462516643355187	0.2490796050310209
0.1568103128666252	0.1572855865778777	0.7485611661302727
0.1529016147010367	0.6531679962549006	0.7504528389965799
0.6533576035777955	0.1529224815153469	0.7511000114651694
0.6564900329152792	0.6555217555032016	0.7522272740218696
0.4837803306806592	0.1626904851106445	0.5865623423545543
0.4870510342866723	0.6685483387014841	0.5833671755581520
0.9846245638581550	0.1651856093590709	0.5852484180501776
0.9854545824664740	0.6656899539366066	0.5833917364778074
0.1796740382428155	0.1659257512843695	0.0829482513365605
0.1799192630186184	0.6652918712833582	0.0829113517506528
0.6797797982005278	0.1664785668371564	0.0833785556536100
0.6817213775729272	0.6666055329995402	0.0811410319123667
0.3523795057803838	0.3421042444209093	0.5706198459907554
0.3311842864811894	0.8210439986487046	0.5853808841142865
0.8324833191061174	0.3188725081310579	0.5838176382078544
0.8330972540853878	0.8202787000823264	0.5845418835798313
0.3335434892283402	0.0132642357547397	0.0833420489456174
0.3336266598530884	0.5127896005439680	0.0821959427154297
0.8334626428324725	0.0133063909342255	0.0828185209305516
0.8365834494443973	0.5135856934305210	0.0809451657031843
0.1759345389130971	0.0115815988634580	0.5899641440337433
0.1789011695593032	0.5125251474882262	0.5842941245874300
0.6799191712628598	0.0129725966226033	0.5833889883039660
0.6815362266719731	0.5146296053799588	0.5861833700676300
0.4862770242356191	0.3192242356146622	0.0823086884233817
0.4865833244703808	0.8207224720429593	0.0812915884325207
0.9866971225723375	0.3195018441545798	0.0831590729356577
0.9878720614403420	0.8202591481172377	0.0826334487892453
0.3199187820120173	0.3329329720078320	0.9169871695607199

0.3199035676232285 0.8330284869451887 0.9169255544283814

0.8199600034762682 0.3326837021937621 0.9165034825731837

0.8209489505367102 0.8343349988678028 0.9169760976879573

0.0125329313843423 0.3326589134231455 0.4172449217615437

0.0130847405257128 0.8341145405565413 0.4172898693670035

0.5063692186464905 0.3371077455476836 0.4331312344158960

0.5140572373967842 0.8342245149436991 0.4159861291279370

0.1669238399991855 0.4863460383373663 0.9163913935636074

0.1672425569122495 0.9869553633625827 0.9169115614839498

0.6671203367363248 0.4862794915764255 0.9165997270746757

0.6670339729759149 0.9868027695803079 0.9168106625088441

0.1601235859125012 0.1812843355337321 0.4157105806765458

0.1607765854645820 0.6730302952046614 0.4160351916055234

0.6635215395930487 0.1802994038026071 0.4172465524355786

0.6712662343602744 0.6847855684430399 0.4161171299189874

0.0139357784760037 0.1803352309050297 0.9165883486676094

0.0142240157054374 0.6801629381023488 0.9163866447238931

0.5137820596063634 0.1801422546997387 0.9169312844837876

0.5136985482608196 0.6800512114910262 0.9167311958662694

0.3267274241663429 0.4988782175660589 0.4220081515614424

0.3174177922614305 0.9680986379313978 0.4136018618839685

0.8165570183109168 0.4850204925215920 0.4160778787097854

0.8193716464006542 0.9885873024020668 0.4159975299053116

0.3924764474027782 0.4956180272097441 0.4741254587411206

0.4378919645270473 0.3572596894441987 0.5061827319704826

5) O-vacancy in IS

DFT free energy: -831.602 eV

Frequencies: -

Structure:

O-vacancy in Fe_2O_3

1.00000000000000

10.0776023865000006 0.0000000000000000 0.0000000000000000

-5.0388011932000003 8.7274596759000005 0.0000000000000000

0.0000000000000000 0.0000000000000000 13.7955408096000003

Fe O

48 71

Direct

-0.0004818373873170 -0.0004475501960820 0.3538796861680036

-0.0007291756450462 0.4997194240841701 0.3532319185233674

0.5018817433282565 -0.0010452259327802 0.3523279093925004

0.4989710223760706 0.5000025727693971 0.3544571656287126

-0.0001522297455596 0.0008226595601627 0.6459866063038028

0.0007572793486087 0.5006667750650964 0.6461384742901694

0.5011478756563044 0.0007970243758245 0.6456410624966997

0.5074131636786262 0.5062525282037329 0.6467935626706026

0.0003983082433116 0.0000189528648654 0.1461283913838047

-0.0002034551632542 0.4997375452544181 0.1457292692621248

0.5000556904007993	0.0000937559915730	0.1455437782541061
0.4995534927185301	0.4995287630051684	0.1464749055041923
-0.0002307556175122	-0.0001531816406923	0.8539874825667730
-0.0002438516593305	0.4990566122090744	0.8537840933896620
0.5005502201289754	-0.0009248802970713	0.8547410834064256
0.5007615479037573	0.5002839832415370	0.8527560191114752
0.3365547479702851	0.1607904582181391	0.6932018574673927
0.3338850726352088	0.6668269140463574	0.6866991277298129
0.8337329111338661	0.1669049956083829	0.6869473419070579
0.8340672966803663	0.6676699725070457	0.6881286580612880
0.3334233140965872	0.1669800563481233	0.9793497586217278
0.3333971872263520	0.6662617653957122	0.9795416104301630
0.8339863058563437	0.1667801291670602	0.9795062871449991
0.8327079188104863	0.6660828455865808	0.9795252849685355
0.3301112240297190	0.1575686962478565	0.4734651265326024
0.3327808993647953	0.6662748274111496	0.4799678562701813
0.8325987333196355	0.1669357048266814	0.4785383259387075
0.8329331188661357	0.6665050854745164	0.4797196420929380
0.3332426579034170	0.1668897282515385	0.1873172253782676
0.3332687847736521	0.6661975641693637	0.1871253735698329
0.8339581111895155	0.1667079557760969	0.1871416990314600
0.8326797241436581	0.6661268243107162	0.1871606968549976
0.1671135062814701	0.3333082562866408	0.0201920994958083
0.1666113085992008	0.8333710805907738	0.0211232267458948
0.6662686767566863	0.3329536306215558	0.0205386136161958
0.6668704401632533	0.8332740154176733	0.0209377357378757
0.1659054510962426	0.3328554213377815	0.3139109658885230
0.1661167788710247	0.8318579145739536	0.3119259015935728
0.6668977406175118	0.3334105599768217	0.3126795024332273
0.6669108366593298	0.8326334788684060	0.3128828916103376
0.1676959766239299	0.3343645363933290	0.8122088353712914
0.1647852556717437	0.8304060457389648	0.8143380916075018
0.6671488223873168	0.3333672731912370	0.8127863148319993
0.6673961606450441	0.8337816147292172	0.8134340824766348
0.1592538353213737	0.3321723505251086	0.5198724683294028
0.1655191233436956	0.8329821637195204	0.5210249685033057
0.6668192147455581	0.3343078753057245	0.5206794246962027
0.6659097056513908	0.8332425107164887	0.5205275567098361
0.1535291037184416	-0.0006332084557365	0.2498852086105904
0.1537098887518528	0.5001667202100033	0.2501759666952614
0.6537625164703867	0.0000721437386750	0.2498397845487919
0.6527049606647037	0.4994380155549377	0.2504189206848216
0.3465897883539529	-0.0037916888265527	0.7519963813032025
0.3460491739055077	0.4977487609430849	0.7470867461989842
0.8479250870758669	0.0009277896218174	0.7507324896167006
0.8471952397568775	0.5001547404901469	0.7502158445438221
0.0006265429526608	0.1526200415588743	0.2494918064657405
-0.0004720824488070	0.6528661388050568	0.2495973122912629
0.4998730702685488	0.1536525578573422	0.2502865839766318
0.4996331821406451	0.6528681768465443	0.2502493689563901

-0.0001876352583431	0.3466184957865479	0.7500090098412625
0.0027887715847038	0.8493144317218669	0.7495124408628433
0.4986437667599738	0.3436913433477133	0.7500255907235800
0.5012133555648601	0.8474243795387966	0.7497370094126041
0.3464101548815023	0.3466311727821942	0.2504381808678808
0.3467750202852014	0.8464813168754394	0.2491938952293918
0.8451341936471631	0.3466408250267681	0.2485213615586142
0.8468413499050079	0.8461756188826994	0.2501121452235361
0.1576003099349533	0.1580434924078940	0.7465056405098643
0.1535705586661217	0.6531641529309787	0.7507708602073395
0.6522980865709949	0.1531434017861984	0.7491332790127939
0.6599021852162599	0.6555171994655390	0.7557785146096931
0.4804935407355553	0.1726988127262219	0.5843854497246761
0.4844421437369573	0.6694131221435632	0.5820952542683148
0.9829025094289932	0.1634016658768763	0.5838979133374149
0.9882497773411323	0.6689994451254710	0.5841914927396717
0.1802596954277931	0.1667185021563483	0.0831864424786186
0.1798306759941398	0.6663979329190438	0.0833089788960848
0.6800594081061972	0.1664344774986756	0.0831472904057283
0.6796924783788734	0.6663133786346052	0.0835690013636996
0.3333329860000021	0.8246973888536984	0.5833330150000009
0.8338199319437541	0.3204137761005724	0.5827079793465215
0.8328460980562477	0.8199268591568201	0.5839580506534803
0.3333329860000021	0.0134860999123418	0.0833330009999997
0.3333329860000021	0.5132427884520628	0.0833330009999997
0.8335141988723336	0.0134926360316837	0.0836022657101778
0.8331518311276682	0.5133114621593526	0.0830637362898217
0.1861734432644403	0.0255382629906658	0.5822805802753257
0.1822248402630384	0.5183039934066075	0.5845707757316870
0.6784172066588632	0.0140826727843395	0.5824745372603300
0.6837644745710023	0.5138321574478831	0.5827681166625869
0.4864072885722020	0.3197918077285549	0.0834795595213809
0.4868363080058557	0.8199002719249041	0.0833570231039147
0.9869745056211221	0.3199539152557334	0.0830970006362999
0.9866075758937982	0.8197080703924783	0.0835187115942712
0.3202568601184993	0.3335540039006946	0.9162288041321182
0.3198919947148002	0.8330393415902412	0.9174730897706079
0.8198256350949912	0.3326672549776938	0.9165548397764626
0.8215327913528360	0.8348396163796030	0.9181456234413851
0.0067638357837449	0.3289480002492817	0.4108884993903046
0.0143679344290095	0.8341783452152094	0.4175337349872041
0.5090657060650473	0.3337761684729427	0.4201613734901343
0.5130954573338796	0.8329265942648525	0.4158961537926592
0.1670338168593551	0.4865689637058935	0.9164176160436091
0.1667939287314515	0.9871134715887925	0.9163804010233679
0.6660404420473389	0.4853274826062131	0.9171751785342588
0.6671390674488064	0.9866721902538587	0.9170696727087357
0.1680232322400266	0.1783805615877382	0.4166414232764181
0.1654536434351407	0.6795440389739367	0.4169300045873942
0.6638782134152951	0.1798586751371638	0.4171545731371545

0.6668546202583426 0.6801391160448891 0.4166580041587362

0.0139610603353012 0.1800660698902352 0.9162480643151764

0.0129035045296178 0.6796426422682890 0.9168272004512069

0.5131369122815584 0.1791706878258180 0.9167817763894096

0.5129561272481478 0.6797898314581471 0.9164910183047380

0.3206178410944939 0.4850335860375739 0.4195802678010138

0.3200772266460487 0.9829525218194916 0.4146706326967953

0.8187418979241322 0.4863366715459448 0.4159345243832973

0.8194717452431216 0.9862934697332636 0.4164511694561761

6) H_2O in IS

DFT free energy: -849.983 eV

Frequencies: 2850.288 cm^{-1}, 2681.840 cm^{-1}, 1614.522 cm^{-1}, 1096.403 cm^{-1}, 1072.356 cm^{-1}, 820.108 cm^{-1}, 626.320 cm^{-1}, 563.556 cm^{-1}, 491.358 cm^{-1}, 408.236 cm^{-1}, 359.781 cm^{-1}, 330.848 cm^{-1}

Structure:

H_2O in Fe_2O_3

1.00000000000000

10.0776023865000006 0.0000000000000000 0.0000000000000000

-5.0388011932000003 8.7274596759000005 0.0000000000000000

0.0000000000000000 0.0000000000000000 13.7955408096000003

Fe O H

48 73 2

Selective dynamics

Direct

0.0054983549199292 0.0074266152378115 0.3534604674195236

0.9969532880347600 0.4992557011258398 0.3521854138637650

0.5022747166874169 0.9999372454252580 0.3533094625075606

0.4988959677947236 0.5001406229031389 0.3491675277566841

0.9985518643079558 0.9984253025817367 0.6474695349414574

0.0021719035231911 0.5012439312780614 0.6460628015881795

0.5002330529973236 0.0016078746304586 0.6463726217288865

0.4918813514265281 0.4901571007129348 0.6605112741259163

0.0005644426126992 0.0007133009769404 0.1457182130529873

0.9998112261694345 0.5005106470061378 0.1445300040491446

0.5002740834389243 0.9998511689447156 0.1453864916693703

0.5005221480303845 0.5009315928296019 0.1446952887157806

0.9988554829818881 0.9985626853251546 0.8554529589967146

0.0001104458229477 0.4997805051952469 0.8538992062965534

0.4994377788998747 0.9991522512926707 0.8543655236240397

0.5001219862336654 0.5000979223589184 0.8600549824748427

0.3272147799844873 0.1612514177902469 0.6904037743527098

0.3344216395739252 0.6696671430751167 0.6866593337762410

0.8336050780587030 0.1664544225598021 0.6878190475918231

0.8329590072229749 0.6647841168023376 0.6869311561554312

0.3331803036097725 0.1654578051493871 0.9798284584174937

0.3321159895666668 0.6664494478602805 0.9797356128747481

0.8328119610024345 0.1663730832321022 0.9794756577608013

0.8341174221709053	0.6670781562138686	0.9798001726509256
0.3373699898317071	0.1665474444749009	0.4793424549297853
0.3248711884601363	0.6724160429290649	0.4747916709880116
0.8380841093146785	0.1678883325884328	0.4793436859899742
0.8420612085279444	0.6733015433725598	0.4768416302558904
0.3344588741603687	0.1677763344802869	0.1869140195359762
0.3336220173475866	0.6674099426328937	0.1869336634802039
0.8338315691696749	0.1666750980819245	0.1869687710222294
0.8338177779755753	0.6681250659517417	0.1868723319069829
0.1660100431307185	0.3328825455579647	0.0213896770827802
0.1668012551124107	0.8336606616871194	0.0204414391208445
0.6668367012862930	0.3330298698467118	0.0210302449513406
0.6667395405785186	0.8339932081283692	0.0209158079233944
0.1664213519418141	0.3392674466358940	0.3111161302703707
0.1670003038096368	0.8350603839601121	0.3117612892417156
0.6656328666302116	0.3310160437934613	0.3090800433030410
0.6710495036359845	0.8346668913179300	0.3114192269362590
0.1648577919487408	0.3317260116960909	0.8143649430885276
0.1653751281613509	0.8318795824501848	0.8131871595172626
0.6678894092377732	0.3326843557742905	0.8138019969537709
0.6659747421781219	0.8325335872595971	0.8130124129943042
0.1667844729495513	0.3325777979365583	0.5219041635881396
0.1670418400750435	0.8366329675887343	0.5213376958637213
0.6716458066771196	0.3259293377782200	0.5178082360182827
0.6721900652858963	0.8409968581797358	0.5196523810941898
0.1556937311381930	0.0032235558876152	0.2503262545864757
0.1486065623110520	0.5008338176671288	0.2450870815100643
0.6543913510678152	0.9997449793886375	0.2492833622051336
0.6533083298035933	0.5003471145980996	0.2485366058064002
0.3460408796756482	0.9976707118167170	0.7508458462073477
0.3460978337600551	0.5025044202020084	0.7544571427716846
0.8447686757390045	0.9982191455183198	0.7500115683273165
0.8475608641840608	0.4993477673904820	0.7506631600295464
0.0014579385407743	0.1555023208501822	0.2497700593773260
0.9991251695821788	0.6550035920156887	0.2495437103429055
0.5009343811182063	0.1499986672430254	0.2461148852659747
0.5025527192003736	0.6553003500228414	0.2480363134104948
0.0009831673035663	0.3465618820045577	0.7499073974678296
0.9982510050965701	0.8442338688247943	0.7504733162645536
0.4973903219343754	0.3430206405128544	0.7546847058194714
0.5000625096310500	0.8477457420747783	0.7509823614542626
0.3470380582156238	0.3480809075112603	0.2493889396749793
0.3466837836714518	0.8458442279346343	0.2503943907054662
0.8469179024021969	0.3468050346239693	0.2486387860862251
0.8511194424647996	0.8510344455591365	0.2476863922102339
0.1482318340611144	0.1474689041717951	0.7552840321202723
0.1533684904115091	0.6530048114245233	0.7497791680795487
0.6532200915562996	0.1536871652334213	0.7499025666605164
0.6526257659400798	0.6502442334775864	0.7525063317423673
0.4862999155396892	0.1685118996523229	0.5834290658361567

0.4929685960207308	0.6780528919014159	0.5896968870287722
0.9861662466454320	0.1653907444710612	0.5840119938236867
0.9882401646045764	0.6673860839819810	0.5830271048050832
0.1806990505208432	0.1670082425654442	0.0834811090766649
0.1803053256849338	0.6674960194100308	0.0825361665214572
0.6803688654788260	0.1662620152127943	0.0825946836082636
0.6805416055632918	0.6673816172711255	0.0828390225826965
0.3108257840215174	0.3057837950133191	0.6023087763656960
0.3330917644629494	0.8224438045726501	0.5831169308788304
0.8369789375136847	0.3202645593460787	0.5839199883773745
0.8330869648127006	0.8181349190418459	0.5833579147734866
0.3331023355093876	0.0131916566006325	0.0830123062549930
0.3330822158315243	0.5133876211137860	0.0830947069708188
0.8338483719159697	0.0138153141751900	0.0829103147990793
0.8329915413149962	0.5138099059001817	0.0830012932629955
0.1783635936848711	0.0116100369070224	0.5846781790686038
0.1835763587325019	0.5164022932982064	0.5822382891998075
0.6808162382581457	0.0154983931296044	0.5841863967863503
0.6846318805311711	0.5065266966438386	0.5877226454101248
0.4868596598589363	0.3201498953271482	0.0830681139419553
0.4862220311208745	0.8197367570483749	0.0830848266035318
0.9857484169124007	0.3196299341200941	0.0824729201169419
0.9870775624206161	0.8205212099601766	0.0828838234396372
0.3190547189817039	0.3324991594165070	0.9183156494235760
0.3194618595791283	0.8327132221623756	0.9166939736208732
0.8198810229762898	0.3330529470338135	0.9164623957485460
0.8186951311837447	0.8322621111252957	0.9169472201092219
0.0108065968412987	0.3338629847699650	0.4161408617036955
0.0181065496539645	0.8409590429332070	0.4162768443211249
0.5153540578102280	0.3089357447449501	0.4055487373590836
0.5156321765382046	0.8340388772630192	0.4172158680533471
0.1657365636437191	0.4858917371671438	0.9172120755284254
0.1662316240936121	0.9854680658954180	0.9173344915118946
0.6672924764162644	0.4861711514520763	0.9180718833508976
0.6665413825666064	0.9862373575518930	0.9165803255209326
0.1735451270533304	0.1884421360278594	0.4183326458567436
0.1593682227995998	0.6827289343301715	0.4154147140604465
0.6717015362346785	0.1758974316370754	0.4159698040170454
0.6856373279092125	0.6954898265060692	0.4092856923817578
0.0126142644486791	0.1795469918589916	0.9174070028235306
0.0131965678207138	0.6793998808958150	0.9166605086258244
0.5134734924924089	0.1793048309967489	0.9172071284429890
0.5131494811211468	0.6805613571068321	0.9180966559015715
0.3007450595036403	0.5019337772694925	0.4015109084166326
0.3213068050441876	0.9856220700267002	0.4168989263893081
0.8191424489793491	0.4868463789102222	0.4164642287529432
0.8254320076868567	0.9911924755710402	0.4165136783798857
0.4774265717627060	0.4795138958921044	0.4885471306800113
0.4746191438218190	0.5710664581995886	0.5176435067382812
0.5770288487677108	0.4934729305178253	0.5156194852797065

7) H_2O in IS with O-vacancy

DFT free energy: -845.341 eV

Frequencies: 2500.339 cm^{-1}, 1974.397 cm^{-1}, 1609.528 cm^{-1}, 1169.040 cm^{-1}, 1090.553 cm^{-1}, 889.746 cm^{-1}, 828.992 cm^{-1}, 341.570 cm^{-1}, 292.563 cm^{-1}

Structure:

```
H2O in Fe2O3 with vac
   1.00000000000000
    10.0776023865000006    0.0000000000000000    0.0000000000000000
    -5.0388011932000003    8.7274596759000005    0.0000000000000000
     0.0000000000000000    0.0000000000000000   13.7955408096000003
   Fe   O    H
    48    72     2
Selective dynamics
Direct
  0.0013423247286255  0.0011184016854457  0.3519195923944807
  0.9991488107966902  0.5004033996135675  0.3504397787744082
  0.5048533660515417  0.0008264871699524  0.3500975833754509
  0.5008678845719601  0.5055956580183931  0.3475504588277829
  0.0016479167094943  0.0012107749295396  0.6455281328989955
  0.0026418632992602  0.5031310676963230  0.6463123589845452
  0.5024027821180255  0.0015668934958839  0.6443868466283291
  0.5391147055287107  0.5233330900845701  0.6662647302133280
  0.0028161785643945  0.0022171670133631  0.1447533770877669
  0.0021569906357328  0.5021667567200723  0.1435793345625100
  0.5022526599497397  0.0026559240687831  0.1443586782682189
  0.5018683235524790  0.5016109643610136  0.1432004903448600
  0.0038088245301822  0.0032807057043627  0.8530871871611367
  0.0034471656887831  0.5020929824871274  0.8531460201853136
  0.5026402484339982  0.0022464062603262  0.8532092999919243
  0.5030740402333436  0.5047550306775150  0.8591210952774233
  0.3400568505546673  0.1648619696705964  0.6900013231141244
  0.3361983810433173  0.6672334996491500  0.6868763544896055
  0.8357428660133834  0.1678046161236750  0.6863627427944294
  0.8370873169210427  0.6688571277887263  0.6869903996726876
  0.3365360786053699  0.1686783948707031  0.9786725957976827
  0.3350057934617610  0.6695718188093451  0.9787993520434100
  0.8375957449887252  0.1698093618409544  0.9785360151103220
  0.8366944903110607  0.6703663413203884  0.9783737114240409
  0.3265126843519752  0.1487831669627298  0.4696856689919997
  0.3329975787028516  0.6714203309550868  0.4752597984186409
  0.8322281911906657  0.1651293590646929  0.4777401896103299
  0.8349736002288779  0.6673169457764203  0.4784937362775850
  0.3349551240026543  0.1685052096020527  0.1858218419440263
  0.3352412715260940  0.6686393866474987  0.1853574647597256
  0.8360857971309628  0.1690868868886213  0.1858331331222658
  0.8345466684685476  0.6679844662548859  0.1855821320049458
  0.1691385502045790  0.3352475453300343  0.0192423242948294
```

0.1691129528396615	0.8362027906183442	0.0195250598317642
0.6697063365877796	0.3355431139516938	0.0202098196262810
0.6697211795763991	0.8366026283642967	0.0216266658362727
0.1681801535737222	0.3362341216897988	0.3103005859936658
0.1667988474327302	0.8324816206653907	0.3097781654432623
0.6689490207628808	0.3353960678045809	0.3114645425226001
0.6700336097412105	0.8354885561818648	0.3112274931512573
0.1721954689894076	0.3382281476125399	0.8109550149651028
0.1679837446982546	0.8338807321966186	0.8126916747623838
0.6712545717827183	0.3350590992083013	0.8134876344429287
0.6717001131907736	0.8419452874745588	0.8157690607764323
0.1489668843301004	0.3331587290150111	0.5183350010727565
0.1665585719748250	0.8322811127185616	0.5203461548007624
0.6717633482074632	0.3344627091964654	0.5201143308880489
0.6677317128928308	0.8333254407105031	0.5187790504393703
0.1555898143828074	0.0007904186560808	0.2482708698598799
0.1533427650401649	0.5018139396557544	0.2464379030294310
0.6559422429114861	0.0026589324246586	0.2486565359739572
0.6542769540128290	0.5008898567964835	0.2484868910825568
0.3492411416556891	0.0004061196818057	0.7500475154958153
0.3532168472558439	0.5080658154515802	0.7547456720857113
0.8516339923756959	0.0043358670569020	0.7514053127840299
0.8504745162861056	0.5018206554004365	0.7503198320231661
0.0024037054249888	0.1543800967343429	0.2475988197450434
0.0005932980839063	0.6544355949408427	0.2478496433855000
0.5010588926647088	0.1558775691988359	0.2497739182177128
0.5049128686631192	0.6579338225086886	0.2456204977896093
0.0037025640843851	0.3490205967545350	0.7487141876421504
0.0078556946732959	0.8539116320033955	0.7481216527138841
0.5035164882552863	0.3463840967187863	0.7527216925892759
0.5038311100821602	0.8494102372959347	0.7488565366290985
0.3483283154867678	0.3491203391021855	0.2470774634805295
0.3485217789622865	0.8480374801031800	0.2477199778635466
0.8452831217230496	0.3484710977223671	0.2462005050186349
0.8491240311486550	0.8480508610033937	0.2486078713645838
0.1624962695272529	0.1627817825655384	0.7444856612024253
0.1567662296443260	0.6559259910866757	0.7502271426477876
0.6549637777703978	0.1550879829017617	0.7485073999603955
0.6743126278135421	0.6677289050247595	0.7645523182585094
0.4835041539435352	0.1723171650282467	0.5823741170362666
0.4855103342623437	0.6626893621877130	0.5810865878535125
0.9732165517808653	0.1546612634887055	0.5833181589539436
0.9941062322380816	0.6719172823271862	0.5836232061211817
0.1824871877049787	0.1684812544069558	0.0820551459153549
0.1823121242694512	0.6694765435405898	0.0817700435493549
0.6823821533131635	0.1687239251668373	0.0824276980411796
0.6829538021082584	0.6689468915255006	0.0824183071209177
0.3364499146594113	0.8249832582559335	0.5812466697257292
0.8370536697784914	0.3210165706629340	0.5821221094390765
0.8329532230765722	0.8195828458885970	0.5836016672014424

0.3357098045334439	0.0157288687702604	0.0823070743852128
0.3353872882192945	0.5156788906423060	0.0819245896605594
0.8361162343038799	0.0166917262048045	0.0831493190762416
0.8360748632102428	0.5162994025277499	0.0818525281752187
0.1878041112766340	0.0258641120276550	0.5800728324967892
0.1836445540710585	0.5224226744509366	0.5833212874090137
0.6757574042664984	0.0109659107678155	0.5810036497831490
0.6968875148253559	0.5171513563899381	0.5814692901236640
0.4890958373758423	0.3215081408212797	0.0823303429893514
0.4888251201382090	0.8231199306981551	0.0825901351434055
0.9889879403050443	0.3219579320436949	0.0818303018648550
0.9897092908040790	0.8221407511457599	0.0826259641277858
0.3228323825461388	0.3355723386517724	0.9171858009651928
0.3227866898837064	0.8362190939032885	0.9160120149781790
0.8233779315059238	0.3354996992374666	0.9160927127380205
0.8273999539710388	0.8418048979166812	0.9201434421309145
0.0016879039354336	0.3276000078479910	0.4069207998508944
0.0161631705355632	0.8359759023527857	0.4162805208790132
0.5071261134212008	0.3378060052644045	0.4207183094883291
0.5160555504661701	0.8354328691290647	0.4142388486022526
0.1689641322096946	0.4891296369514890	0.9166664591899334
0.1705266390776288	0.9901915498066742	0.9152348471154212
0.6694282467217505	0.4878501940286171	0.9183534089649470
0.6694916918693039	0.9907578212876871	0.9169327179782698
0.1703296654624822	0.1800511353519880	0.4125533404533002
0.1644683553731454	0.6803062825797426	0.4145444829302889
0.6635110915099887	0.1814981309999197	0.4161329034806514
0.6675767149014504	0.6802841701269529	0.4152342363469614
0.0176805282119830	0.1835205218228921	0.9152230425381731
0.0160695987670962	0.6830128825536335	0.9155829387581775
0.5164080467879373	0.1812863991409088	0.9165848259276501
0.5172987437791932	0.6851953912493229	0.9192923837580480
0.3237346597597295	0.4994330051324241	0.4065115566659756
0.3214195303984582	0.9801981695316400	0.4120775122175431
0.8194063716588076	0.4870890727804777	0.4137745822880916
0.8203716048054375	0.9857518267582961	0.4147605941938934
0.3758931483075543	0.3795839242813699	0.5662988921658467
0.4017461283019514	0.4967338269252650	0.5582628484291448
0.4448732687573128	0.3818490376786060	0.5096536736192967

Table S19: Gibbs free energy calculation at 400 °C.

No.	State	DFT free energy [eV]	ZPE [eV]	U [eV]	S [meV/K]	Δ G [eV]
1	Fe_2O_3 (bulk)	-839.745	0.083	-839.557	0.28	-839.749
2	H_2O (gas)	-14.217	0.568	-13.410	2.31	-14.966
3	H_2 in IS	-844.746	0.577	-843.941	0.62	-844.359
4	2H split in IS	-845.660	0.560	-844.932	0.43	-845.218
5	O-vac	-831.602	-	-831.602	-	-831.602
6	H_2O in IS	-849.983	0.801	-848.917	0.68	-849.377
7	H_2O in IS with O-vacancy	-845.341	0.663	-844.515	0.41	-844.793
